# Organocatalyzed Enantioselective [3+2] Cycloaddition Reactions for Synthesis of Dispiro[benzothiophenone-indandione-pyrrolidine] Derivatives

**DOI:** 10.3390/molecules29204856

**Published:** 2024-10-13

**Authors:** Hong-Yan Liu, Da-Ming Du

**Affiliations:** 1 School of Chemistry and Chemical Engineering, Beijing Institute of Technology, No. 5 Zhongguancun South Street, Beijing 100081, China; 3220211472@bit.edu.cn; 2 Key Laboratory of Medicinal Molecule Science and Pharmaceutical Technology, Ministry of Industry and Information Technology, No. 5 Zhongguancun South Street, Beijing 100081, China

**Keywords:** asymmetric catalysis, 2-arylidene-1,3-indandione, benzothiophenone, thiourea, squaramide, spirocyclic compounds

## Abstract

An organocatalytic enantioselective [3+2] cycloaddition reaction involving 2-arylidene-1,3-indandiones and *N*-2,2-difluoroethylbenzothiophenone imines was developed. This approach efficiently afforded dispiro[benzothiophenone-indandione-pyrrolidine]s, featuring three stereocenters, in 84–98% yields with 3–93% ee and 9:1–>20:1 dr. Notably, the method maintained its yield and enantioselectivity integrity even in a gram-scale amplification experiment. For example, the product with substituents on aromatics were obtained in 90% yield with 91% ee and >20:1 dr. Its absolute configuration was established through X-ray single-crystal diffraction analysis, and a plausible reaction mechanism was proposed.

## 1. Introduction

**Figure 1:**
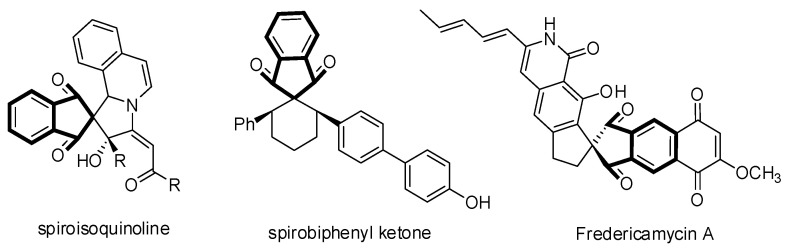
Medicines containing chiral spiroindanone skeletons.

In recent years, chiral spiroindanone compounds have garnered significant attention due to their remarkable bioactivities exhibiting numerous potential pharmacological effects. Among them, spiroisoquinolines have attracted particular interest for their sedative, antihypertensive, and neuromuscular blocking activities. Bisphenyl spiroketones, on the other hand, are emerging as novel anticancer agents, offering new directions for the development of anticancer drugs. Moreover, Fredericamycin A, a notable antitumor compound with significant antibiotic properties, has demonstrated immense potential in clinical medicine [1,2,3] (Figure 1). Given the immense value of chiral spiroindanone compounds in the fields of clinical medicine and medicinal chemistry, their construction has remained a hotspot in asymmetric catalytic synthesis.

**Figure 2:**
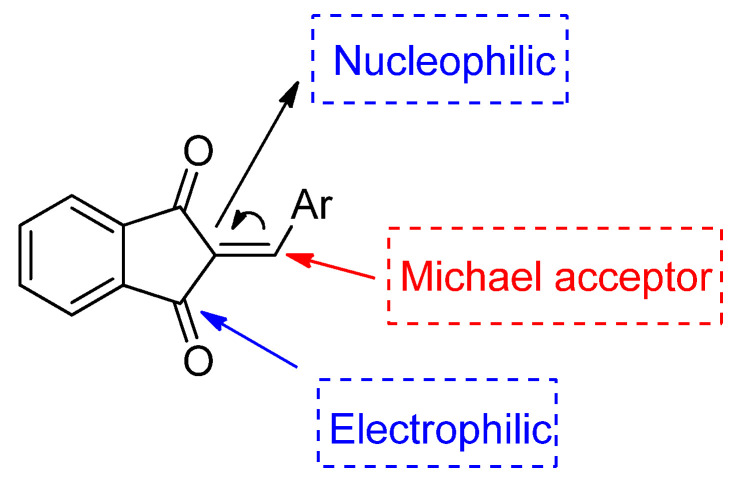
The reactive sites of 2-arylidene-1,3-indandione.

**Scheme 1:**
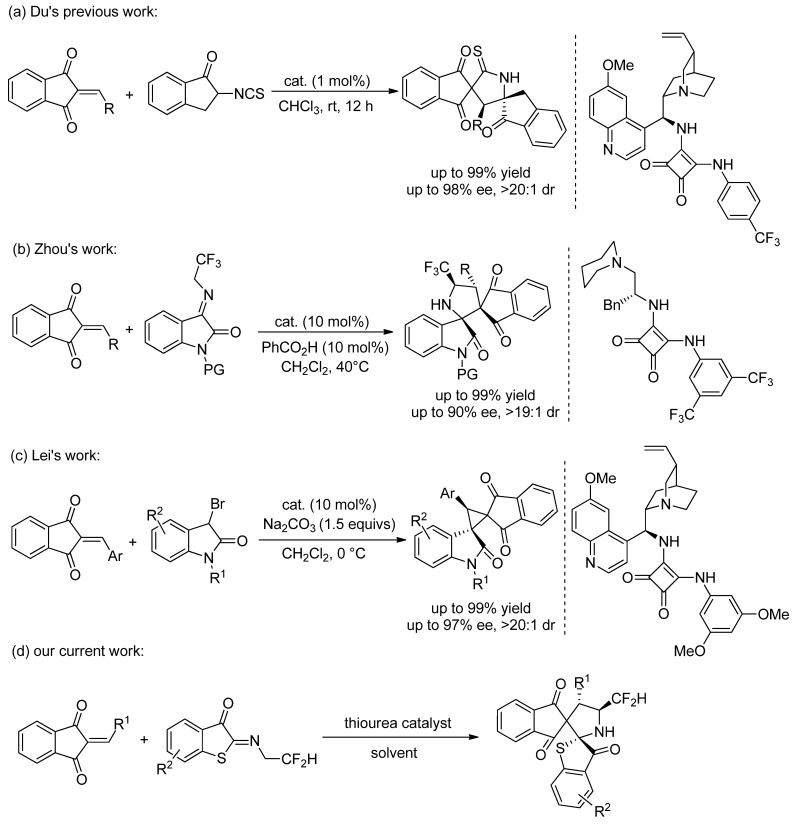
Previous reports and our current work. (**a**) [3+2] annulation by Du, et al. [16]; (**b**) [3+2] annulation by Zhou, et al. [17]; (**c**) [2+1] cycloaddition by Lei, et al. [18]; (**d**) our research plan.

2-Arylidene-1,3-indandione, a planar compound, features both its indandione and arylidene moieties as potential electrophilic centers susceptible to nucleophilic attack, and the central carbon can be transformed to nucleophilic in reaction progress (Figure 2). Its 1,3-indandione scaffold bears three consecutive reactive sites, enabling self-condensation to form homodimers and cyclotrimers. The electrophilicity of its arylidene part renders it a Michael acceptor, participating in annulation reactions to yield six-membered rings, enhancing structural diversity. Furthermore, it undergoes dipolar cycloaddition with 1,3-dipoles, yielding five-membered rings. Toxicity modulation is achievable by introducing diverse polar groups onto the aryl ring [4,5,6,7]. These properties make 2-arylidene-1,3-indandione highly sought-after in synthetic chemistry and drug development. Notably, it also demonstrates promising prospects in constructing biologically active spirocyclic scaffolds using nucleophilicity of the central carbon and allowing the [3+2] cycloaddition [8,9,10,11,12,13,14,15]. For example, in 2022, our group achieved an asymmetric [3+2] cyclization reaction between 2-arylidene-1,3-indanediones and isothiocyanatoindanones catalyzed by squaramide in chloroform, and thiodispiro[indene-pyrrolidine-indene]-trione compounds with two consecutive stereogenic centers were obtained [16] (Scheme 1a). This reaction exhibited high yields and excellent stereoselectivities. In 2023, Zhou and coworkers delved into a mild and efficient organocatalytic [3+2] cycloaddition reaction between isatin-derived ketimines and 2-ylideneindane-1,3-diones [17] (Scheme 1b), spiro[oxindole-3,2′-pyrrolidine]s compounds that integrate both the spiroindane-1,3-dione motif and trifluoromethyl, two crucial structural units were successfully constructed. This reaction proceeded with high yields and displayed good diastereoselectivities and enantioselectivities. Moreover, in 2023, Lei and his team successfully utilized squaramide-catalyzed [2+1] cycloaddition to synthesize a series of compounds featuring a dispiro[indanedione-oxindole-cyclopropane] scaffold [18] (Scheme 1c), starting from 2-arylidene-1,3-indanediones and 3-bromooxindoles. This reaction system proceeded through an efficient tandem Michael/alkylation pathway, and the products were obtained with excellent diastereoselectivities and enantioselectivities.

Furthermore, the introduction of fluorine groups as an effective molecular modification approach profoundly impacts the physical, chemical, biological, and pharmacokinetic properties of organic molecules [19]. Notably, the selective replacement of hydrogen atoms in methyl groups with fluorine atoms to form difluoromethyl groups (–CF_2_H) exerts a pivotal role in drug-biological target interactions due to their weak acidity as hydrogen bond donors [20]. Molecules containing the –CF_2_H group exhibit significant advantages in enhancing drug efficacy, attributed to their heightened binding affinity [21]. However, given the pronounced differences in properties between –CF_3_ and –CF_2_H, studies on 1,3-dipoles featuring –CF_2_H are relatively scarce, motivating our dedication to exploring this area.

Inspired by this, herein, we developed an organocatalyzed enantioselective [3+2] cycloaddition to construct chiral dispiro[benzothiophenone-indandione-pyrrolidine] compounds (Scheme 1d) using 2-arylidene-1,3-indandiones and 2-((2,2-difluoroethyl)imino)benzo[*b*]thiophen-3(2*H*)-ones, aiming to contribute efficient and safe new drug candidates to the pharmaceutical research field through this innovative strategy.

## 2. Results and Discussion

### 2.1. Optimization of Reaction Conditions

**Figure 3:**
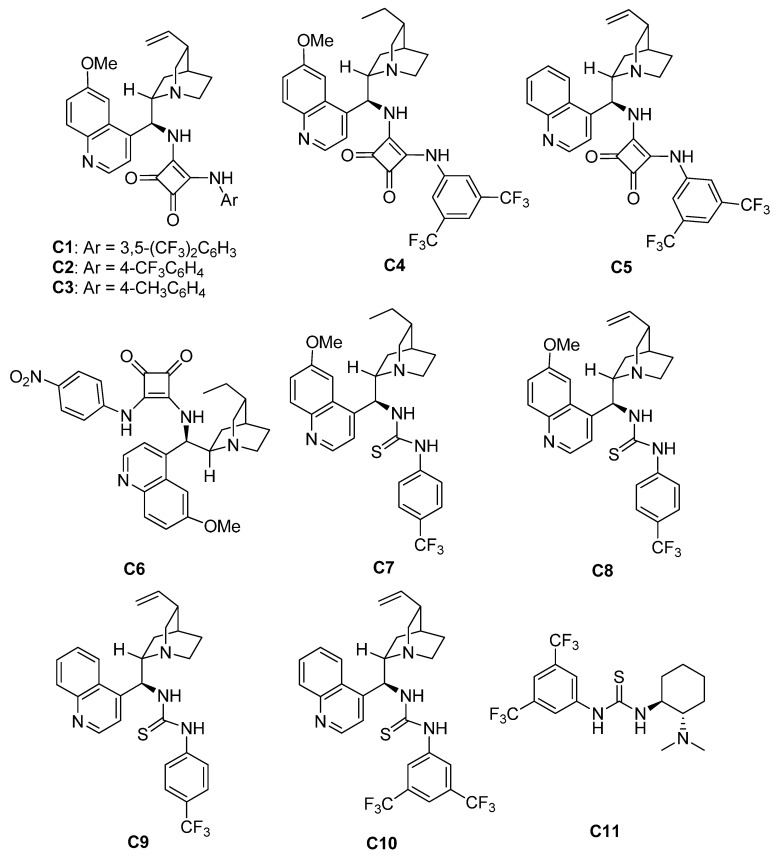
Organocatalysts selected.

Using 2-arylidene-1,3-indandione **1a** and 2-((2,2-difluoroethyl)imino)benzo[*b*]thiophen-3(2*H*)-one **2a** as the template substrates, we embarked on a systematic investigation to identify the optimal reaction parameters through a rigorous evaluation of various reaction conditions. The objective was to establish a robust synthetic protocol for the target compounds, backed by sound experimental data. Initially, dichloromethane served as the solvent medium, using 10 mol% of quinine-derived bis(trifluoromethyl)-substituted squaramide **C1** as catalyst (depicted in Figure 3). The reaction was carried out at ambient temperature over a period of 24 h. Subsequent analysis revealed that these conditions facilitated the formation of a dispiro[benzothiophenone-pyrrolidine-indandione] derivative in 80% yield with 20:1 dr and 76% ee. Having validated the identity of the desired product, we proceeded to evaluate the catalytic efficacy of alternative catalysts under identical experimental settings. The outcomes of these experiments are concisely presented in Table 1, facilitating a comparative assessment of catalyst performance.

Through screening of quinine-derived squaramide catalysts, it was found that catalyst **C2** had similar catalytic effects as **C1**, and although the yield of **3aa** was improved using **C3**, its enantiomeric excess decreased significantly. After using hydroquinine-derived squaramide **C4** and cinchonidine-derived squaramide catalyst **C5**, the enantiomeric excess did not improve. Subsequently, the use of hydroquinidine-derived catalyst **C6** did not exhibit good catalytic effects. Therefore, attention was turned to thiourea catalysts **C7**–**C11**, with the hope of finding a more effective catalytic system. Through experimental attempts, it was pleasantly surprised to find that the 4-trifluoromethylphenyl-substituted thiourea catalyst **C9** exhibited excellent enantiomeric excess in this reaction. To more comprehensively evaluate the performance of different catalysts, quinine and cyclohexanediamine catalysts were also tested and compared with the thiourea catalysts. After careful comparison, **C9** was ultimately selected as the optimal catalyst for this experiment, as it exhibited good catalytic activity while enhancing enantiomeric excess.

After determining the optimal catalyst as **C9**, further screenings of solvents, catalyst loading, and reaction temperature were conducted to optimize the reaction conditions. Initially, eight different solvents including chloroform, 1,2-dichloroethane (DCE), toluene, acetonitrile, tetrahydrofuran (THF), methyl *tert*-butyl ether (MTBE), dioxane, and diethyl ether were tested (Table 1, entries 9, 12–19). Under identical experimental conditions, it was found that dichloromethane remained the best solvent for this reaction, providing the highest yield and enantioselectivity. Subsequently, the influence of catalyst loading and temperature on the reaction was investigated. The experimental results showed that neither increasing nor decreasing the catalyst loading significantly improved the enantioselectivity. This indicated that 10 mol% catalyst loading of the **C9** was already sufficiently effective in the current reaction system. Then, the reaction temperature was lowered to −10 °C. Although the enantioselectivity of the reaction was well maintained at this temperature, the reaction time significantly increased and the yield decreased. Therefore, considering the balance between yield and enantioselectivity, it was decided to maintain room temperature as the reaction temperature.

In summary, the optimal reaction conditions for this asymmetric [3+2] cycloaddition reaction of **1a** and **2a** were established as: 10 mol% of 4-trifluoromethylphenyl-substituted thiourea **C9** as the catalyst, 2 mL of dichloromethane as the solvent, and the reaction was conducted at room temperature.

### 2.2. Substrate Scope

**Scheme 2:**
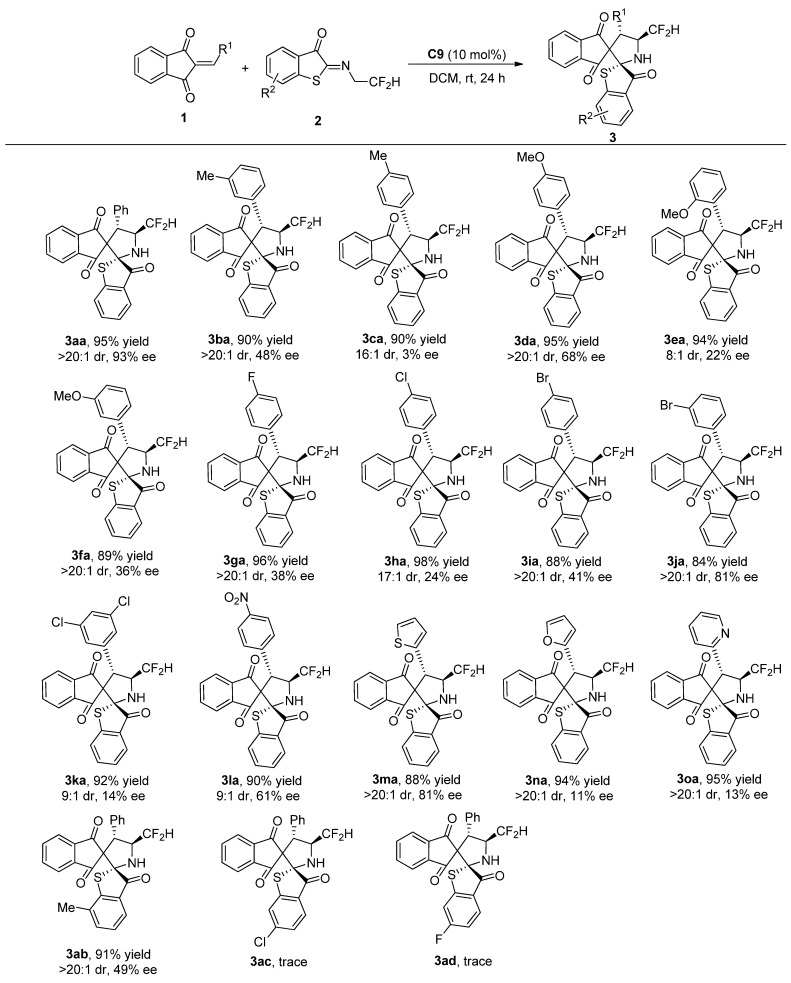
Substrate scope for dispiro[benzothiophenone-indandione-pyrrolidine]. **3**. The reactions were carried out with **1** (0.10 mmol), **2** (0.10 mmol), and catalyst **C9** (10 mol%) in DCM (2.0 mL) at room temperature for 24 h (48 h for **3ac** and **3ad**). Product yield was determined after purification on column chromatography. The dr values were determined by ^1^H NMR, and the ee values were determined by HPLC analysis.

Under the optimized reaction conditions, a systematic study was conducted on the substrate scope of the asymmetric [3+2] cycloaddition reaction, and the results are summarized in Scheme 2.

Firstly, the focus was placed on the substrate diversity of 2-arylidene-1,3-indandione. Experimental data indicated that the reaction exhibited high yields (84−98% yield) in most cases, yet the enantioselectivity was influenced by electronic effects. Specifically, when the substituent was a methyl group with electron-donating properties, the *meta*- and *para*-substitutions significantly affected the enantioselectivity of the products. Compared to the *para*-substituted product **3ca**, the *meta*-substituted product **3ba** exhibited significantly better enantioselectivity. Furthermore, when the methyl group was replaced by a methoxy group with a stronger electron-donating effect, the *ortho*- and *meta*-substituted products showed relatively poor enantioselectivity (22−36% ee), while the *para*-substituted methoxy product **3da** exhibited relatively better enantioselectivity (68% ee).

Subsequently, the influence of electron-withdrawing groups on the reaction was further investigated. The experimental results showed that electron-withdrawing groups such as fluorine, chlorine, and bromine substituted at the *para*-position exhibited relatively poor tolerance to the reaction, and the resulting products **3ga**–**3ia** generally had low enantioselectivity (24−41% ee). However, when the bromine atom was located at the *meta*-position, although the yield slightly decreased (84% yield), the enantioselectivity significantly improved (81% ee). This finding encouraged us to explore more *meta*-substituted substrates; unfortunately, the experimental result for substrate bearing *meta*-disubstituted chlorine atoms was not ideal (14% ee). When using *para*-nitro-substituted substrate, the enantioselectivity of product **3la** was moderate (51% ee). In addition, substrates with different heterocyclic substituents were also evaluated. Among the heterocyclic substituted substrates such as thiophene, furan, and pyridine, the thiophene-substituted product **3ma** exhibited relatively ideal results (88% yield and 81% ee).

To further explore the substrate scope of the asymmetric tandem reaction, the research focus was shifted to the substrate diversity of *N*-2,2-difluoroethylbenzothiophenone imine **2**. The experimental results indicate that when *N*-2,2-difluoroethylbenzothiophenone imine bearing an electron-donating methyl group, the reaction proceeded smoothly, generating the corresponding product **3ba**. The reaction still maintained a good yield (91% yield), but in terms of enantioselectivity, the performance of product **3ba** was relatively moderate, only reaching 49% ee. Subsequently, the reaction substrates were expended to *N*-2,2-difluoroethylbenzothiophenone imine **2** bearing electron-withdrawing groups. The experimental results show that when introducing electron-withdrawing groups such as chlorine and fluorine atoms, the reaction did not produce the corresponding target products. This finding indicates that electron-withdrawing groups have a significant inhibitory effect on the reaction, leading to the inability to obtain the expected product.

### 2.3. Scaled-Up Synthesis

**Scheme 3:**
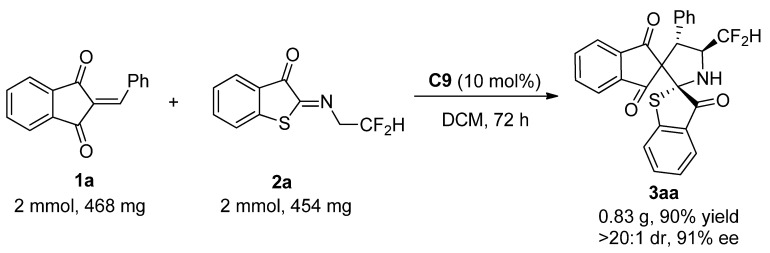
Scaled-up synthesis of **3aa**.

To validate the synthetic practicability and potential of this asymmetric [3+2] cycloaddition reaction strategy, a scale-up experiment was conducted under optimized conditions (as shown in Scheme 3). The experimental results demonstrated that even with the enlarged reaction scale, the strategy remained highly efficient and maintained excellent yield and stereoselectivity (90% yield, >20:1 dr, 91% ee). This result showcases the remarkable application value of this strategy in the large-scale asymmetric synthesis of such dispirocyclic benzothiophenone-indandione-pyrrolidine derivatives, providing strong potential for future practical applications and drug development.

### 2.4. X-ray Diffraction Analysis

**Figure 4:**
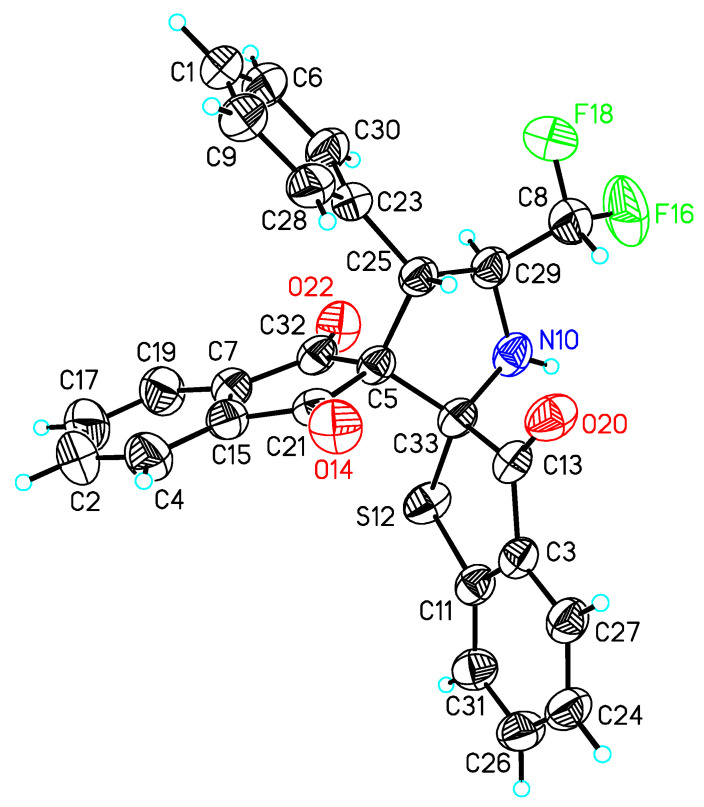
X-ray crystal structure of **3aa**.

The single crystal of **3aa** was obtained by slowly evaporating the solvent from ethyl acetate, and the absolute configuration of compound **3aa** was determined to be (2*S*,4′*S*,5′*S*) (see Supplementary Materials) through single-crystal X-ray diffraction technology (Figure 4) [22]. The absolute configurations of other products were determined by analogy.

### 2.5. Plausible Mechanism

**Scheme 4:**
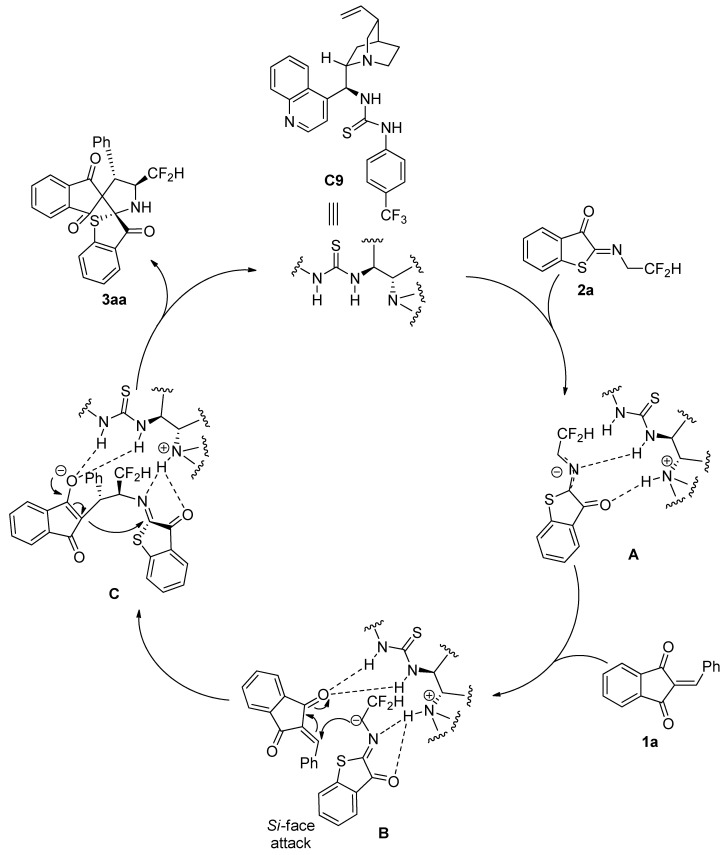
Proposed reaction mechanism.

Based on the previous work, we proposed a reasonable catalytic reaction mechanism (Scheme 4). In the initial stage of the Michael addition, the catalyst **C9** activates the *N*-2,2-difluoroethylbenzothiophenone imine **2a** through protonation, thereby facilitating the formation of the transition state **A**. Subsequently, **1a** and **2a** are effectively activated under the hydrogen bonding interaction of the catalyst **C9**. Immediately thereafter, the deprotonated **2a** attacks the 2-aryl-1,3-indandione **1a** from the *Si*-face, traversing through the transition state **B** to complete the intermolecular Michael addition. Following this, the resulting 2-aryl-1,3-indandione anion serves as a nucleophile in the Mannich cyclization reaction, attacking the *C=N* double bond of the benzothiophenone imine via the transition state **C**. Through this crucial step, the observed dispirocyclic product **3aa** is ultimately formed. Concurrently, the deprotonation process also occurs, enabling the regeneration of the bifunctional catalyst **C9**. Alternatively, a concert 1,3-dipolar [3+2] cycloaddition mechanism may also be possible for this asymmetric catalytic reaction.

## 3. Materials and Methods

### 3.1. General Information

Commercially sourced compounds were used as received without any additional purification. Solvents were dried following commonly used drying methods. The isolation of products was performed on column chromatography utilizing silica gel (200–300 mesh). Melting points were ascertained with a WRX-4 melting point apparatus and without correction. ^1^H NMR spectra were acquired on a Bruker Ascend 400 MHz spectrometer (Bruker, Karlsurhe, Germany), and the chemical shifts were reported in δ (ppm), using tetramethylsilane (TMS) as the internal standard. ^13^C NMR spectra were measured at 100 MHz with a 400 MHz spectrometer, and the chemical shifts were referenced to tetramethylsilane and the deuterated solvent peak (CDCl_3_, δC = 77.00 ppm; DMSO-d_6_, δC = 39.43 ppm). High-resolution mass spectra were measured with an Agilent 6520 Accurate-Mass Q-TOF MS equipment (Agilent, Santa Clara, CA, USA) using an electrospray ionization system (ESI^+^). Optical rotations of chiral products were measured with a Krüss P8000 polarimeter (KRÜSS, Hamburg, Germany), and the concentration unit was reported as g/100 mL. The enantiomeric excesses of chiral products were determined with chiral HPLC analysis using an Agilent 1200 LC instrument (Agilent, Santa Clara, CA, USA) on a Daicel Chiralpak IC, or AD-H column.

### 3.2. Materials

2-Arylidene-1,3-indandiones **1a**–**1o** were prepared according to the literature reported by Yang et al. [23], and *N*-2,2-difluoroethylbenzothiophenone imines **2a**–**2d** were prepared according to the literature by Sun et al. [24]. The chiral organocatalysts were prepared following the procedures previously reported [25,26,27,28,29].

### 3.3. Procedure for the Asymmetric Synthesis of Compounds ***3***

2-Arylidene-1,3-indandione **1** (0.10 mmol), *N*-2,2-difluoroethylbenzothiophenone imine **2** (0.10 mmol), organocatalyst **C9** (4.8 mg, 0.01 mmol, 10 mol%), and CH_2_Cl_2_ (2.0 mL) were sequentially added to a small, dried glass bottle. The reaction mixture was stirred at room temperature for 24 h. After completion of the reaction, the solvent was evaporated under reduced pressure, and the residue was purified by silica gel (200–300 mesh) flash column chromatography using ethyl acetate/petroleum ether (1:7) as eluent. The pure products **3** were obtained. The corresponding racemate sample was prepared following a similar procedure with Et_3_N (10 mol%).

(2*S*,4′*S*,5′*S*)-5′-(Difluoromethyl)-4′-phenyl-3*H*-dispiro[benzo[*b*]thiophene-2,2′-pyrrolidine-3′,2″-indene]-1″,3,3″-trione (**3aa**). From **1a** (23.4 mg, 0.10 mmol) and **2a** (22.7 mg, 0.10 mmol), compound **3aa** (43.8 mg, 95% yield) was obtained as a white solid, m.p. 215–217 °C. HPLC (Daicel Chiralpak IC column, mobile phase *n*-hexane/2-propanol = 70:30, flow rate 1.0 mL/min, detection wavelength 254 nm): *t*_R_ = 9.5 min (minor), *t*_R_ = 12.0 min (major); 93% ee. [α]_D_^25^ = −8.1 (*c* = 0.54, CH_2_Cl_2_). ^1^H NMR (400 MHz, CDCl_3_): *δ* 7.83 (d, *J* = 7.6 Hz, 1H, ArH), 7.79 (d, *J* = 7.2 Hz, 1H, ArH), 7.69–7.60 (m, 3H, ArH), 7.45–7.41 (m, 1H, ArH), 7.22 (t, *J* = 7.6 Hz, 1H, ArH), 7.10 (d, *J* = 7.2 Hz, 2H, ArH), 7.06–6.98 (m, 4H, ArH), 6.25 (td, *J*_1_ = 56.8, *J*_2_ = 6.8 Hz, 1H, CF_2_H), 4.82 (d, *J* = 10.0 Hz, 1H, CH), 4.72–4.63 (m, 1H, CH), 3.36 (br s, 1H, NH) ppm. ^13^C NMR (100MHz, CDCl_3_): *δ* 200.4, 198.7, 196.1, 147.4, 142.5, 142.1, 136.3, 136.1, 135.8, 132.7, 129.1, 128.5, 128.4, 127.9, 127.6, 125.9, 123.5, 123.21, 123.17, 118.1 (t, ^1^*J*_C–F_ = 241.9 Hz), 81.2, 71.2, 62.2 (dd, ^2^*J*_C–F_ = 27.3, 21.3 Hz), 51.9 (d, ^3^*J*_C–F_ = 7.6 Hz) ppm. ^19^F NMR (376 MHz, CDCl_3_): *δ* −117.2 (d, *J* = 288.4 Hz, 1F), −121.8 (d, *J* = 288.8 Hz, 1F) ppm. HRMS (ESI^+^): *m*/*z* calculated for C_26_H_18_F_2_NO_3_S [M + H]^+^ 462.0970, found 462.0957.

(2*S*,4′*S*,5′*S*)-5′-(Difluoromethyl)-4′-(*m*-tolyl)-3*H*-dispiro[benzo*[b*]thiophene-2,2′-pyrrolidine-3′,2″-indene]-1″,3,3″-trione (**3ba**). From **1b** (24.8 mg, 0.10 mmol) and **2a** (22.7 mg, 0.10 mmol), compound **3ba** (42.8 mg, 90% yield) was obtained as a white solid, m.p. 193–195 °C. HPLC (Daicel Chiralpak ADH column, mobile phase *n*-hexane/2-propanol = 70:30, flow rate 1.0 mL/min, detection wavelength 254 nm): *t*_R_ = 11.9 min (major), *t*_R_ = 23.2 min (minor); 48% ee. [α]_D_^25^ = −189.6 (*c* = 0.75, CH_2_Cl_2_). ^1^H NMR (400 MHz, CDCl_3_): *δ* 7.83 (d, *J* = 7.2 Hz, 1H, ArH), 7.78 (d, *J* = 7.6 Hz, 1H, ArH), 7.69–7.59 (m, 3H, ArH), 7.45–7.40 (m, 1H, ArH), 7.21 (t, *J* = 7.6 Hz, 1H, ArH), 7.03 (d, *J* = 8.0 Hz, 1H, ArH), 6.95–6.86 (m, 3H, ArH), 6.80 (d, *J* = 6.8 Hz, 1H, ArH), 6.24 (td, *J*_1_ = 56.8 Hz, *J*_2_ = 6.5 Hz, 1H, CF_2_H), 4.78 (d, *J* = 9.6 Hz, 1H, CH), 4.69–4.61 (m, 1H, CH), 3.35 (br s, 1H, NH), 2.11 (s, 3H, CH_3_) ppm. ^13^C NMR (100 MHz, CDCl_3_): *δ* 200.5, 198.7, 196.2, 147.4, 142.5, 142.2, 138.0, 136.2, 136.1, 135.7, 132.6, 129.2, 129.1, 128.7, 128.3, 127.6, 125.9, 125.4, 123.5, 123.12, 123.05, 118.1 (t, ^1^*J*_C–F_ = 242.0 Hz), 81.2, 71.2, 62.1 (dd, ^2^*J*_C–F_ = 27.2, 21.4 Hz), 51.9 (d, ^3^*J*_C–F_ = 7.5 Hz), 21.2. ^19^F NMR (376 MHz, CDCl_3_): *δ* −117.2 (d, *J* = 288.4 Hz, 1F), −121.7 (d, *J* = 288.0 Hz, 1F) ppm. HRMS (ESI^+^): *m*/*z* calculated for C_27_H_20_F_2_NO_3_S [M + H]^+^ 476.1127, found 476.1118.

(2*S*,4′*S*,5′*S*)-5′-(Difluoromethyl)-4′-(*p*-tolyl)-3*H*-dispiro[benzo[*b*]thiophene-2,2′-pyrrolidine-3′,2″-indene]-1″,3,3″-trione (**3ca**). From **1c** (24.8 mg, 0.10 mmol) and **2a** (22.7 mg, 0.10 mmol), compound **3ca** (42.8 mg, 90% yield) was obtained as a white solid, m.p. 164–165 °C. HPLC (Daicel Chiralpak ADH column, mobile phase *n*-hexane/2-propanol = 70:30, flow rate 1.0 mL/min, detection wavelength 254 nm): *t*_R_ = 13.9 min (major), *t*_R_ = 29.7 min (minor); 3% ee. [α]_D_^25^ = −128.2 (*c* = 0.29, CH_2_Cl_2_). ^1^H NMR (400 MHz, CDCl_3_): *δ* 7.81 (t, *J* = 7.4 Hz, 2H, ArH), 7.70–7.60 (m, 3H, ArH), 7.42 (td, *J*_1_ = 7.6 Hz, *J*_2_ = 1.2 Hz, 1H, ArH), 7.21 (t, *J* = 7.2 Hz, 1H, ArH), 7.02 (d, *J* = 7.6 Hz, 1H, ArH), 6.98 (d, *J* = 8.4 Hz, 2H, ArH), 6.84 (d, *J* = 8.0 Hz, 2H, ArH), 6.23 (dd, *J*_1_ = 56.8, *J*_2_ = 6.8 Hz, 1H, CF_3_H), 4.78 (d, *J* = 9.6 Hz, 1H, CH), 4.68–4.60 (m, 1H, CH), 3.32 (br s, 1H, NH), 2.10 (s, 3H, CH_3_) ppm. ^13^C NMR (100 MHz, CDCl_3_): *δ* 200.4, 198.8, 196.2, 147.4, 142.6, 142.2, 137.6, 136.2, 136.1, 135.8, 134.5, 129.64, 129.59, 129.2, 128.3, 127.6, 125.9, 123.5, 123.23, 123.17, 118.1 (t, ^1^*J*_C–F_ = 241.8 Hz), 81.3, 71.1, 62.3 (dd, ^2^*J*_C–F_ = 27.2, 21.2 Hz), 51.5 (d, ^3^*J*_C–F_ = 7.5 Hz), 20.9 ppm. ^19^F NMR (376 MHz, CDCl_3_): *δ* −117.1 (d, *J* = 288.4 Hz, 1F), −121.8 (d, *J* = 288.4 Hz, 1F) ppm. HRMS (ESI^+^): *m*/*z* calculated for C_27_H_20_F_2_NO_3_S [M + H]^+^ 476.1127, found 476.1126.

(2*S*,4′*S*,5′*S*)-5′-(Difluoromethyl)-4′-(4-methoxyphenyl)-3*H*-dispiro[benzo[*b*]thiophene-2,2′-pyrrolidine-3′,2″-indene]-1″,3,3″-trione (**3da**). From **1d** (26.4 mg, 0.10 mmol) and **2a** (22.7 mg, 0.10 mmol), compound **3da** (46.6 mg, 95% yield) was obtained as a white solid, m.p. 199–201 °C. HPLC (Daicel Chiralpak ADH column, mobile phase *n*-hexane/2-propanol = 70:30, flow rate 1.0 mL/min, detection wavelength 254 nm): *t*_R_ = 16.8 min (major), *t*_R_ = 32.9 min (minor); 68% ee. [α]_D_^25^ = −36.0 (*c* = 0.17, CH_2_Cl_2_). ^1^H NMR (400 MHz, CDCl_3_): *δ* 7.82 (t, *J* = 7.6 Hz, 2H, ArH), 7.71–7.61 (m, 3H, ArH), 7.43 (td, *J*_1_ = 7.6 Hz, *J*_2_ = 1.2 Hz, 1H, ArH), 7.23–7.19 (m, 1H, ArH), 7.03 (d, *J* = 8.0 Hz, 1H, ArH), 6.96 (t, *J* = 7.8 Hz, 1H, ArH), 6.69 (d, *J* = 7.6 Hz, 1H, ArH), 6.62 (s, 1H, ArH), 6.54 (dd, *J*_1_ = 8.2 Hz, *J*_2_ = 2.2 Hz, 1H, ArH), 6.24 (td, *J*_1_ = 56.6 Hz, *J*_2_ = 6.8 Hz, 1H, CF_2_H), 4.79 (d, *J* = 9.6 Hz, 1H, CH), 4.68–4.60 (m, 1H, CH), 3.63 (s, 3H, OCH_3_), 3.27 (br s, 1H, NH) ppm. ^13^C NMR (100 MHz, CDCl_3_): *δ* 200.5, 198.6, 196.1, 159.3, 147.5, 142.5, 142.2, 136.3, 136.2, 135.8, 134.3, 129.5, 129.1, 127.6, 125.9, 123.5, 123.2, 123.1, 120.7, 118.1 (t, ^1^*J*_C–F_ = 242.1 Hz), 113.9, 113.8, 81.2, 71.1, 62.2 (dd, ^2^*J*_C–F_ = 27.2, 21.4 Hz), 55.1, 51.9 (d, ^3^*J*_C–F_ = 7.5 Hz) ppm. ^19^F NMR (376 MHz, CDCl_3_): *δ* −117.3 (d, *J* = 288.4 Hz, 1F), −121.8 (d, *J* = 288.4 Hz, 1F) ppm. HRMS (ESI^+^): *m*/*z* calculated for C27H20F2NO4S [M + H]^+^ 492.1076, found 492.1078.

(2*S*,4′*S*,5′*S*)-5′-(Difluoromethyl)-4′-(2-methoxyphenyl)-3*H*-dispiro[benzo[*b*]thiophene-2,2′-pyrrolidine-3′,2″-indene]-1″,3,3″-trione (**3ea**). From **1e** (26.4 mg, 0.10 mmol) and **2a** (22.7 mg, 0.10 mmol), compound **3ea** (46.2 mg, 94% yield) was obtained as a white solid, m.p. 186–188 °C. HPLC (Daicel Chiralpak IC column, mobile phase *n*-hexane/2-propanol = 80:20, flow rate 1.0 mL/min, detection wavelength 254 nm): *t*_R_ = 10.6 min (major), *t*_R_ = 13.2 min (minor); 22% ee. [α]_D_^25^ = −2.1 (*c* = 0.49, CH_2_Cl_2_). ^1^H NMR (700 MHz, CDCl_3_): *δ* 7.90 (dd, *J*_1_ = 7.7 Hz, *J*_2_ = 0.8 Hz, 1H, ArH), 7.70 (d, *J* = 7.7 Hz, 1H, ArH), 7.63 (td, *J*_1_ = 7.7 Hz, *J*_2_ = 1.4 Hz, 1H, ArH), 7.59–7.55 (m, 2H, ArH), 7.45 (td, *J*_1_ = 7.7 Hz, *J*_2_ = 1.4 Hz, 1H, ArH), 7.27 (d, *J* = 7.7 Hz, 1H, ArH), 7.59–7.22 (m, 1H, ArH), 6.95 (td, *J*_1_ = 7.7 Hz, *J*_2_ = 1.4 Hz, 1H, ArH), 6.79 (td, *J*_1_ = 7.7 Hz, *J*_2_ = 0.7 Hz, 1H, ArH), 6.38–6.20 (m, 2H, ArH + CF_2_H), 5.22 (d, *J* = 9.8 Hz, 1H, CH), 4.71–4.65 (m, 1H, CH), 3.45 (s, 3H, CH_3_) ppm. ^13^C NMR (100 MHz, CDCl_3_): *δ* 201.5, 198.6, 194.9, 156.6, 147.7, 142.8, 141.1, 136.0, 135.43, 135.40, 129.4, 128.7, 128.4, 127.4, 125.7, 123.4, 122.8, 122.2, 121.1, 120.3, 118.1 (t, ^1^*J*_C–F_ = 241.7 Hz), 109.2, 80.4, 72.0, 61.5 (dd, ^2^*J*_C–F_ = 27.6, 21.5 Hz), 54.1, 45.4 (d, ^3^*J*_C–F_ = 7.5 Hz) ppm. ^19^F NMR (376 MHz, CDCl_3_): *δ* −117.1 (d, *J* = 287.2 Hz, 1F), −122.4 (d, *J* = 287.2 Hz, 1F) ppm. HRMS (ESI^+^): *m*/*z* calculated for C_27_H_20_F_2_NO_4_S [M + H]^+^ 492.1076, found 492.1073.

(2*S*,4′*S*,5′*S*)-5′-(Difluoromethyl)-4′-(3-methoxyphenyl)-3*H*-dispiro[benzo[*b*]thiophene-2,2′-pyrrolidine-3′,2″-indene]-1″,3,3″-trione (**3fa**). From **1f** (26.4 mg, 0.10 mmol) and **2a** (22.7 mg, 0.10 mmol), compound **3fa** (43.7 mg, 89% yield) was obtained as a white solid, m.p. 160–161 °C. HPLC (Daicel Chiralpak ADH column, mobile phase *n*-hexane/2-propanol = 70:30, flow rate 1.0 mL/min, detection wavelength 254 nm): *t*_R_ = 17.3 min (major), *t*_R_ = 38.0 min (minor); 36% ee. [α]_D_^25^ = −117.5 (*c* = 1.1, CH_2_Cl_2_). ^1^H NMR (400 MHz, CDCl_3_): *δ* 7.83–7.80 (m, 2H, ArH), 7.72–7.63 (m, 3H, ArH), 7.45–7.41 (m, 1H, ArH), 7.23–7.19 (m, 1H, ArH), 7.02 (d, *J* = 8.8 Hz, 3H, ArH), 6.58 (d, *J* = 8.8 Hz, 2H, ArH), 6.23 (td, *J*_1_ = 56.8 Hz, *J*_2_ = 6.8 Hz, 1H, CF_2_H), 4.77 (d, *J* = 9.6 Hz, 1H, CH), 4.65–4.57 (m, 1H, CH), 3.62 (s, 3H, OCH_3_), 3.33 (s, 1H, NH) ppm. ^13^C NMR (100 MHz, CDCl_3_): *δ* 200.5, 198.9, 196.3, 159.0, 147.5, 142.6, 142.2, 136.23, 136.18, 135.8, 129.6, 129.1, 127.6, 125.9, 124.6, 123.5, 123.23, 123.18, 118.1 (t, ^1^*J*_C–F_ = 242.1 Hz), 113.9, 81.3, 71.1, 62.4 (dd, ^2^*J*_C–F_ = 27.0, 21.3 Hz), 55.0, 51.2 (d, ^3^*J*_C–F_ = 7.5 Hz) ppm. ^19^F NMR (376 MHz, CDCl_3_): *δ* −117.0 (d, *J* = 288.4 Hz, 1F), −121.8 (d, *J* = 288.4 Hz, 1F) ppm. HRMS (ESI^+^): *m*/*z* calculated for C_27_H_20_F_2_NO_4_S [M + H]^+^ 492.1076, found 492.1081.

(2*S*,4′*S*,5′*S*)-5′-(Difluoromethyl)-4′-(4-fluorophenyl)-3*H*-dispiro[benzo[*b*]thiophene-2,2′-pyrrolidine-3′,2″-indene]-1″,3,3″-trione (**3ga**). From **1g** (25.2 mg, 0.10 mmol) and **2a** (22.7 mg, 0.10 mmol), compound **3ga** (46.0 mg, 96% yield) as a white solid, m.p. 167–170 °C. HPLC (Daicel Chiralpak ADH column, mobile phase *n*-hexane/2-propanol = 70:30, flow rate 1.0 mL/min, detection wavelength 254 nm): *t*_R_ = 13.7 min (major), *t*_R_ = 27.7 min (minor); 38% ee. [α]_D_^25^ = −186.0 (*c* = 0.23, CH_2_Cl_2_). ^1^H NMR (400 MHz, CDCl_3_): *δ* 7.82 (d, *J* = 7.6 Hz, 2H, ArH), 7.73–7.69 (m, 1H, ArH), 7.67–7.64 (m, 2H, ArH), 7.43 (t, *J* = 7.4 Hz, 1H, ArH), 7.21 (t, *J* = 7.6 Hz, 1H, ArH), 7.11–7.08 (m, 2H, ArH), 7.02 (d, *J* = 7.6 Hz, 1H, ArH), 6.75 (t, *J* = 8.4 Hz, 2H, ArH), 6.24 (td, *J*_1_ = 56.8 Hz, *J*_2_ = 6.8 Hz, 1H, CF_2_H), 4.81 (d, *J* = 10.0 Hz, 1H, CH), 4.67–4.58 (m, 1H, CH), 3.40 (br s, 1H, NH) ppm. ^13^C NMR (100 MHz, CDCl_3_): *δ* 200.3, 198.6, 196.0, 162.2 (^1^*J*_C–F_ = 245.7 Hz), 147.4, 142.5, 142.1, 136.4, 136.3, 136.0, 130.1 (^3^*J*_C–F_ = 8.1 Hz), 129.0, 128.7, 128.7, 127.6, 126.0, 123.6, 123.3 (^4^*J*_C–F_ = 3.3 Hz), 118.1 (t, ^1^*J*_C–F_ = 242.1 Hz), 115.5 (^2^*J*_C–F_ = 21.3 Hz), 81.3, 70.9, 62.4 (dd, ^2^*J*_C–F_ = 27.3, 21.6 Hz), 51.0 (d, ^3^*J*_C–F_ = 7.5 Hz) ppm. ^19^F NMR (376 MHz, CDCl_3_): *δ* −113.8, −116.9 (d, *J* = 289.1 Hz, 1F), −121.8 (d, *J* = 289.1 Hz, 1F) ppm. HRMS (ESI^+^): *m*/*z* calculated for C_26_H_17_F_3_NO_3_S [M + H]^+^ 480.0876, found 480.0879.

(2*S*,4′*S*,5′*S*)-4′-(4-Chlorophenyl)-5′-(difluoromethyl)-3*H*-dispiro[benzo[*b*]thiophene-2,2′-pyrrolidine-3′,2″-indene]-1″,3,3″-trione (**3ha**). From **1h** (26.8 mg, 0.10 mmol) and **2a** (22.7 mg, 0.10 mmol), compound **3ha** (49.0 mg, 98% yield) was obtained as a light-yellow solid, m.p. 123–127 °C. HPLC (Daicel Chiralpak IC column, mobile phase *n*-hexane/2-propanol = 85:15, flow rate 1.0 mL/min, detection wavelength 254 nm): *t*_R_ = 9.4 min (minor), *t*_R_ = 10.5 min (major); 24% ee. [α]_D_^25^ = −41.3 (*c* = 0.53, CH_2_Cl_2_). ^1^H NMR (400 MHz, CDCl_3_): *δ* 7.84–7.80 (m, 2H, ArH), 7.75–7.71 (m, 1H, ArH), 7.67 (d, *J* = 4.0 Hz, 2H, ArH), 7.43 (td, *J*_1_ = 7.6, *J*_2_ = 1.2 Hz, 1H, ArH), 7.21 (t, *J* = 7.5 Hz, 1H, ArH), 7.07–7.01 (m, 5H, ArH), 6.23 (td, *J*_1_ = 56.8 Hz, *J*_2_ = 6.8 Hz, 1H, CF_2_H), 4.80 (d, *J* = 9.6 Hz, 1H, CH), 4.66–4.58 (m, 1H, CH), 3.36 (br s, 1H, NH) ppm. ^13^C NMR (100 MHz, CDCl_3_): *δ* 200.2, 198.4, 195.9, 147.3, 142.4, 142.1, 136.4, 136.3, 136.1, 133.8, 131.5, 129.9, 128.9, 128.7, 127.6, 126.0, 123.5, 123.33, 123.27, 118.1 (t, ^1^*J*_C–F_ = 242.1 Hz), 81.4, 70.7, 62.3 (dd, ^2^*J*_C–F_ = 27.4, 21.6 Hz), 50.9 (d, ^3^*J*_C–F_ = 7.6 Hz) ppm. ^19^F NMR (376 MHz, CDCl_3_): *δ* −117.0 (d, *J* = 289.1 Hz, 1F), −121.8 (d, *J* = 289.5 Hz, 1F) ppm. HRMS (ESI^+^): *m*/*z* calculated for C_26_H_17_ClF_2_NO_3_S [M + H]^+^ 496.0581, found 496.0578.

(2*S*,4′*S*,5′*S*)-4′-(4-Bromophenyl)-5′-(difluoromethyl)-3*H*-dispiro[benzo[*b*]thiophene-2,2′-pyrrolidine-3′,2″-indene]-1″,3,3″-trione (**3ia**). From **1i** (31.2 mg, 0.10 mmol) and **2a** (22.7 mg, 0.10 mmol), compound **3ia** (47.4 mg, 88% yield) was obtained as a light-yellow solid, m.p. 185–187 °C. HPLC (Daicel Chiralpak ADH column, mobile phase *n*-hexane/2-propanol = 70:30, flow rate 1.0 mL/min, detection wavelength 254 nm): *t*_R_ = 19.4 min (major), *t*_R_ = 39.3 min (minor); 41% ee. [α]_D_^25^ = −103.8 (*c* = 0.67, CH_2_Cl_2_). ^1^H NMR (400 MHz, CDCl_3_): *δ* 7.83 (t, *J* = 8.2 Hz, 2H, ArH), 7.76–7. 72 (m, 1H, ArH), 7.67 (d, *J* = 3.6 Hz, 2H, ArH), 7.43 (t, *J* = 7.6 Hz, 1H, ArH), 7.24–7.18 (m, 3H, ArH), 7.03–6.98 (m, 3H, ArH), 6.23 (td, *J*_1_ = 56.8 Hz, *J*_2_ = 6.8 Hz, 1H, CF_2_H), 4.78 (d, *J* = 9.6 Hz, 1H, CH), 4.66–4.57 (m, 1H, CH), 3.35 (br s, 1H, NH) ppm. ^13^C NMR (100 MHz, CDCl_3_): *δ* 200.3, 198.6, 196.0, 163.4, 160.9, 147.4, 142.5, 142.1, 136.4, 136.3, 136.0, 130.2, 130.1, 129.0, 127.6, 126.0, 123.6, 123.3, 123.2, 122.1, 118.1 (t, ^1^*J*_C–F_ = 242.1 Hz), 115.6, 115.4, 81.3, 70.9, 62.4 (dd, ^2^*J*_C–F_ = 27.2, 21.6 Hz), 50.9 (d, ^3^*J*_C–F_ = 7.5 Hz) ppm. ^19^F NMR (376 MHz, CDCl_3_): *δ* −117.0 (d, *J* = 289.5 Hz, 1F), −121.8 (d, *J* = 289.5 Hz, 1F) ppm. HRMS (ESI^+^): *m*/*z* calculated for C_26_H_17_^79^BrF_2_NO_3_S [M + H]^+^ 540.0076, found 540.0062; calculated for C_26_H_17_^81^BrF_2_NO_3_S [M + H]^+^ 542.0055, found 542.0055.

(2*S*,4′*S*,5′*S*)-4′-(3-Bromophenyl)-5′-(difluoromethyl)-3*H*-dispiro[benzo[*b*]thiophene-2,2′-pyrrolidine-3′,2″-indene]-1″,3,3″-trione (**3ja**). From **1j** (31.2 mg, 0.10 mmol) and **2a** (22.7 mg, 0.10 mmol), compound **3ja** (45.0 mg, 84% yield) as a light-yellow solid, m.p. 207–209 °C. HPLC (Daicel Chiralpak ADH column, mobile phase *n*-hexane/2-propanol = 70:30, flow rate 1.0 mL/min, detection wavelength 254 nm): *t*_R_ = 15.2 min (major), *t*_R_ = 29.3 min (minor); 81% ee. [α]_D_^25^ = −47.6 (*c* = 0.58, CH_2_Cl_2_). ^1^H NMR (400 MHz, CDCl_3_): *δ* 7.83 (t, *J* = 6.4 Hz, 2H, ArH), 7.74–7.64 (m, 3H, ArH), 7.46–7.42 (m, 1H, ArH), 7.25 (s, 1H, ArH), 7.22 (t, *J* = 7.4 Hz, 1H, ArH), 7.15 (d, *J* = 8.0 Hz, 1H, ArH), 7.06 (d, *J* = 8.0 Hz, 1H, ArH), 7.03 (d, *J* = 8.0 Hz, 1H, ArH), 6.93 (t, *J* = 8.0 Hz, 1H, ArH), 6.24 (td, *J*_1_ = 56.8 Hz, *J*_2_ = 6.8 Hz, 1H, CF_2_H), 4.78 (d, *J* = 9.6 Hz, 1H, CH), 4.66–4.56 (m, 1H, CH), 3.37 (br s, 1H, NH) ppm. ^13^C NMR (100 MHz, CDCl_3_): *δ* 200.2, 198.3, 195.7, 147.4, 142.4, 142.0, 136.39, 136.35, 136.0, 135.3, 131.6, 131.2, 130.0, 129.0, 127.7, 127.2, 126.0, 123.5, 123.3, 122.5, 118.0 (t, ^1^*J*_C–F_ = 242.2 Hz), 81.2, 70.8, 62.2 (dd, ^2^*J*_C–F_ = 27.6, 21.8 Hz), 51.1 (d, ^3^*J*_C–F_ = 7.6 Hz) ppm. ^19^F NMR (376 MHz, CDCl_3_): *δ* −117.1 (d, *J* = 289.1 Hz, 1F), −121.7 (d, *J* = 289.1 Hz, 1F) ppm. HRMS (ESI^+^): *m*/*z* caluclated for C_26_H_17_^79^BrF_2_NO_3_S [M + H]^+^ 540.0076, found 540.0063; calculated for C_26_H_17_^81^BrF_2_NO_3_S [M + H]^+^ 542.0055, found 542.0048.

(2*S*,4′*S*,5′*S*)-4′-(3,5-Dichlorophenyl)-5′-(difluoromethyl)-3*H*-dispiro[benzo[*b*]thiophene-2,2′-pyrrolidine-3′,2″-indene]-1″,3,3″-trione (**3ka**). From **1k** (30.2 mg, 0.10 mmol) and **2a** (22.7 mg, 0.10 mmol), compound **3ka** (48.7 mg, 92% yield) was obtained as a light-yellow solid, m.p. 206–208 °C. HPLC (Daicel Chiralpak ADH column, mobile phase *n*-hexane/2-propanol = 70:30, flow rate 1.0 mL/min, detection wavelength 254 nm): *t*_R_ = 16.0 min (major), *t*_R_ = 32.7 min (minor); 14% ee. [α]_D_^25^ = −24.2 (*c* = 0.48, CH_2_Cl_2_). ^1^H NMR (400 MHz, CDCl_3_): *δ* 7.87 (d, *J* = 8.0 Hz, 1H, ArH), 7.81 (dd, *J*_1_ = 8.0 Hz, *J*_2_ = 1.2 Hz, 1H, ArH), 7.75–7.69 (m, 3H, ArH), 7.45 (td, *J* = 7.6, *J*_2_ = 1.2 Hz, 1H, ArH), 7.30 (d, *J* = 8.4 Hz, 1H, ArH), 7.25–7.21 (m, 1H, ArH), 7.11 (d, *J* = 2.0 Hz, 1H, ArH), 7.05 (dd, *J* = 8.6, 2.2 Hz, 2H, ArH), 6.29 (td, *J*_1_ = 56.8 Hz, *J*_2_ = 7.0 Hz, 1H, CF_2_H), 5.59 (d, *J* = 9.6 Hz, 1H, CH), 4.49–4.41 (m, 1H, CH), 3.29 (br s, 1H, NH) ppm. ^13^C NMR (100 MHz, CDCl_3_): *δ* 200.6, 198.7, 194.7, 147.4, 142.4, 141.8, 136.30, 136.28, 135.8, 134.1, 130.4, 129.8, 129.7, 129.1, 127.7, 127.1, 125.9, 123.52, 123.48, 123.2, 117.9 (t, ^1^*J*_C–F_ = 242.2 Hz), 81.0, 71.0, 64.1 (dd, ^2^*J*_C–F_ = 27.2, 21.4 Hz), 46.5 (d, ^3^*J*_C–F_ = 7.8 Hz) ppm. ^19^F NMR (376 MHz, CDCl_3_): *δ* −116.8 (d, *J* = 289.9 Hz, 1F), −122.6 (d, *J* = 289.9 Hz, 1F) ppm. HRMS (ESI^+^): *m*/*z* calculated for C_26_H_16_Cl_2_F_2_NO_3_S [M + H]^+^ 530.0191, found 530.0191.

(2*S*,4′*S*,5′*S*)-5′-(Difluoromethyl)-4′-(4-nitrophenyl)-3*H*-dispiro[benzo[*b*]thiophene-2,2′-pyrrolidine-3′,2″-indene]-1″,3,3″-trione (**3la**). From **1l** (27.9 mg, 0.10 mmol) and **2a** (22.7 mg, 0.10 mmol), compound **3la** (45.6 mg, 90% yield) was obtained as a light yellow solid, m.p. 224–226 °C. HPLC (Daicel Chiralpak IC column, mobile phase *n*-hexane/2-propanol = 80:20, flow rate 1.0 mL/min, detection wavelength 254 nm): *t*_R_ = 21.0 min (minor), *t*_R_ = 27.4 min (major); 61% ee. [α]_D_^25^ = −1.01 (*c* = 1.38, CH_2_Cl_2_). ^1^H NMR (700 MHz, CDCl_3_): *δ* 7.95 (d, *J* = 9.1 Hz, 2H, ArH), 7.85–7.82 (m, 2H, ArH), 7.76–7.73 (m, 1H, ArH), 7.68 (d, *J* = 4.2 Hz, 2H, ArH), 7.45 (d, *J* = 7.4 Hz, 2H, ArH), 7.33 (d, *J* = 9.1 Hz, 2H, ArH), 7.23 (t, *J* = 7.7 Hz, 1H, ArH), 7.03 (d, *J* = 8.4 Hz, 1H, ArH), 6.26 (td, *J*_1_ = 56.7, *J*_2_ = 7.0 Hz, 1H, CF_2_H), 4.94 (d, *J* = 9.8 Hz, 1H, CH), 4.73–4.69 (m, 1H, CH), 3.41 (d, *J* = 7.7 Hz, 1H, NH) ppm. ^13^C NMR (100 MHz, CDCl_3_): *δ* 199.9, 197.9, 195.4, 147.4, 147.2, 142.6, 142.3, 141.9, 140.7, 136.7, 136.5, 136.4, 134.2, 129.6, 128.8, 127.7, 126.1, 124.3, 123.64, 123.60, 123.4, 117.9 (t, ^1^*J*_C–F_ = 242.2 Hz), 81.6, 70.4, 62.4 (dd, ^2^*J*_C–F_ = 27.9, 22.1 Hz), 50.8 (d, ^3^*J*_C–F_ = 7.7 Hz) ppm. ^19^F NMR (376 MHz, CDCl_3_): *δ* −116.9 (d, *J* = 290.5 Hz, 1F), −121.7 (d, *J* = 290.4 Hz, 1F) ppm. HRMS (ESI^+^): *m*/*z* calculated for C_26_H_17_F_2_N_2_O_5_S [M + H]^+^ 507.0821, found 507.0810.

(2*S*,4′*S*,5′*S*)-5′-(Difluoromethyl)-4′-(thiophen-2-yl)-3*H*-dispiro[benzo[*b*]thiophene-2,2′-pyrrolidine-3′,2″-indene]-1″,3,3″-trione (**3ma**). From **1m** (24.0 mg, 0.10 mmol) and **2a** (22.7 mg, 0.10 mmol), compound **3ma** (41.1 mg, 88% yield) was obtained as a light-yellow solid, m.p. 208–210 °C. HPLC (Daicel Chiralpak IC column, mobile phase *n*-hexane/2-propanol = 80:20, flow rate 1.0 mL/min, detection wavelength 254 nm): *t*_R_ = 12.4 min (minor), *t*_R_ = 16.2 min (major); 81% ee. [α]_D_^25^ = −46.1 (*c* = 0.63, CH_2_Cl_2_). ^1^H NMR (400 MHz, CDCl_3_): *δ* 7.88 (d, *J* = 7.6 Hz, 1H, ArH), 7.82 (d, *J* = 7.6 Hz, 1H, ArH), 7.76–7.66 (m, 3H, ArH), 7.46–7.41 (m, 1H, ArH), 7.21 (t, *J* = 7.4 Hz, 1H, ArH), 7.03 (d, *J* = 7.6 Hz, 1H, ArH), 6.93 (d, *J* = 4.8 Hz, 1H, ArH), 6.80 (d, *J* = 3.4 Hz, 1H, ArH), 6.68 (dd, *J*_1_ = 5.0 Hz, *J*_2_ = 3.8 Hz, 1H, ArH), 6.24 (td, *J*_1_ = 56.6 Hz, *J*_2_ = 6.8 Hz, 1H, CF_2_H), 5.11 (d, *J* = 9.6 Hz, 1H, CH), 4.60–4.51 (m, 1H, CH), 3.37 (s, 1H, NH) ppm. ^13^C NMR (100 MHz, CDCl_3_): *δ* 200.3, 198.6, 195.7, 147.5, 142.8, 142.3, 136.4, 136.2, 135.9, 135.3, 128.9, 127.7, 127.1, 126.7, 126.0, 125.1, 123.6, 123.4, 123.3, 117.7 (t, ^1^*J*_C–F_ = 242.3 Hz), 81.0, 70.6, 64.4 (dd, ^2^*J*_C–F_ = 26.9, 21.6 Hz), 47.0 (d, ^3^*J*_C–F_ = 7.6 Hz) ppm. ^19^F NMR (376 MHz, CDCl_3_): *δ* −117.5 (d, *J* = 289.1 Hz, 1F), −121.9 (d, *J* = 289.1 Hz, 1F) ppm. HRMS (ESI^+^): *m*/*z* calculated for C_24_H_16_F_2_NO_3_S_2_ [M + H]^+^ 468.0535, found 468.0526.

(2*S*,4′*S*,5′*S*)-5′-(Difluoromethyl)-4′-(furan-2-yl)-3*H*-dispiro[benzo[*b*]thiophene-2,2′-pyrrolidine-3′,2″-indene]-1″,3,3″-trione (**3na**). From **1n** (22.4 mg, 0.10 mmol) and **2a** (22.7 mg, 0.10 mmol), compound **3na** was obtained as a light-yellow solid, m.p. 216–218 °C. HPLC (Daicel Chiralpak ADH column, mobile phase *n*-hexane/2-propanol = 70:30, flow rate 1.0 mL/min, detection wavelength 254 nm): *t*_R =_ 14.0 min (major), *t*_R_ = 41.9 min (minor); 11% ee. [α]_D_^25^ = −36.1 (*c* = 0.33, CH_2_Cl_2_). ^1^H NMR (400 MHz, CDCl_3_): *δ* 7.90–7.86 (m, 1H, ArH), 7.83–7.70 (m, 5H, ArH), 7.46–7.42 (m, 1H, ArH), 7.24–7.19 (m, 1H, ArH), 7.04 (d, *J* = 8.0 Hz, 1H, ArH), 6.93 (dd, *J*_1_ = 1.6, *J*_2_ = 0.8 Hz, 1H, ArH), 6.26 (dd, *J*_1_ = 56.8 Hz, *J*_2_ = 7.2 Hz, 1H, CF_2_H), 6.10 (d, *J* = 3.2 Hz, 1H, ArH), 6.03 (dd, *J*_1_ = 3.2 Hz, *J*_2_ = 2.0 Hz, 1H, ArH), 4.91 (d, *J* = 9.6 Hz, 1H, CH), 4.53–4.45 (m, 1H. CH), 3.39 (br s, 1H, NH) ppm. ^13^C NMR (100 MHz, CDCl_3_): *δ* 200.3, 197.9, 195.4, 149.0, 147.6, 142.3, 136.3, 136.1, 135.8, 134.9, 129.3, 127.7, 125.9, 123.6, 123.4, 123.3, 122.9, 117.6 (t, ^1^*J*_C–F_ = 241.9 Hz), 110.3, 108.9, 80.5, 69.3, 62.1 (dd, ^2^*J*_C–F_ = 27.8, 22.2 Hz), 45.5 (d, ^3^*J*_C–F_ = 7.9 Hz) ppm. ^19^F NMR (376 MHz, CDCl_3_): *δ* −117.5 (d, *J* = 288.8 Hz, 1F), −122.0 (d, *J* = 288.8 Hz, 1F) ppm. HRMS (ESI^+^): *m*/*z* calculated for C_24_H_16_F_2_NO_4_S [M + H]^+^ 452.0763, found 452.0767.

(2*S*,4′*S*,5′*S*)-5′-(Difluoromethyl)-4′-(pyridin-2-yl)-3*H*-dispiro[benzo[*b*]thiophene-2,2′-pyrrolidine-3′,2″-indene]-1″,3,3″-trione (**3oa**). From **1o** (23.5 mg, 0.10 mmol) and **2a** (22.7 mg, 0.10 mmol), compound **3oa** (43.9 mg, 95% yield) was obtained as a white solid, m.p. 207–210 °C. HPLC (Daicel Chiralpak ADH column, mobile phase *n*-hexane/2-propanol = 60:40, flow rate 1.0 mL/min, detection wavelength 254 nm): *t*_R_ = 19.2 min (major), *t*_R_ = 22.4 min (minor); 13% ee. [α]_D_^25^ = +13.0 (*c* = 0.71, CH_2_Cl_2_). ^1^H NMR (400 MHz, DMSO-d_6_): *δ* 8.27 (dd, *J*_1_ = 4.6 Hz, *J*_2_ = 1.0 Hz, 1H, ArH), 8.21 (d, *J* = 2.0 Hz, 1H, ArH), 7.90 (d, *J* = 7.6 Hz, 2H, ArH), 7.84–7.80 (m, 1H, ArH), 7.72 (d, *J* = 8.4 Hz, 2H, ArH), 7.58–7.53 (m, 2H, ArH), 7.32–7.25 (m, 2H, ArH), 7.18 (dd, *J*_1_ = 8.0 Hz, *J*_2_ = 4.8 Hz, 1H, ArH), 6.24 (td, *J*_1_ = 56.8 Hz, *J*_2_ = 5.8 Hz, 1H, CF_2_H), 5.15 (d, *J* = 5.6 Hz, 1H, CH), 4.69–4.50 (m, 2H, CH + NH) ppm. ^13^C NMR (100 MHz, DMSO-d_6_): *δ* 200.2, 196.9, 195.8, 149.2, 149.1, 146.9, 141.5, 141.2, 137.6, 137.0, 136.7, 135.8, 129.1, 128.3, 126.8, 126.0, 123.9, 123.4, 123.2, 123.1, 118.2 (t, ^1^*J*_C–F_ = 240.2 Hz), 82.7, 70.0, 60.9 (dd, ^2^*J*_C–F_ = 26.8, 20.3 Hz), 54.8 ppm. ^19^F NMR (376 MHz, DMSO-d_6_): *δ* −115.6 (d, *J* = 285.8 Hz, 1F), −120.7 (d, *J* = 285.8 Hz, 1F) ppm. HRMS (ESI^+^): *m*/*z* calculated for C_25_H_17_F_2_N_2_O_3_S [M + H]^+^ 463.0923, found 463.0917.

(2*S*,4′*S*,5′*S*)-5′-(Difluoromethyl)-7-methyl-4′-phenyl-3*H*-dispiro[benzo[*b*]thiophene-2,2′-pyrrolidine-3′,2″-indene]-1″,3,3″-trione (**3ab**). From **1a** (23.4 mg, 0.10 mmol) and **2b** (24.1 mg, 0.10 mmol), compound **3ab** (43.2 mg, 91% yield) was obtained as a white solid, m.p. 189-191 °C. HPLC (Daicel Chiralpak IC column, mobile phase *n*-hexane/2-propanol = 90:10, flow rate 1.0 mL/min, detection wavelength 254 nm): *t*_R_ = 17.0 min (minor), *t*_R_ = 24.4 min (major); 49% ee. [α]_D_^25^ = −155.8 (*c* = 1.18, CH_2_Cl_2_). ^1^H NMR (400 MHz, CDCl_3_): *δ* 7.78 (d, *J* = 7.6 Hz, 1H, ArH), 7.69–7.58 (m, 4H, ArH), 7.24 (s, 1H, ArH), 7.15 (d, *J* = 7.6 Hz, 1H, ArH), 7.12–7.08 (m, 2H, ArH), 7.06–6.97 (m, 3H, ArH), 6.25 (td, *J*_1_ = 56.8, *J*_2_ = 6.8 Hz, 1H, CF_2_H), 4.85 (d, *J* = 10.0 Hz, 1H, CH), 4.72–4.64 (m, 1H, CH), 3.39 (s, 1H, NH), 2.02 (s, 3H, CH_3_) ppm. ^13^C NMR (100 MHz, CDCl_3_): *δ* 200.8, 198.8, 196.1, 147.1, 142.5, 142.1, 136.5, 136.0, 135.7, 132.8, 132.7, 128.9, 128.44, 128.41, 127.9, 126.0, 125.0, 123.2, 123.1, 118.1 (t, ^1^*J*_C–F_ = 241.9 Hz), 81.2, 71.2, 62.2 (dd, ^2^*J*_C–F_ = 27.2, 21.3 Hz), 52.0 (d, ^3^*J*_C–F_ = 7.5 Hz), 18.4 ppm. ^19^F NMR (376 MHz, CDCl_3_): *δ* −117.1 (d, *J* = 288.0 Hz, 1F), −121.8 (d, *J* = 288.4 Hz, 1F) ppm. HRMS (ESI^+^): *m*/*z* calculated for C_27_H_20_F_2_NO_3_S [M + H]^+^ 476.1127, found 476.1125.

### 3.4. Procedure for the Scaled-Up Synthesis of Compound ***3aa***

In a dried bottle, **1a** (0.468 g, 2.0 mmol), **2a** (0.454 g, 2.0 mmol), chiral organocatalyst **C9** (49.0 mg, 0.2 mmol, 20 mol%), and DCM (15.0 mL) were added. The mixture was stirred at room temperature for three days. After completion of the reaction, the residue was purified by flash column chromatography on silica gel to obtain the pure product **3aa** (0.83 g, 90% yield, >20:1 dr, 91% ee).

## 4. Conclusions

In summary, we have successfully developed an asymmetric [3+2] cycloaddition strategy for *N*-2,2-difluoroethyl benzothiophenone imines and 2-arylidene-1,3-indanediones. Under mild conditions, a series of dispiro[benzothiophenone-indandione-pyrrolidine] compounds were obtained in 84–98% yields with 3–93% ee and 9:1–>20:1 dr. The scalability of this strategy for large-scale asymmetric synthesis of such dispiro[benzothiophenone- indanedione-pyrrolidine] derivatives was demonstrated through scale-up experiments, providing strong support for future practical applications and drug development.

## Figures and Tables

**Table 1:** Optimization of the reaction conditions ^a^.

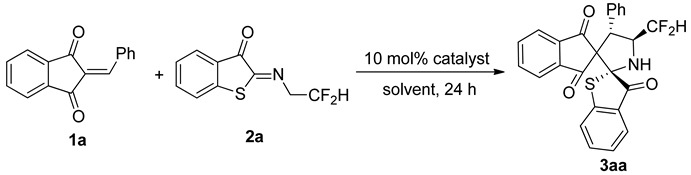						
**Entry**	**Solvent**	**Catalyst**	**Temperature** **(°C)**	**Yield ^b^ (%)**	**dr ^c^**	**ee ^d^ (%)**
1	CH_2_Cl_2_	**C1**	rt	80	>20:1	76
2	CH_2_Cl_2_	**C2**	rt	75	>20:1	76
3	CH_2_Cl_2_	**C3**	rt	92	>20:1	62
4	CH_2_Cl_2_	**C4**	rt	68	>20:1	54
5	CH_2_Cl_2_	**C5**	rt	86	>20:1	45
6	CH_2_Cl_2_	**C6**	rt	trace	–	–
7	CH_2_Cl_2_	**C7**	rt	82	>20:1	39
8	CH_2_Cl_2_	**C8**	rt	90	>20:1	50
9	CH_2_Cl_2_	**C9**	rt	95	>20:1	92
10	CH_2_Cl_2_	**C10**	rt	87	>20:1	81
11	CH_2_Cl_2_	**C11**	rt	83	>20:1	60
12	CHCl_3_	**C9**	rt	85	>20:1	41
13	DCE	**C9**	rt	86	>20:1	72
14	toluene	**C9**	rt	88	>20:1	69
15	MeCN	**C9**	rt	89	>20:1	57
16	THF	**C9**	rt	87	>20:1	43
17	MTBE	**C9**	rt	75	>20:1	27
18	dioxane	**C9**	rt	89	>20:1	61
19	Et_2_O	**C9**	rt	91	>20:1	33
20 ^e^	CH_2_Cl_2_	**C9**	rt	92	>20:1	88
21 ^f^	CH_2_Cl_2_	**C9**	rt	94	>20:1	82
22	CH_2_Cl_2_	**C9**	−10	84	>20:1	92
23	CH_2_Cl_2_	**C9**	40	90	>20:1	87
24 ^g^	CH_2_Cl_2_	**C9**	rt	N.R.	–	–

^a^ Unless otherwise specified, the reactions were carried out with **1a** (0.10 mmol), **2a** (0.10 mmol), and catalyst (10 mol%) in solvent (2.0 mL) for 24 h. ^b^ Product yield was determined after purification on column chromatography. ^c^ Determined by ^1^H NMR analysis. ^d^ The enantiomeric excess (ee) was determined by HPLC analysis. ^e^ 15 mol% catalyst was used. ^f^ 5 mol% catalyst was used. ^g^ 10 mol% benzoic acid was added.

## Data Availability

Data are contained within this article and Supplementary Materials.

## Supplementary Materials

The following supplementary materials can be downloaded at: https://www.mdpi.com/article/10.3390/molecules29204856/s1, spectroscopic data (^1^H and ^13^C NMR), chiral HPLC chromatograms for all new compounds **3**, and X-ray crystal data for compound **3aa**.
