# Supplementary material for: Organocatalyzed Enantioselective [3+2] Cycloaddition Reactions for Synthesis of Dispiro[benzothiophenone-indandione-pyrrolidine] Derivatives
Source: Molecules. 2024 Oct 13;29(20):4856. doi: 10.3390/molecules29204856 (PMC11510190; doi:10.3390/molecules29204856)
Supplement: Supplementary file 1 [file molecules-29-04856-s001.zip › molecules-3240253-supplementary.pdf]

Organocatalyzed Enantioselective [3+2] Cycloaddition Reactions for Synthesis of Dispiro[benzothiophenone-indandione-pyrrolidine] Derivatives

Hong-Yan Liu, Da-Ming Du*

School of Chemistry and Chemical Engineering, Beijing Institute of Technology, Beijing 100081, China; Key Laboratory of Medicinal Molecule Science and Pharmaceutical Technology, Ministry of Industry and Information Technology, Beijing 100081, China

E-mail: dudm@bit.edu.cn

Supporting Information

Contents

1. Copies of ^{1}H , ^{13}C NMR and ^{19}F NMR spectra of new compounds……………......................S1
2. Methodology for determining the dr…………………………………………………………..S25
3. X-ray single-crystal data for product **3aa**…………………..…..……………………. ……S26
4. Copies of HPLC chromatograms……………………..…..…………………………………...S27

1. Copies of ^{1}H, ^{13}C NMR and ^{19}F NMR spectra of new compounds

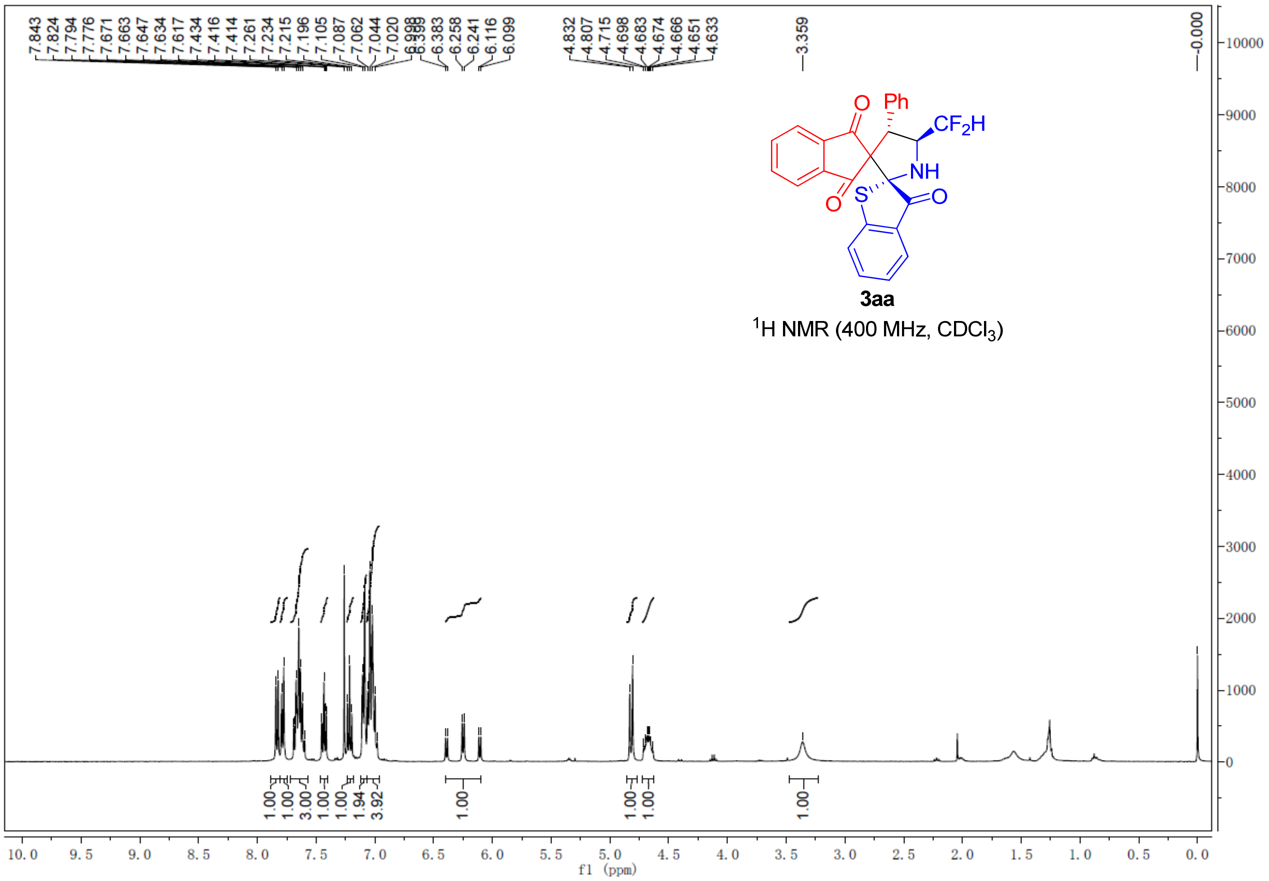

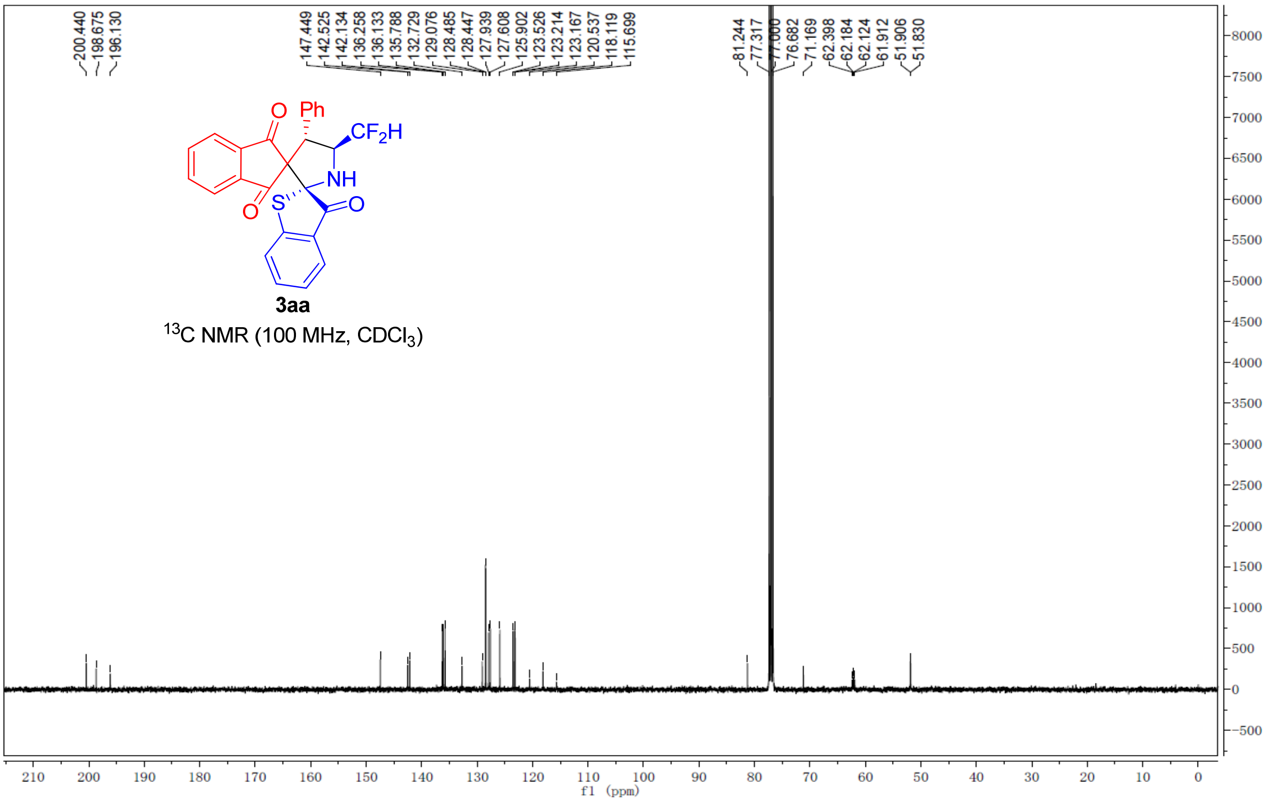

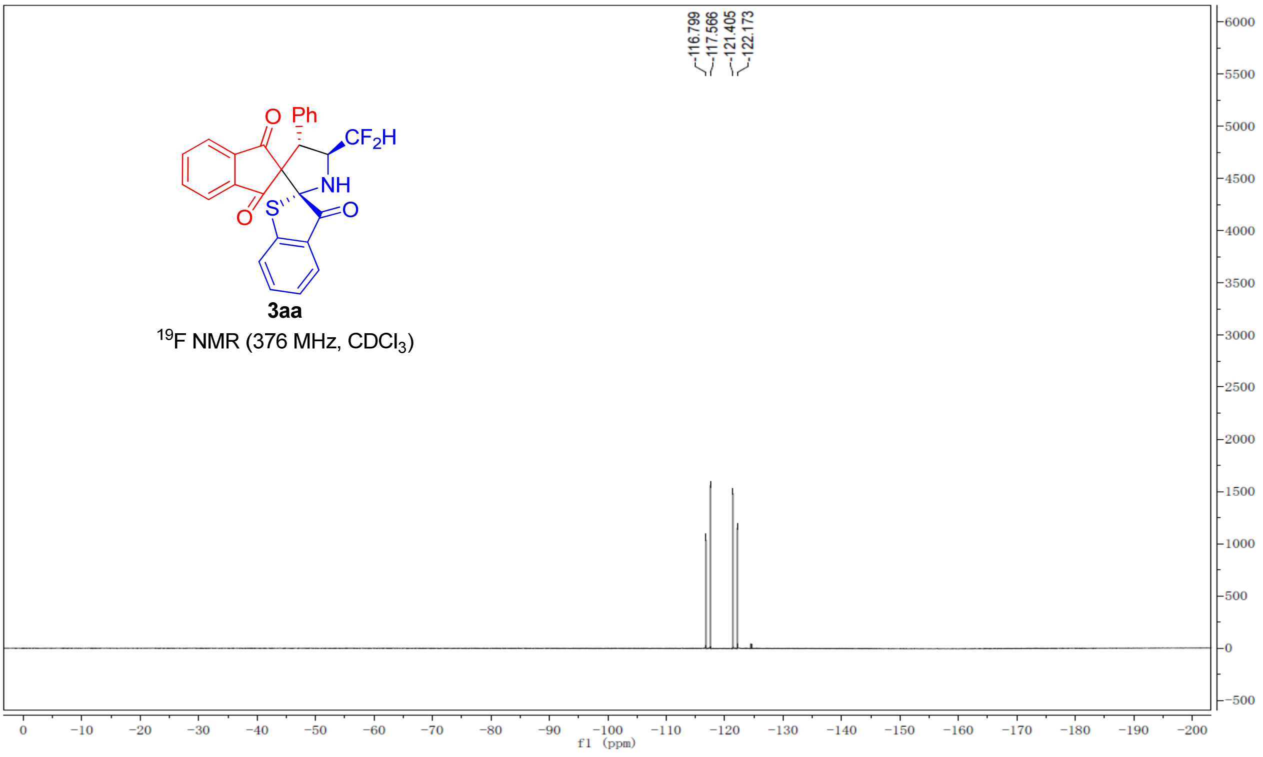

-116.799
-117.566
-121.405
-122.173
Ph
CF2H
NH
S
3aa
19F NMR (376 MHz, CDCl3)
f1 (ppm)

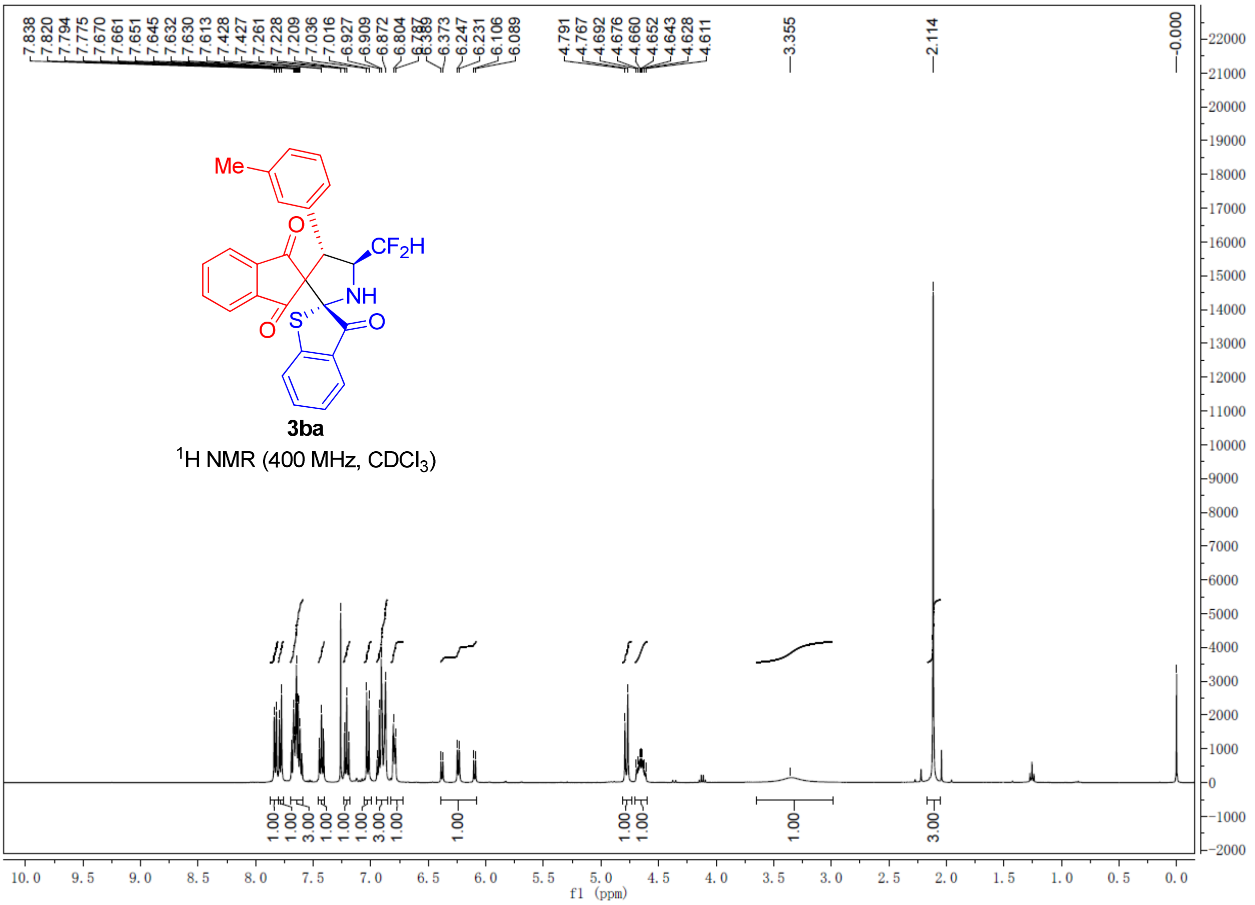

-3.355
-2.114
-0.000
Me
CF2H
NH
S
3ba
1H NMR (400 MHz, CDCl3)
f1 (ppm)

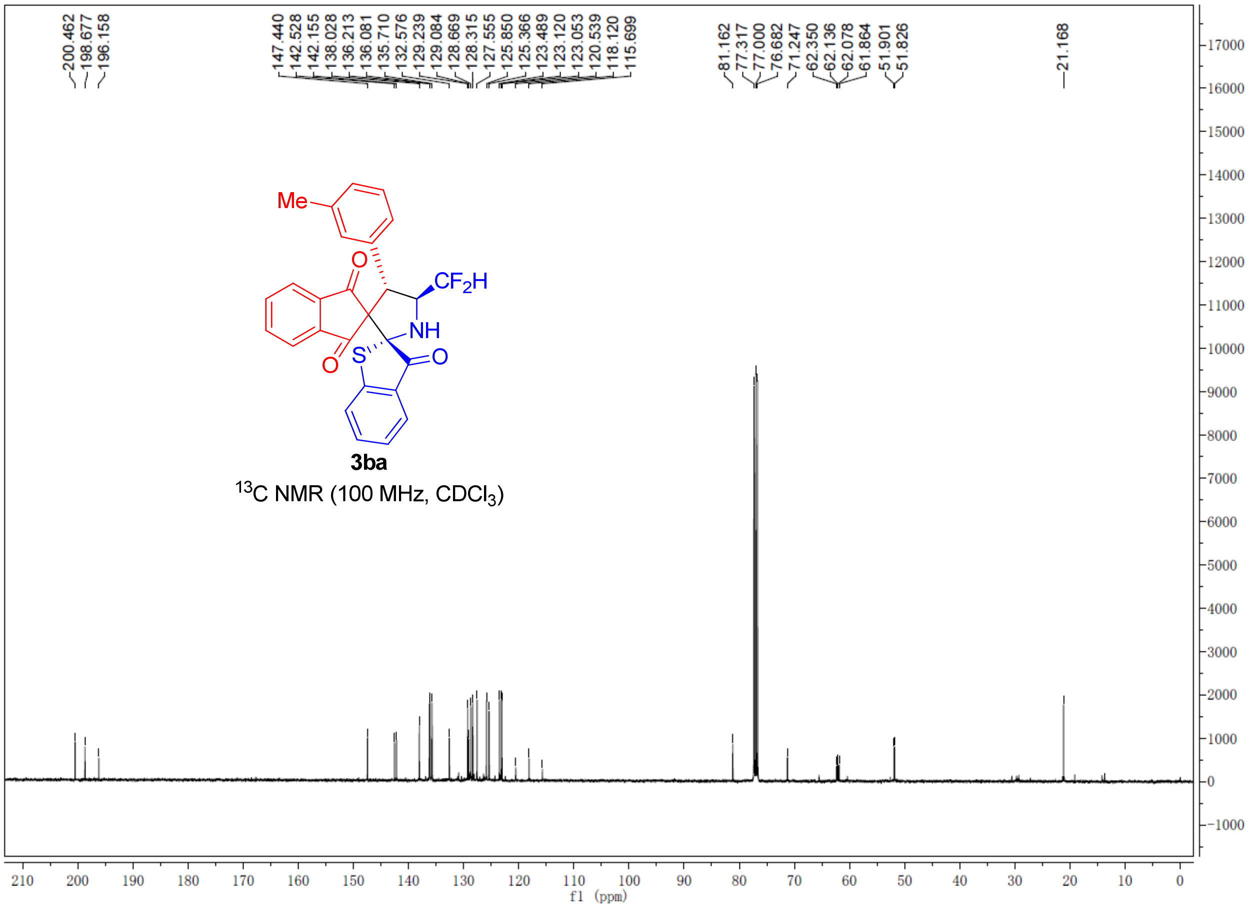
200.462
198.677
196.158
147.440
142.528
142.155
138.028
136.213
136.081
135.710
132.576
129.239
129.084
128.669
128.315
127.555
125.850
125.366
123.489
123.120
123.053
120.539
118.120
115.699
81.162
77.317
77.000
76.682
71.247
62.350
62.136
62.078
61.864
51.901
51.826
21.168
Me
O
CF2H
NH
S
O
O
3ba
13C NMR (100 MHz, CDCl3)
f1 (ppm)

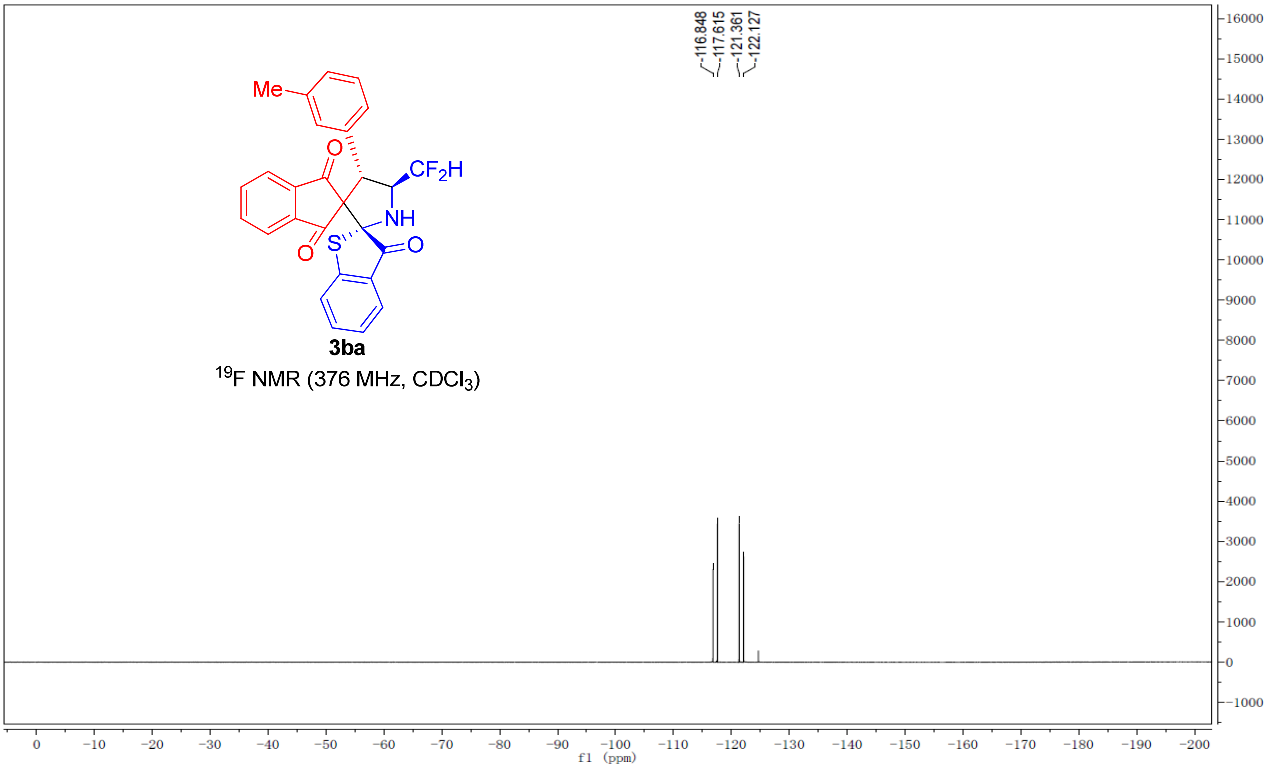
-116.848
-117.615
-121.361
-122.127
Me
O
CF2H
NH
S
O
O
3ba
19F NMR (376 MHz, CDCl3)
f1 (ppm)

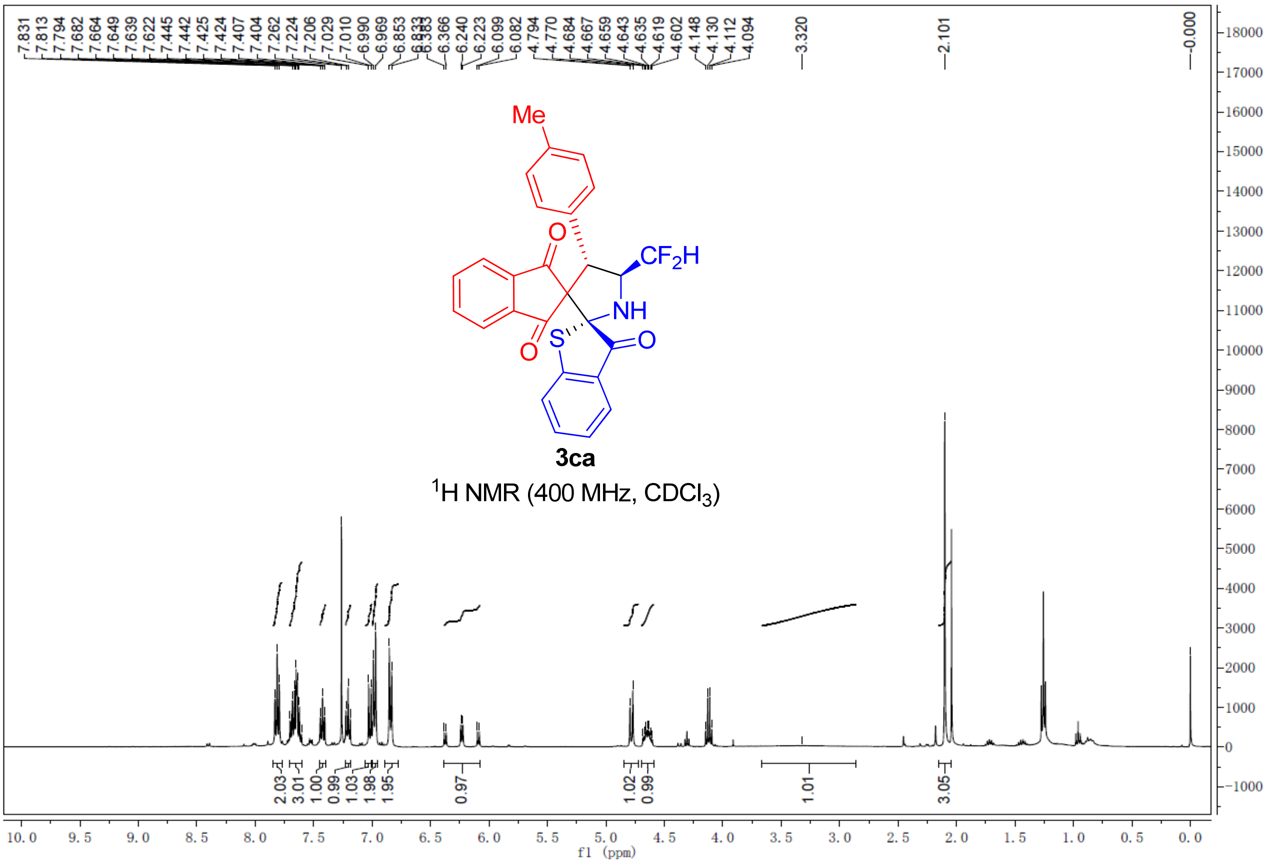
7.831 7.813 7.794 7.682 7.664 7.649 7.639 7.622 7.445 7.442 7.425 7.424 7.407 7.404 7.262 7.224 7.206 7.029 7.010 6.990 6.969 6.853 6.366 6.240 6.223 6.099 6.082 4.794 4.770 4.684 4.667 4.659 4.643 4.635 4.619 4.602 4.148 4.130 4.112 4.094
3.320
2.101
0.000
Me
O
CF2H
NH
S
O
O
3ca
1H NMR (400 MHz, CDCl3)
2.03 3.01 1.00 0.99 1.03 1.98 1.95 0.97 1.02 0.99 1.01 3.05
f1 (ppm)

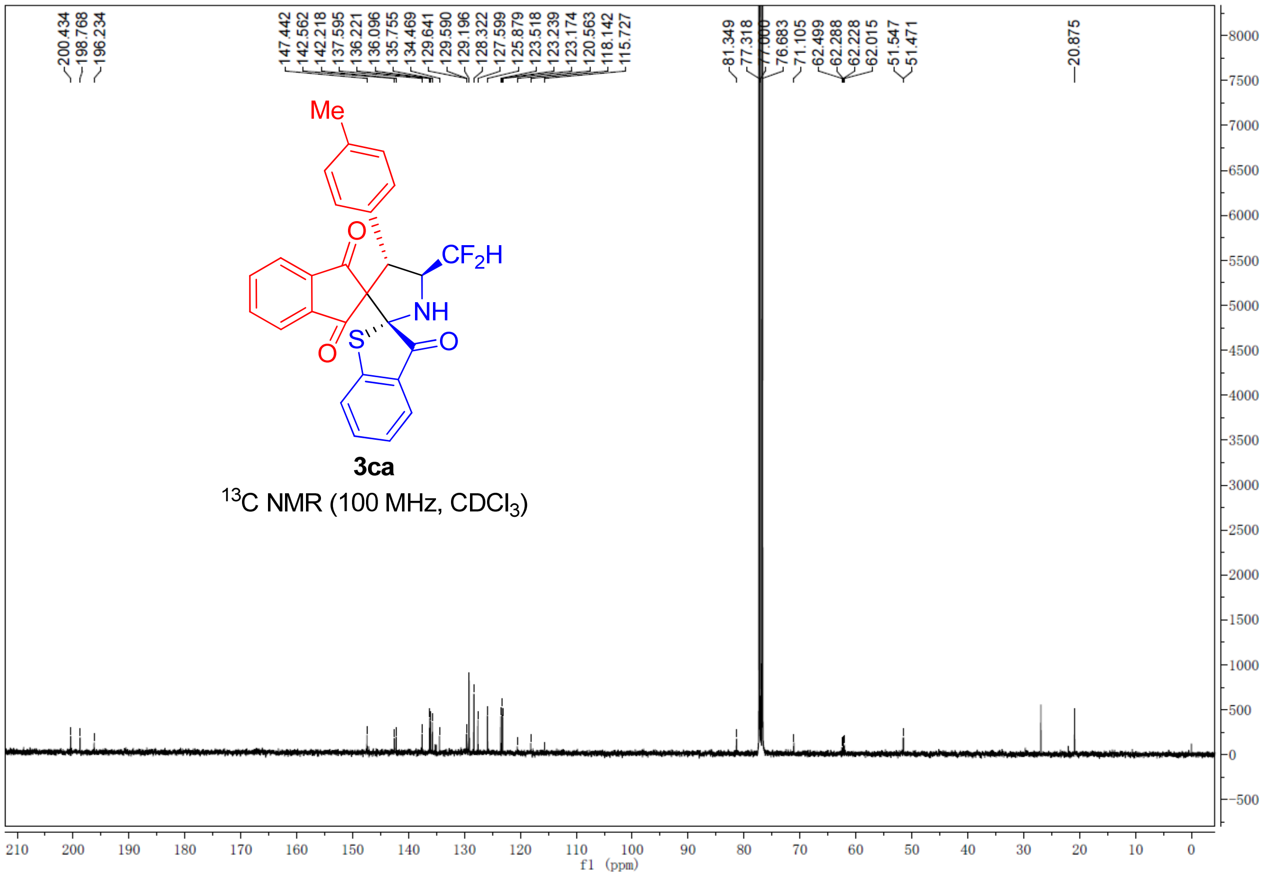
200.434 198.768 196.234
147.442 142.562 142.218 137.595 136.221 136.096 135.755 134.469 129.641 129.590 129.196 128.322 127.599 125.879 123.518 123.239 123.174 120.563 118.142 115.727
81.349 77.318 76.683 71.105 62.499 62.288 62.228 62.015 51.547 51.471
20.875
Me
O
CF2H
NH
S
O
O
3ca
13C NMR (100 MHz, CDCl3)
f1 (ppm)

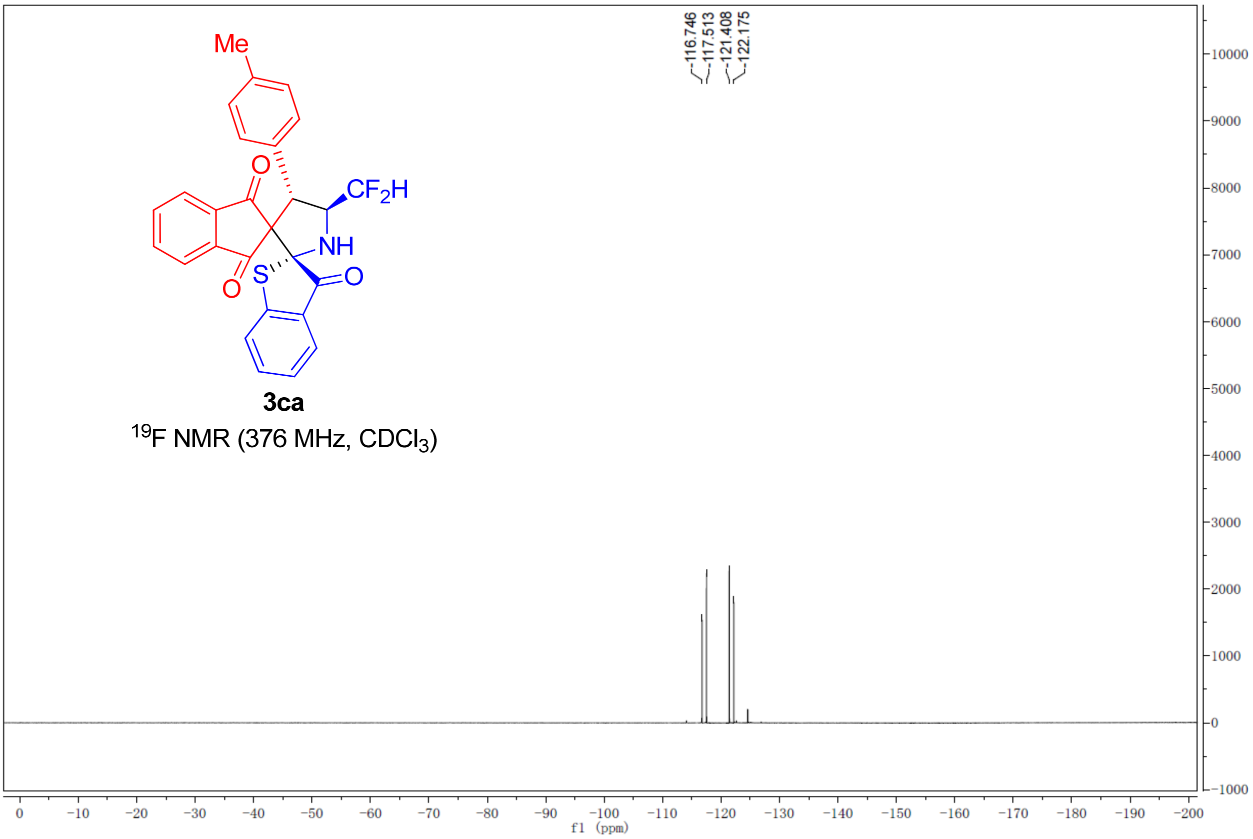

-116.746
-117.513
-121.408
-122.175
Me
O
CF2H
NH
S
O
O
3ca
19F NMR (376 MHz, CDCl3)
f1 (ppm)

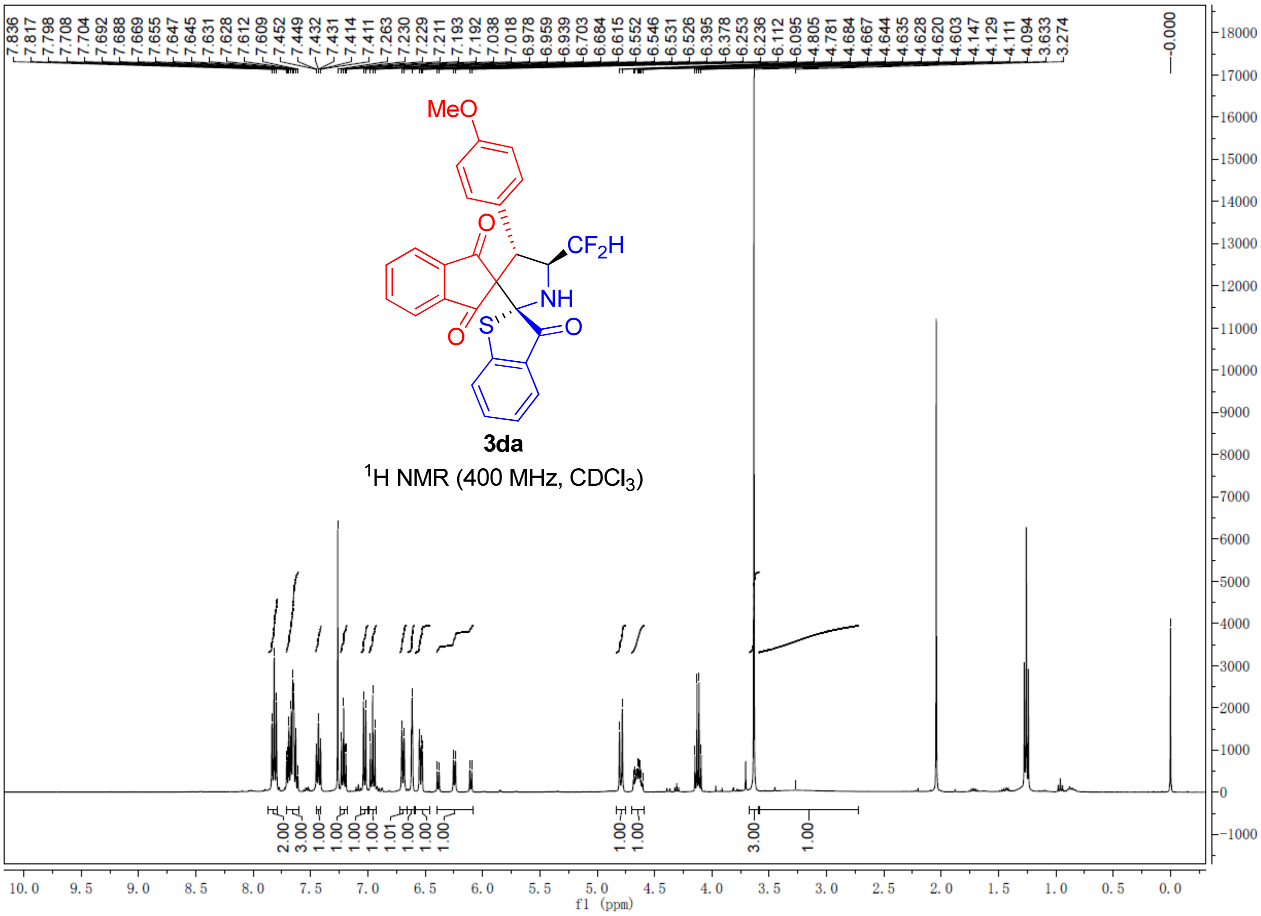

7.836
7.817
7.798
7.708
7.704
7.692
7.688
7.669
7.655
7.647
7.645
7.631
7.628
7.612
7.609
7.452
7.449
7.432
7.431
7.414
7.411
7.263
7.230
7.229
7.211
7.193
7.192
7.038
7.018
6.978
6.959
6.939
6.703
6.684
6.615
6.552
6.546
6.531
6.526
6.395
6.378
6.253
6.236
6.112
6.095
4.805
4.781
4.684
4.667
4.644
4.635
4.628
4.620
4.603
4.147
4.129
4.111
4.094
3.633
3.274
-0.000
MeO
O
CF2H
NH
S
O
O
3da
1H NMR (400 MHz, CDCl3)
f1 (ppm)

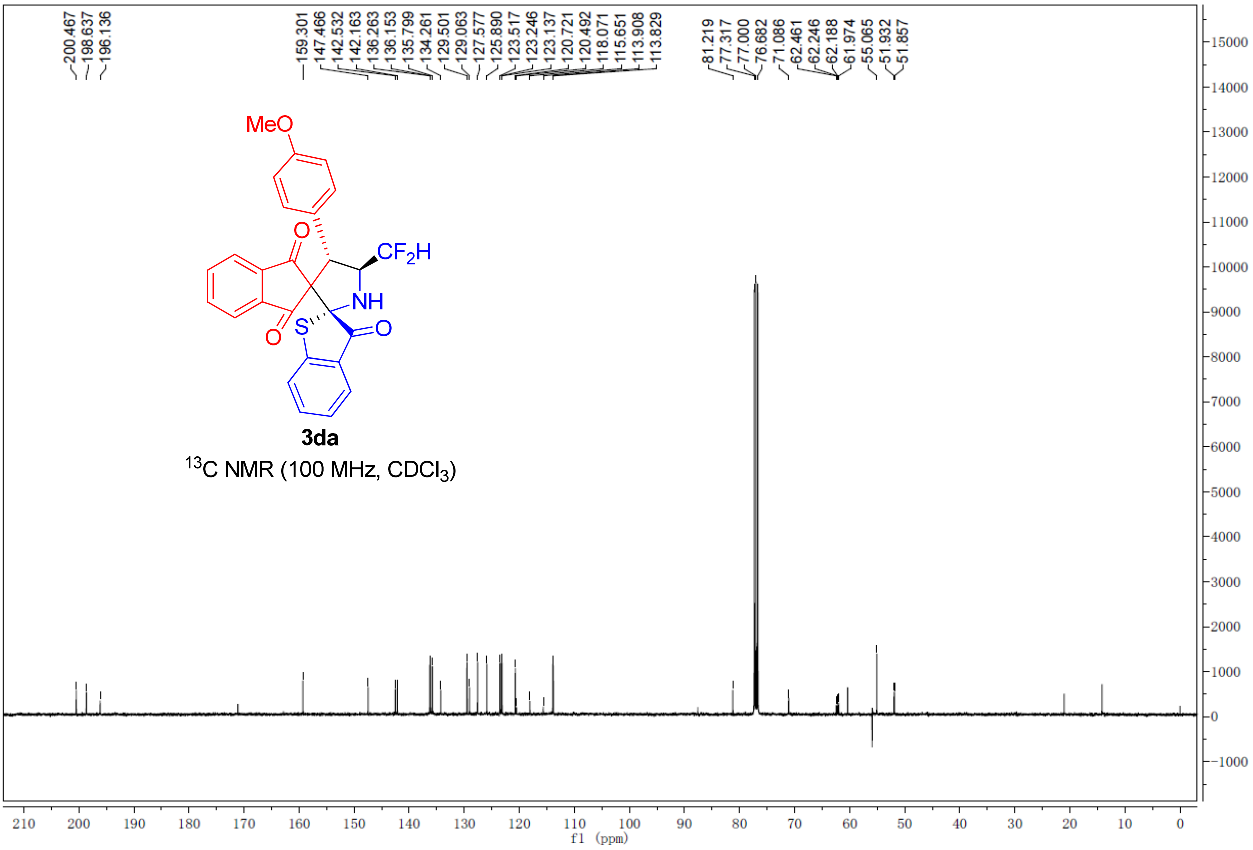

3da

^{13}C NMR (100 MHz, $CDCl_3$)

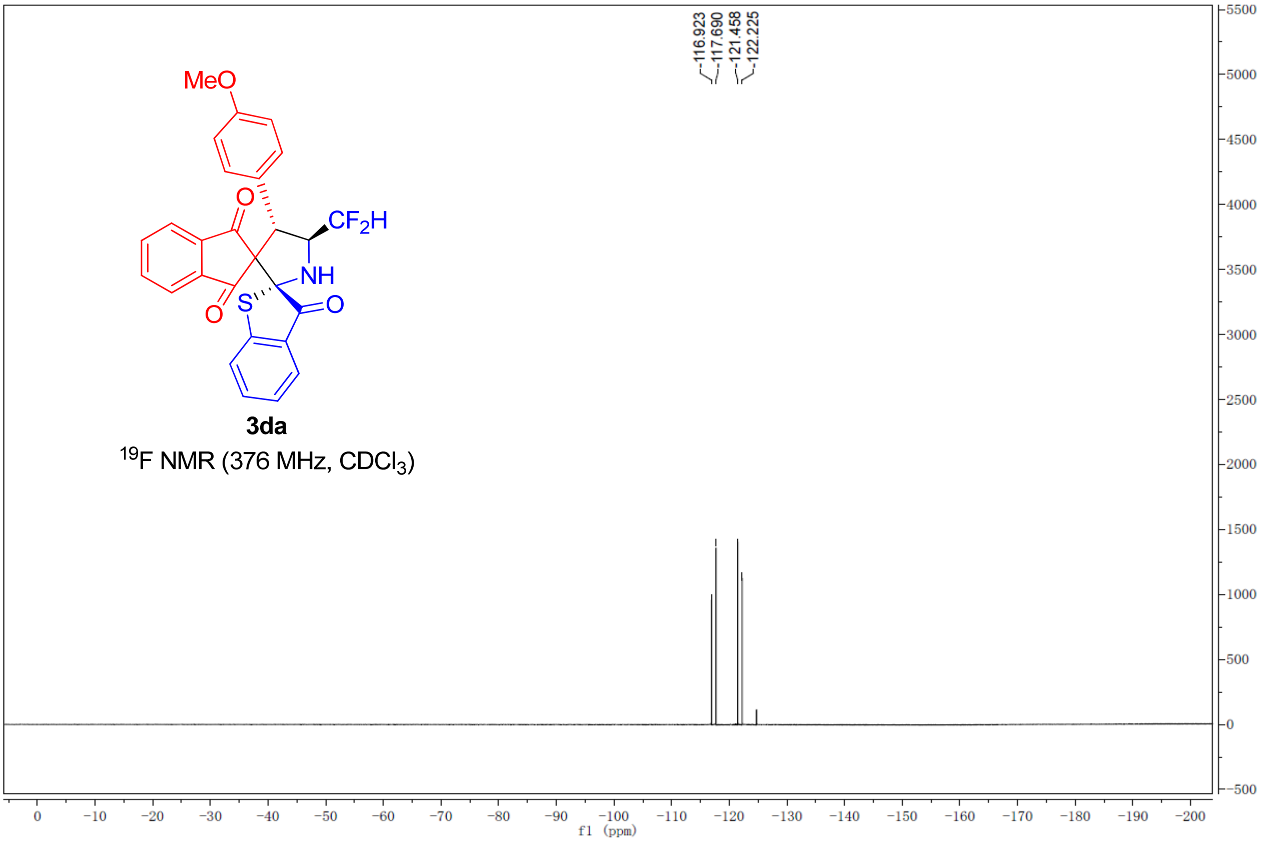

3da

^{19}F NMR (376 MHz, $CDCl_3$)

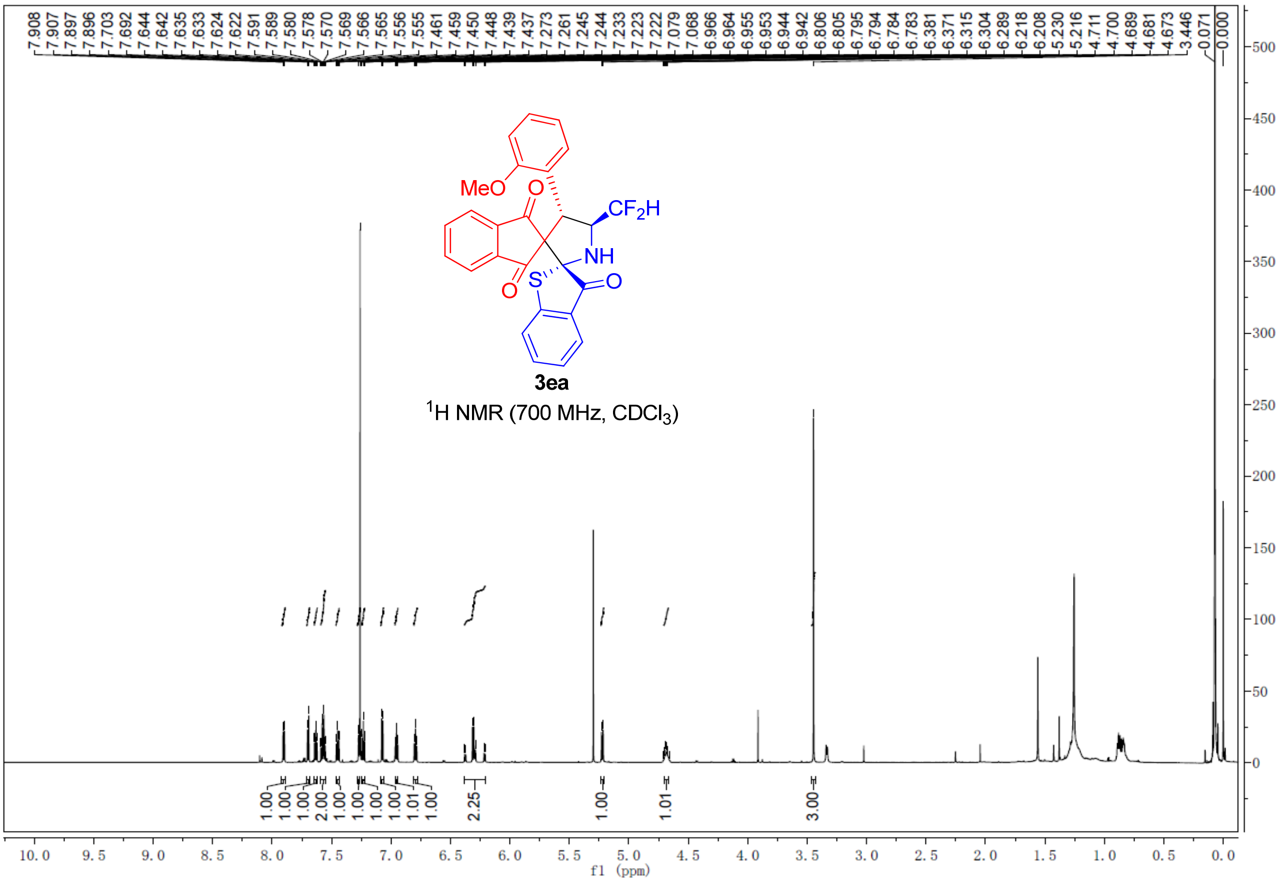

3ea

^{1}H NMR (700 MHz, $CDCl_3$)

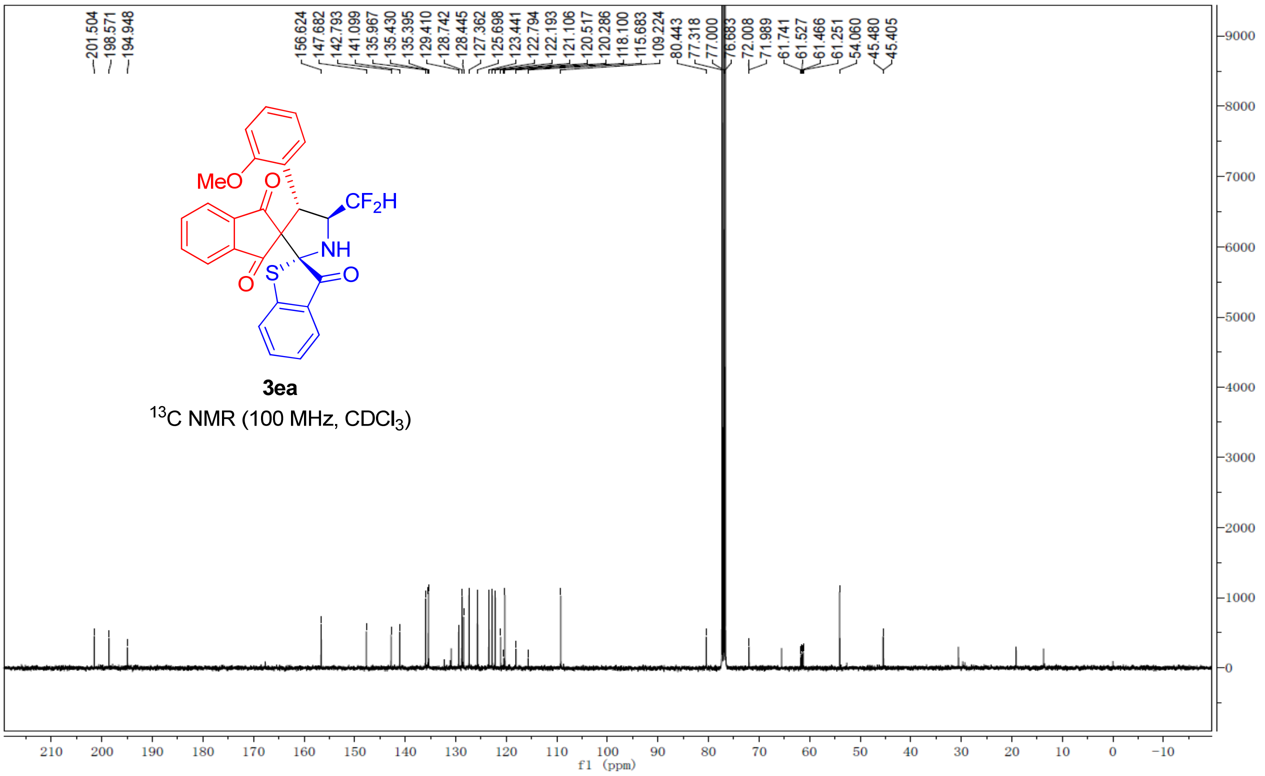

3ea

^{13}C NMR (100 MHz, $CDCl_3$)

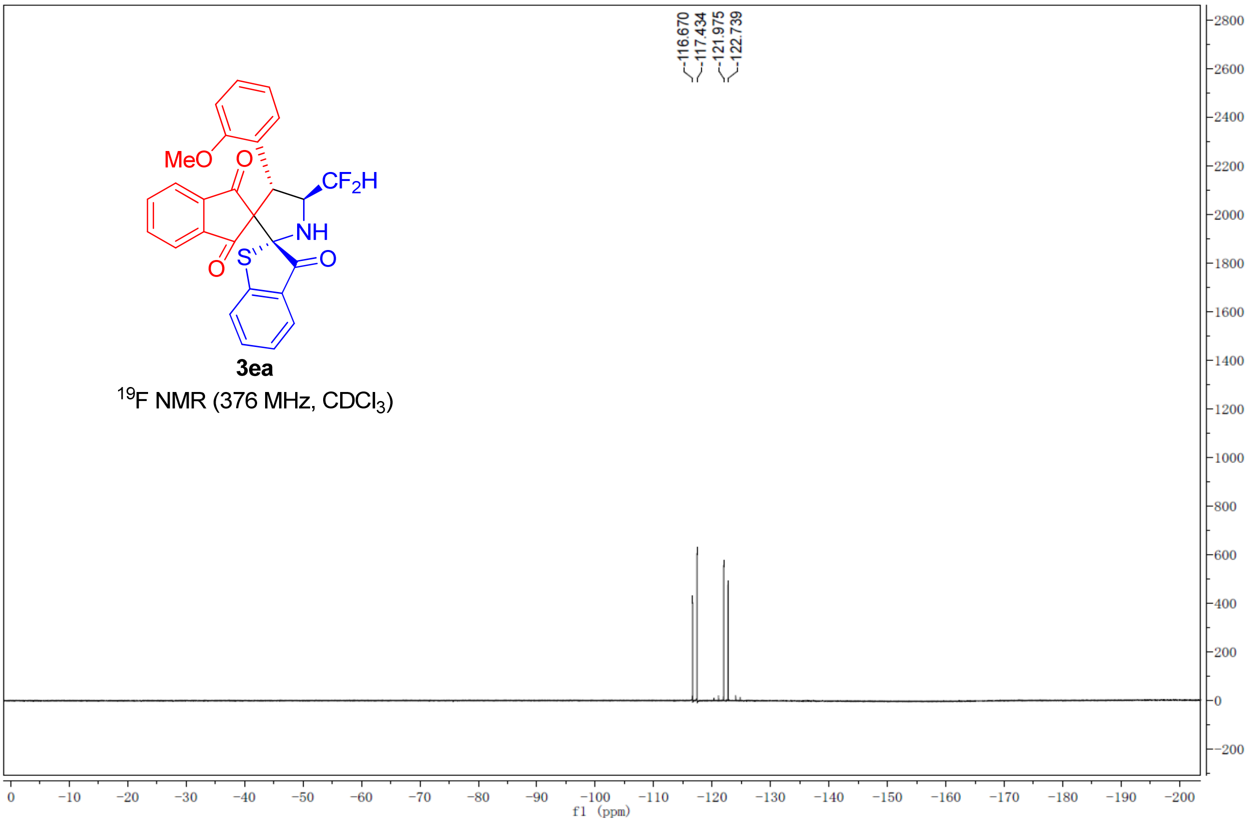

-116.670
-117.434
-121.975
-122.739
MeO
O
CF2H
NH
S
O
O
3ea
19F NMR (376 MHz, CDCl3)
f1 (ppm)

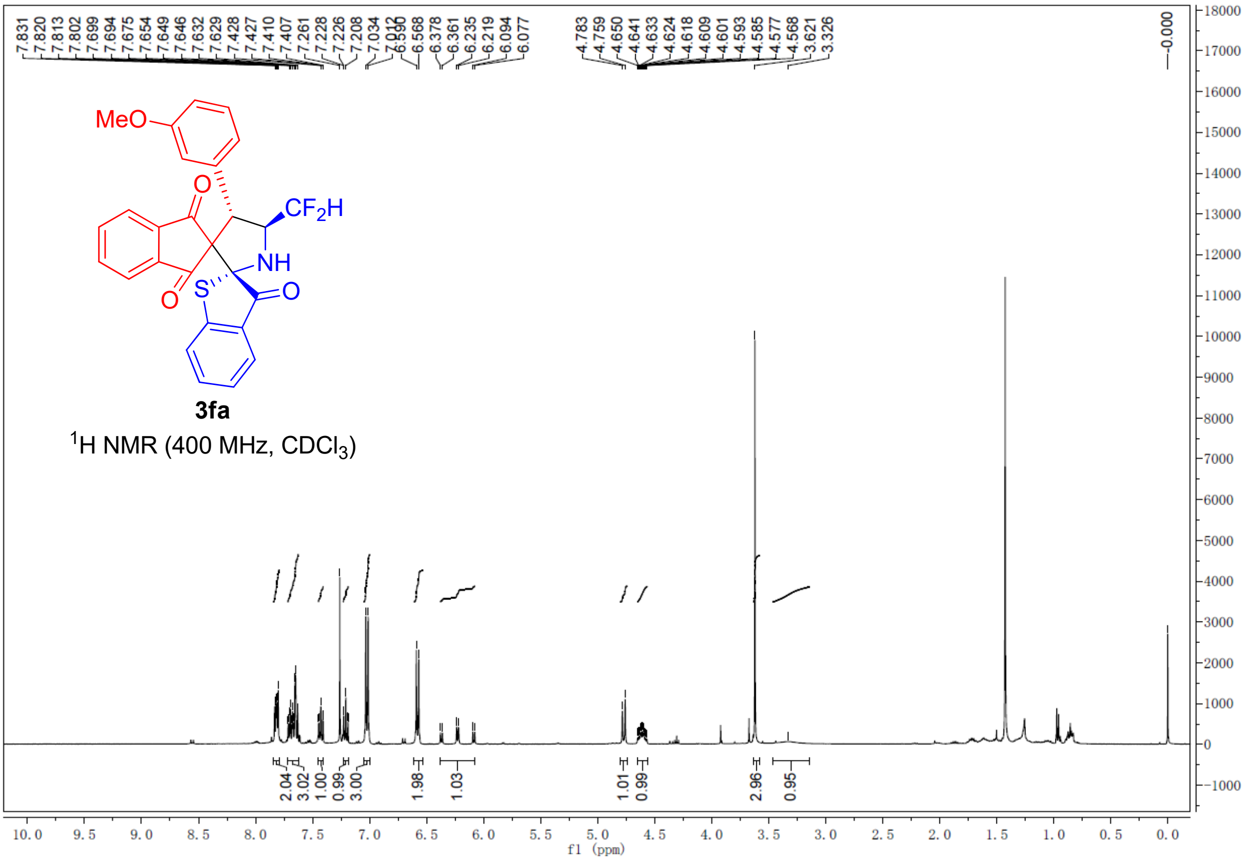

7.831
7.820
7.813
7.802
7.699
7.694
7.675
7.654
7.649
7.646
7.632
7.629
7.428
7.427
7.410
7.407
7.261
7.228
7.226
7.208
7.034
7.012
6.590
6.568
6.378
6.361
6.235
6.219
6.094
6.077
4.783
4.759
4.650
4.641
4.633
4.624
4.618
4.609
4.601
4.593
4.585
4.577
4.568
3.621
3.326
-0.000
MeO
O
CF2H
NH
S
O
O
3fa
1H NMR (400 MHz, CDCl3)
2.04
3.02
1.00
0.99
3.00
1.98
1.03
1.01
0.99
2.96
0.95
f1 (ppm)

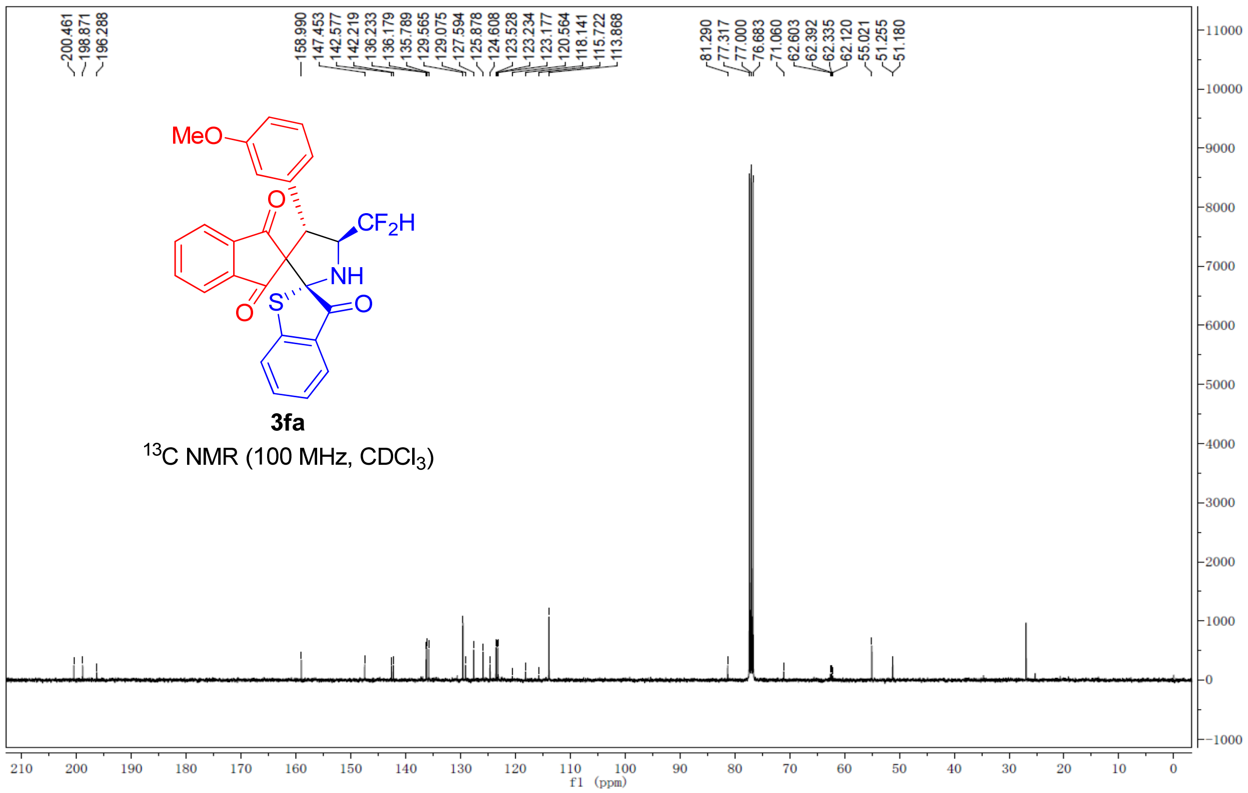
200.461
198.871
196.288
158.990
147.453
142.577
142.219
136.233
136.179
135.789
129.565
129.075
127.594
125.878
124.608
123.528
123.234
123.177
120.564
118.141
115.722
113.868
81.290
77.317
77.000
76.683
71.060
62.603
62.392
62.335
62.120
55.021
51.255
51.180
MeO
O
CF_2H
NH
S
O
O
3fa
^{13}C NMR (100 MHz, $CDCl_3$)
f1 (ppm)

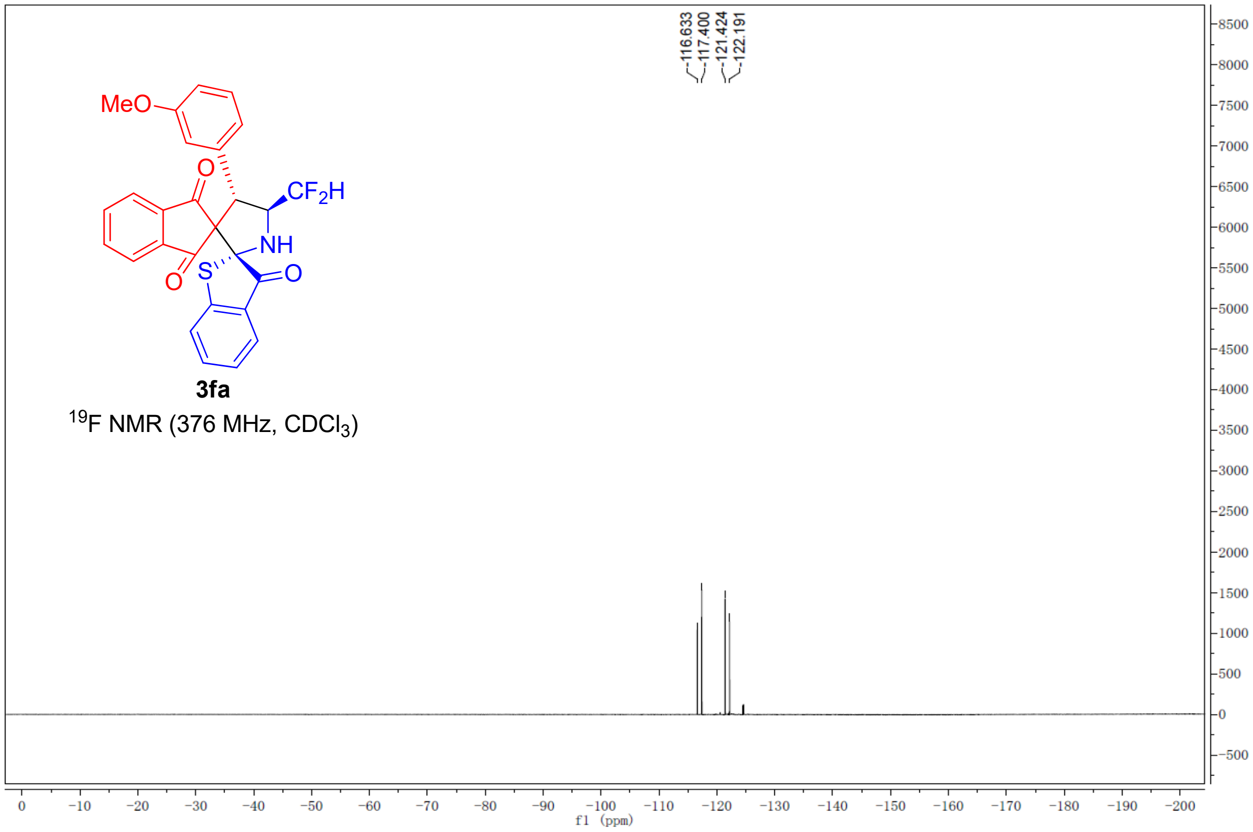
-116.633
-117.400
-121.424
-122.191
MeO
O
CF_2H
NH
S
O
O
3fa
^{19}F NMR (376 MHz, $CDCl_3$)
f1 (ppm)

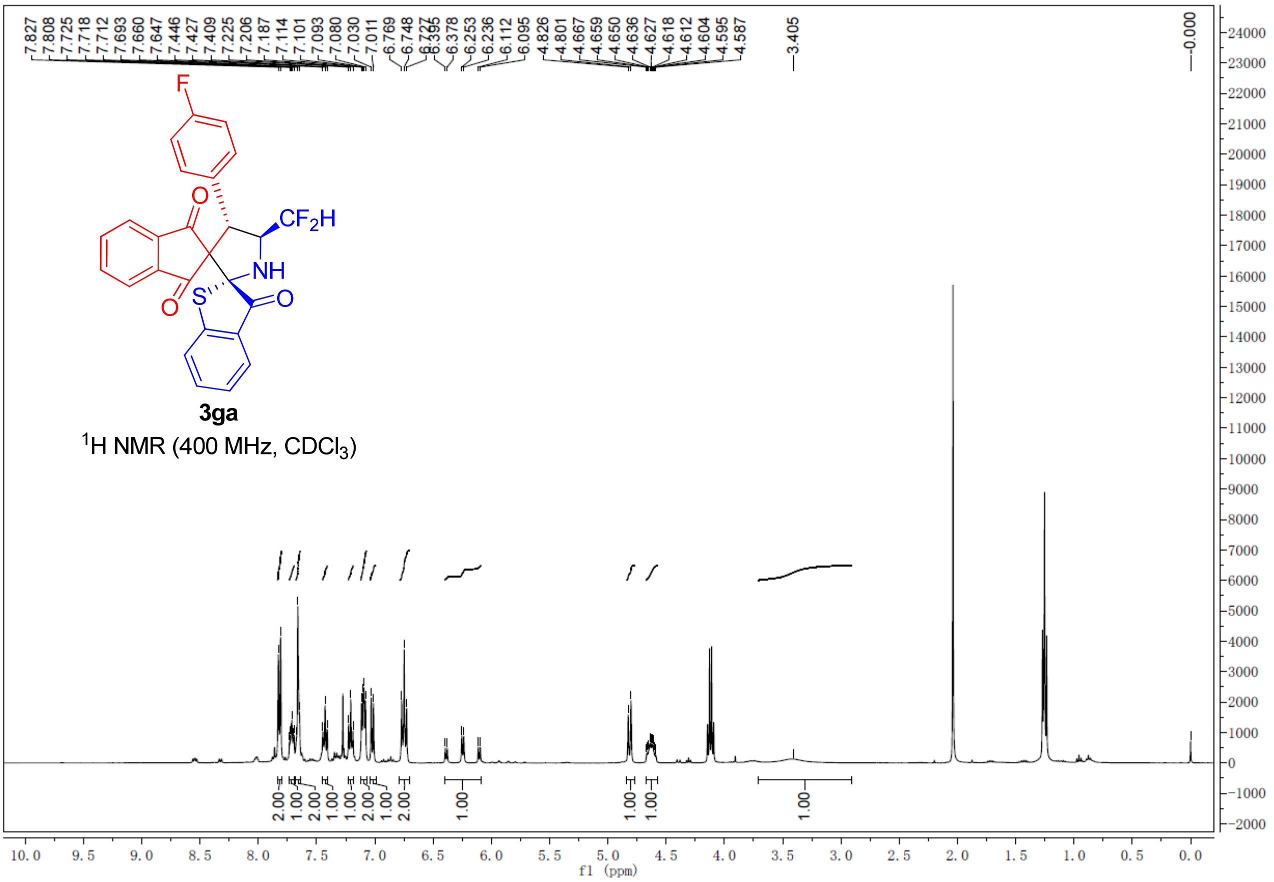

7.827 7.808 7.725 7.718 7.712 7.693 7.660 7.647 7.446 7.427 7.409 7.225 7.206 7.187 7.114 7.101 7.093 7.080 7.030 7.011 6.769 6.748 6.727 6.395 6.378 6.253 6.236 6.112 6.095 4.826 4.801 4.667 4.659 4.650 4.636 4.627 4.618 4.612 4.604 4.595 4.587
3.405
0.000
F
O
CF2H
NH
S
O
O
3ga
1H NMR (400 MHz, CDCl3)
2.00 1.00 2.00 1.00 1.00 2.00 1.00 2.00 1.00 1.00 1.00 1.00
f1 (ppm)

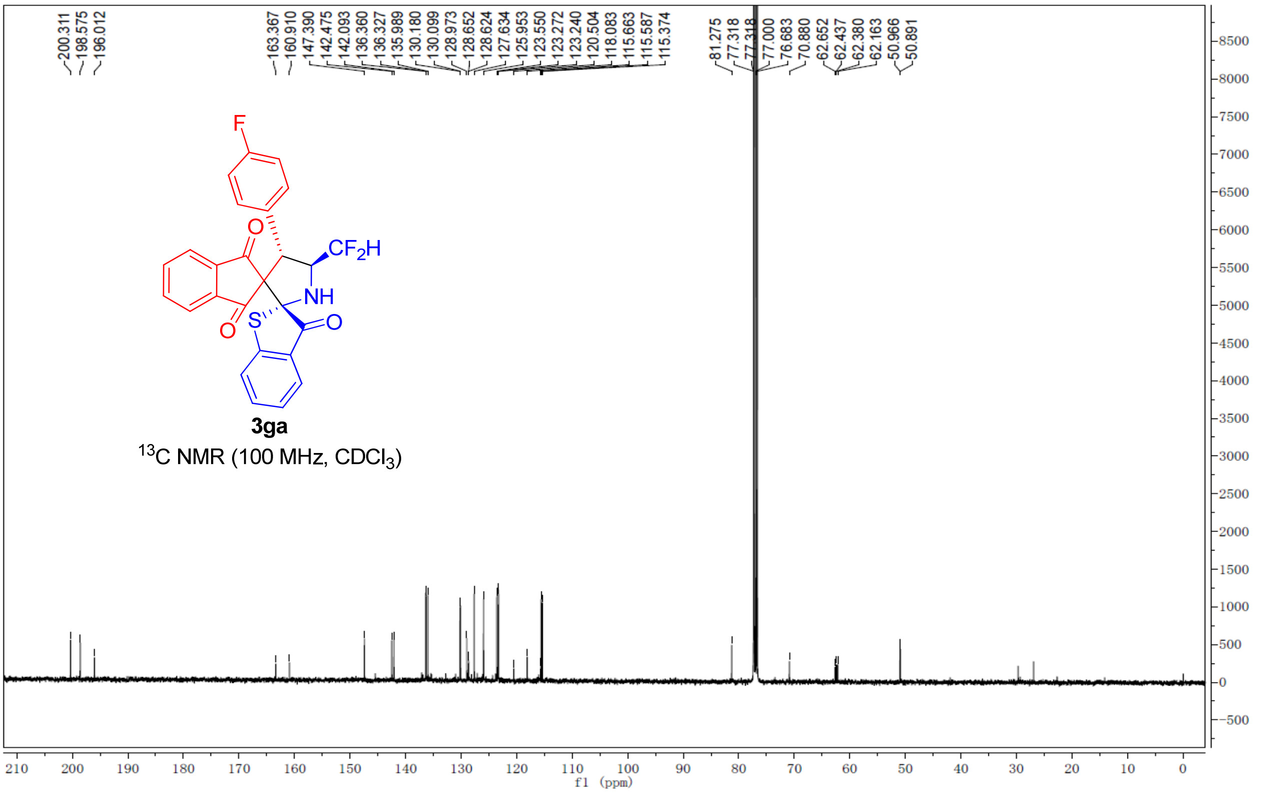

200.311 198.575 196.012
163.367 160.910 147.390 142.475 142.093 136.360 136.327 135.989 130.180 130.099 128.973 128.652 128.624 127.634 125.953 123.550 123.272 123.240 120.504 118.083 115.663 115.587 115.374
81.275 77.318 77.318 77.000 76.683 70.880 62.652 62.437 62.380 62.163 50.966 50.891
F
O
CF2H
NH
S
O
O
3ga
13C NMR (100 MHz, CDCl3)
f1 (ppm)

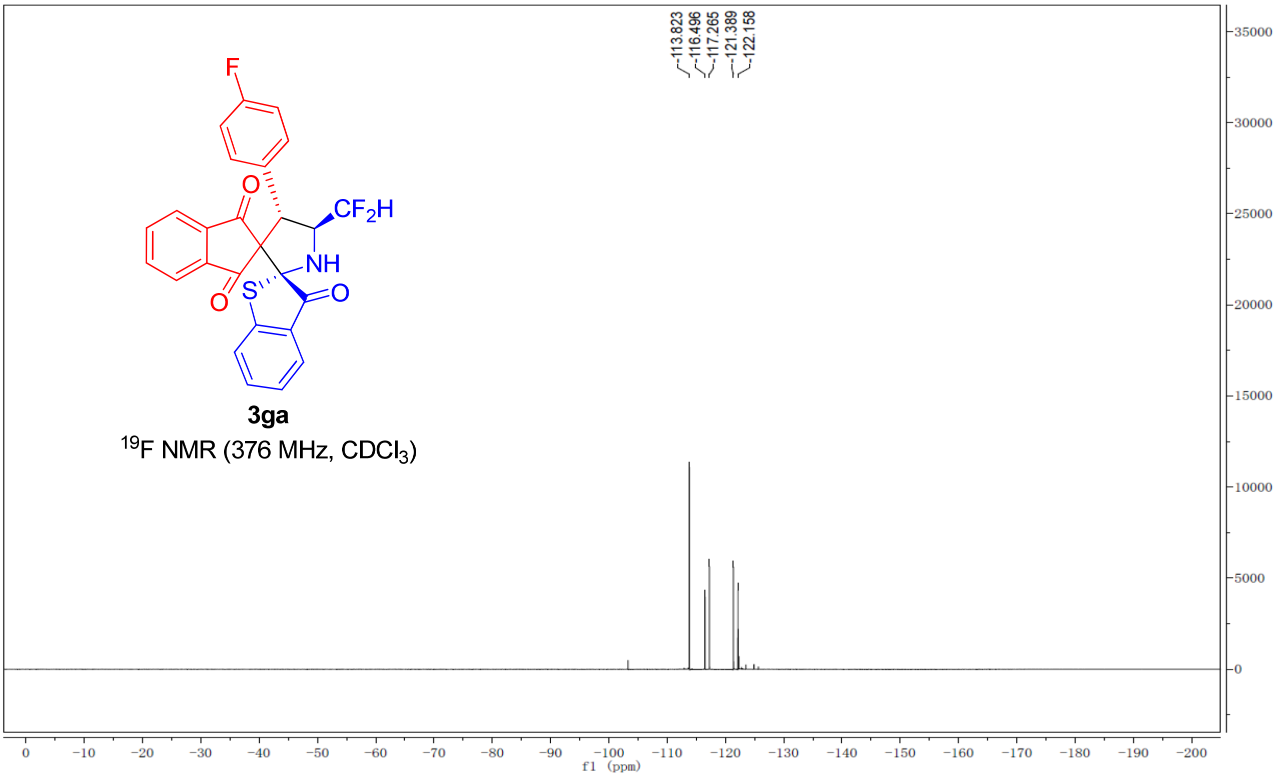
-113.823
-116.496
-117.265
-121.389
-122.158
3ga
^{19}F NMR (376 MHz, CDCl$_3$)
f1 (ppm)

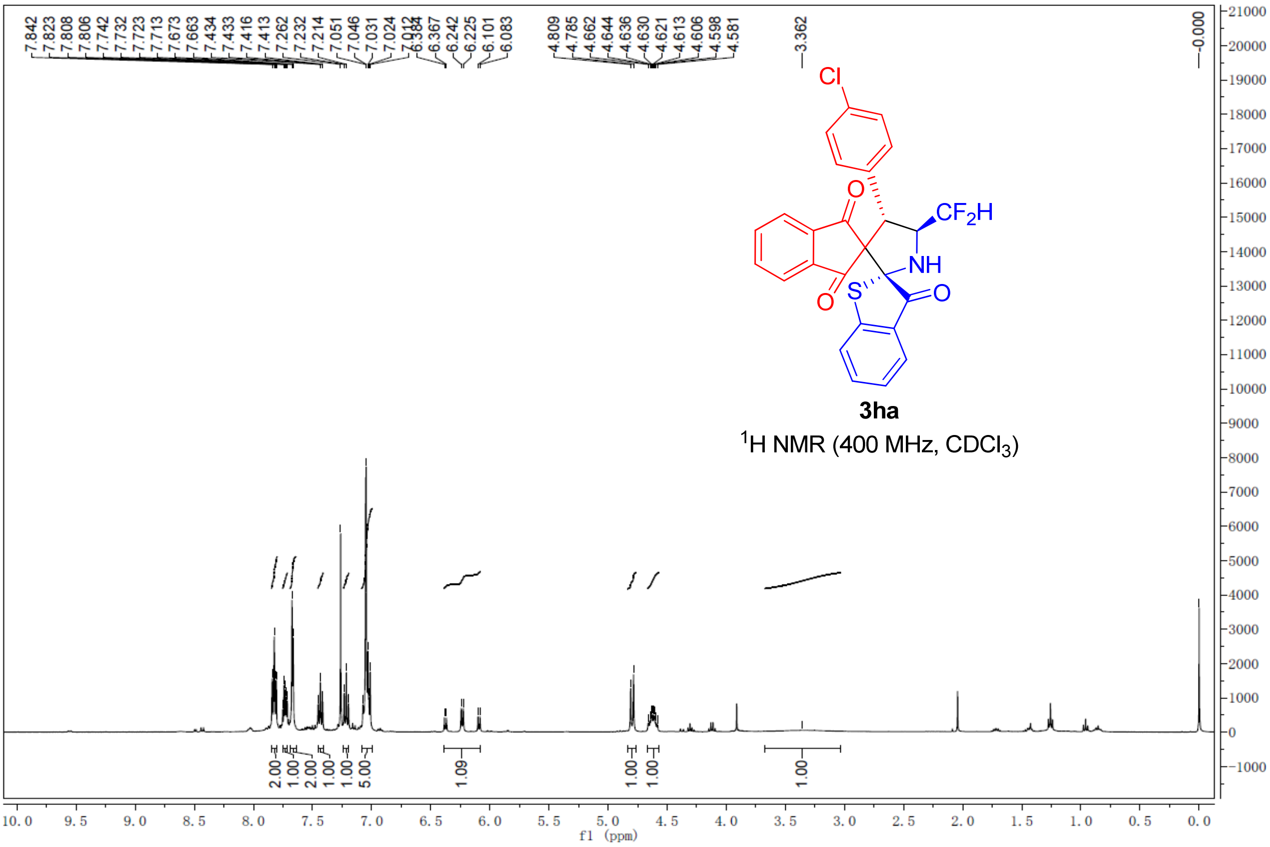
7.842
7.823
7.808
7.806
7.742
7.732
7.723
7.713
7.673
7.663
7.434
7.433
7.416
7.413
7.262
7.232
7.214
7.051
7.046
7.031
7.024
6.367
6.242
6.225
6.101
6.083
4.809
4.785
4.662
4.644
4.636
4.630
4.621
4.613
4.606
4.598
4.581
3.362
-0.000
3ha
^{1}H NMR (400 MHz, CDCl$_3$)
f1 (ppm)

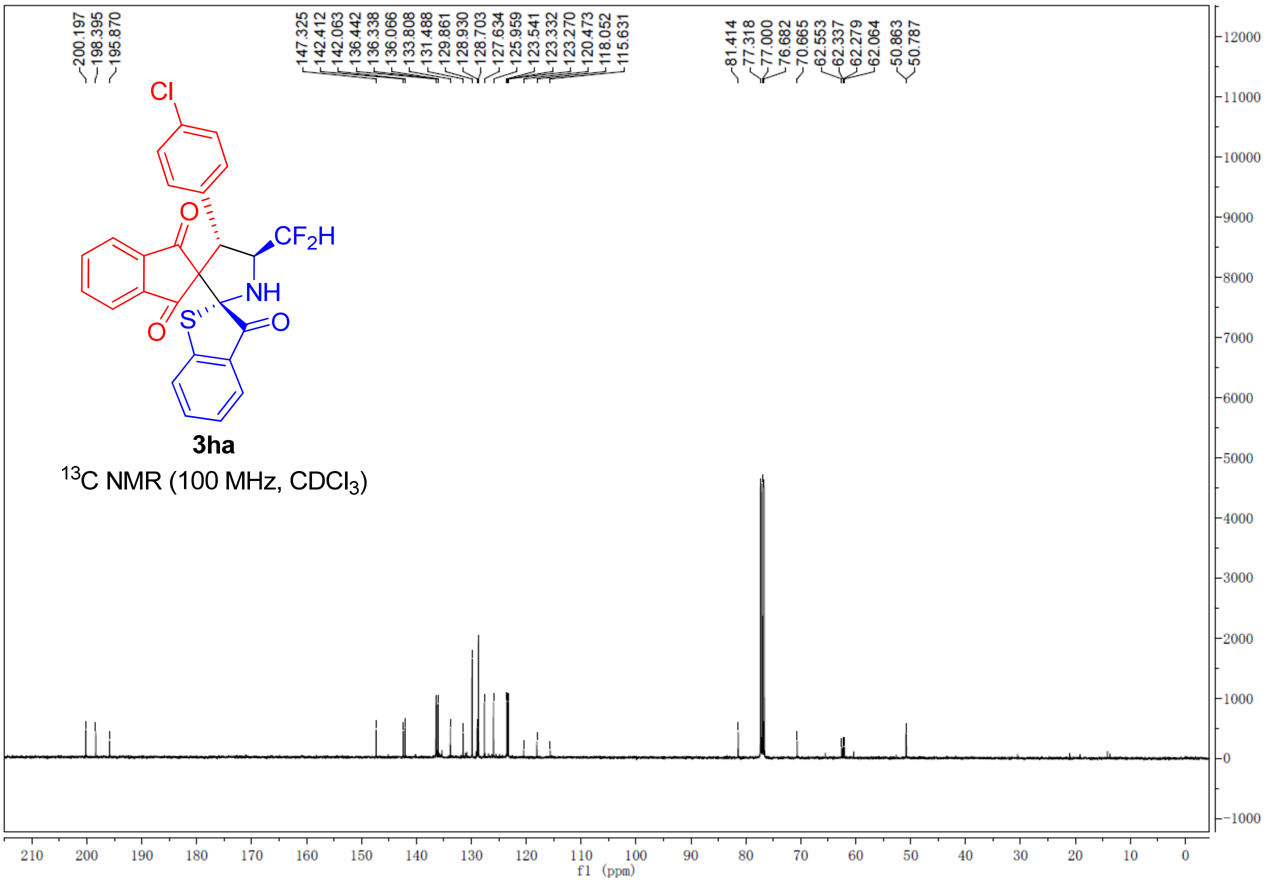

200.197
198.395
195.870
147.325
142.412
142.063
136.442
136.338
136.066
133.808
131.488
129.861
128.930
128.703
127.634
125.959
123.541
123.332
123.270
120.473
118.052
115.631
81.414
77.318
77.000
76.682
70.665
62.553
62.337
62.279
62.064
50.863
50.787
Cl
O
O
CF2H
NH
S
O
3ha
13C NMR (100 MHz, CDCl3)
f1 (ppm)

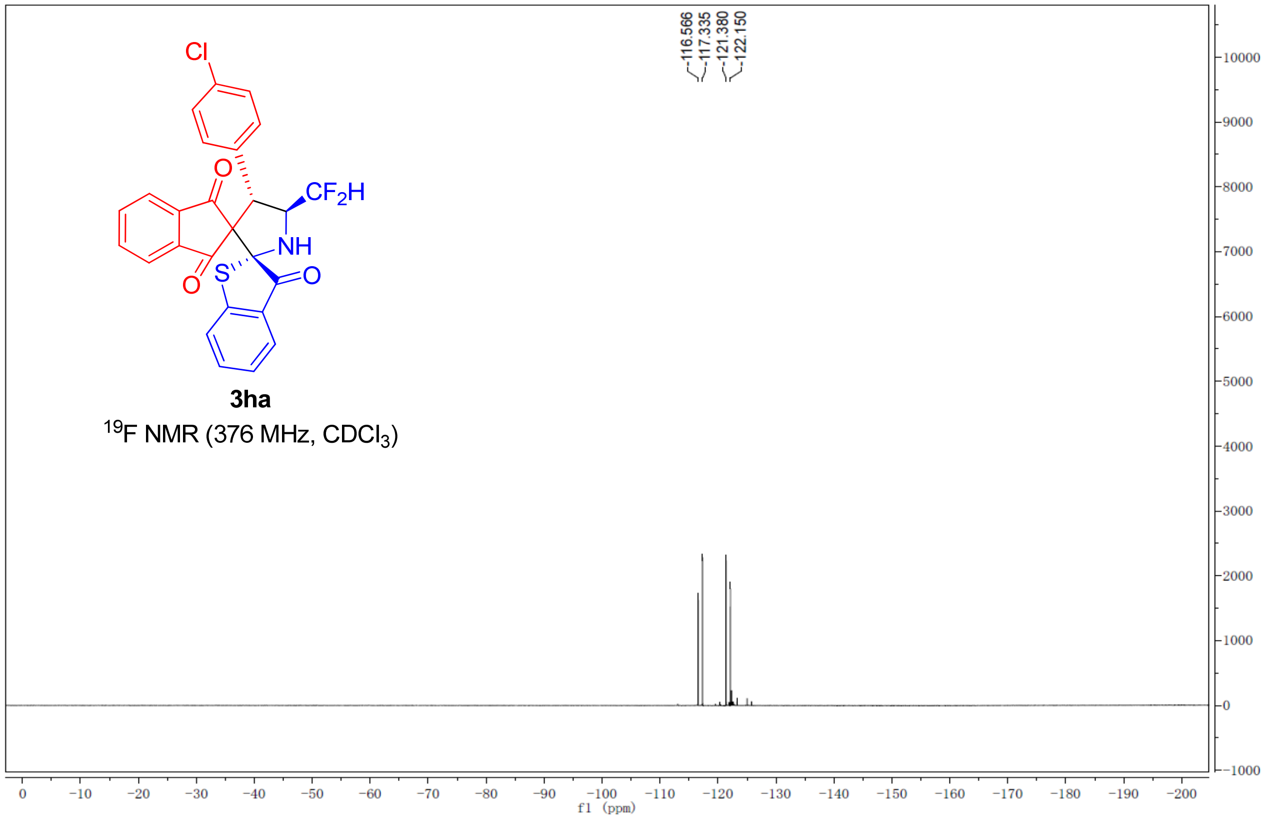

-116.566
-117.335
-121.380
-122.150
Cl
O
O
CF2H
NH
S
O
3ha
19F NMR (376 MHz, CDCl3)
f1 (ppm)

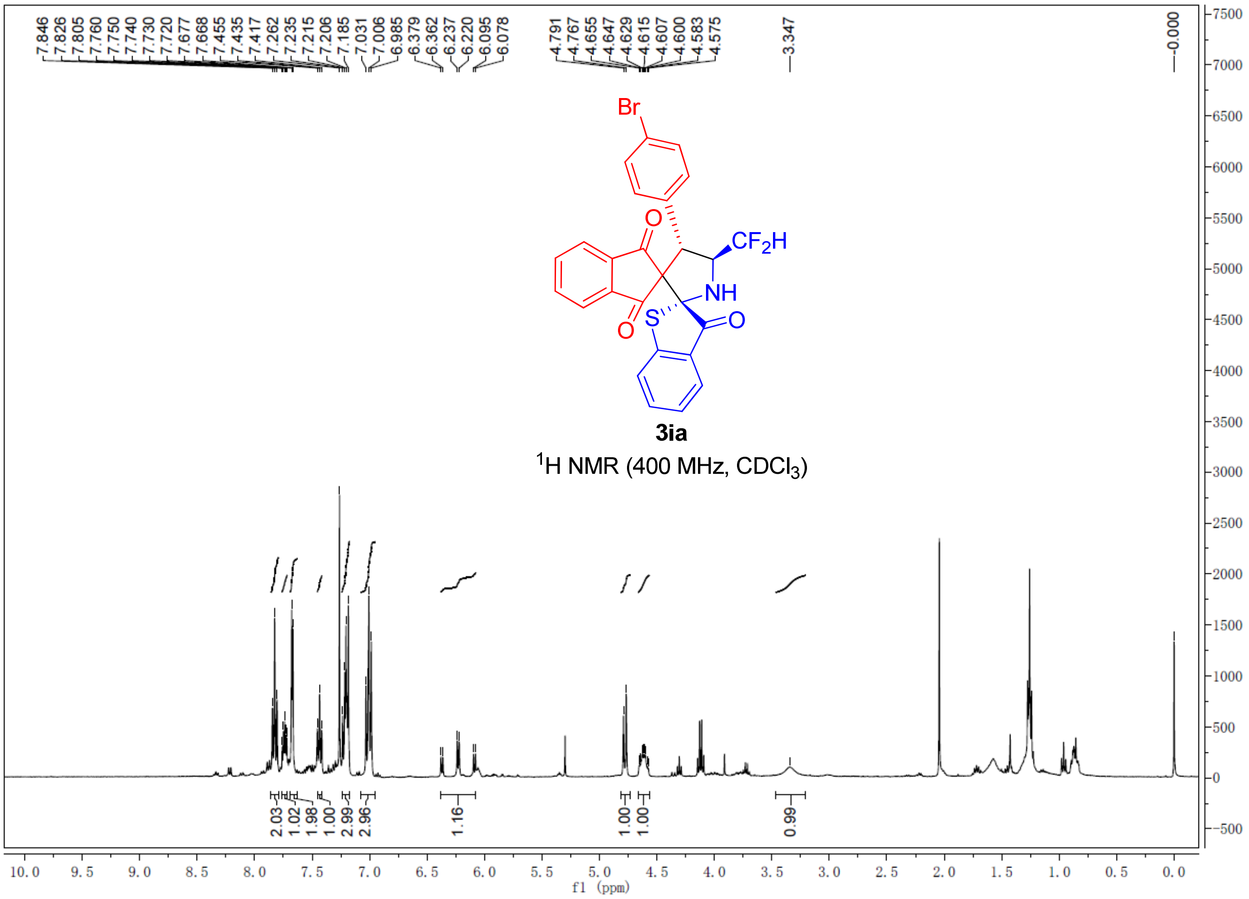
7.846
7.826
7.805
7.760
7.750
7.740
7.730
7.720
7.677
7.668
7.455
7.435
7.417
7.262
7.235
7.215
7.206
7.185
7.031
7.006
6.985
6.379
6.362
6.237
6.220
6.095
6.078
4.791
4.767
4.655
4.647
4.629
4.615
4.607
4.600
4.583
4.575
3.347
0.000
Br
O
CF2H
NH
S
O
O
3ia
1H NMR (400 MHz, CDCl3)
2.03
1.02
1.98
1.00
2.99
2.96
1.16
1.00
1.00
0.99
f1 (ppm)

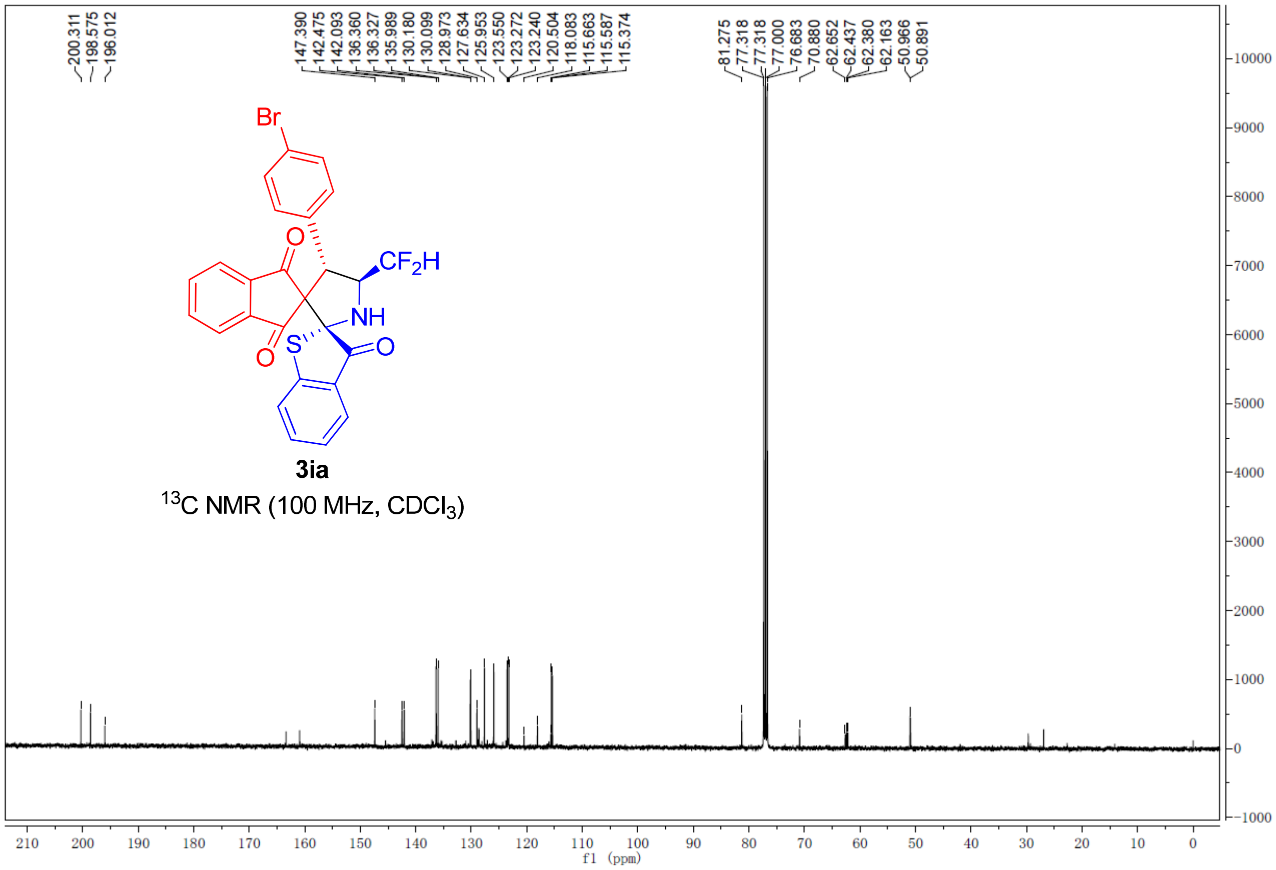
200.311
198.575
196.012
147.390
142.475
142.093
136.360
136.327
135.989
130.180
130.099
128.973
127.634
125.953
123.550
123.272
123.240
120.504
118.083
115.663
115.587
115.374
81.275
77.318
77.318
77.000
76.683
70.880
62.652
62.437
62.380
62.163
50.966
50.891
Br
O
CF2H
NH
S
O
O
3ia
13C NMR (100 MHz, CDCl3)
f1 (ppm)

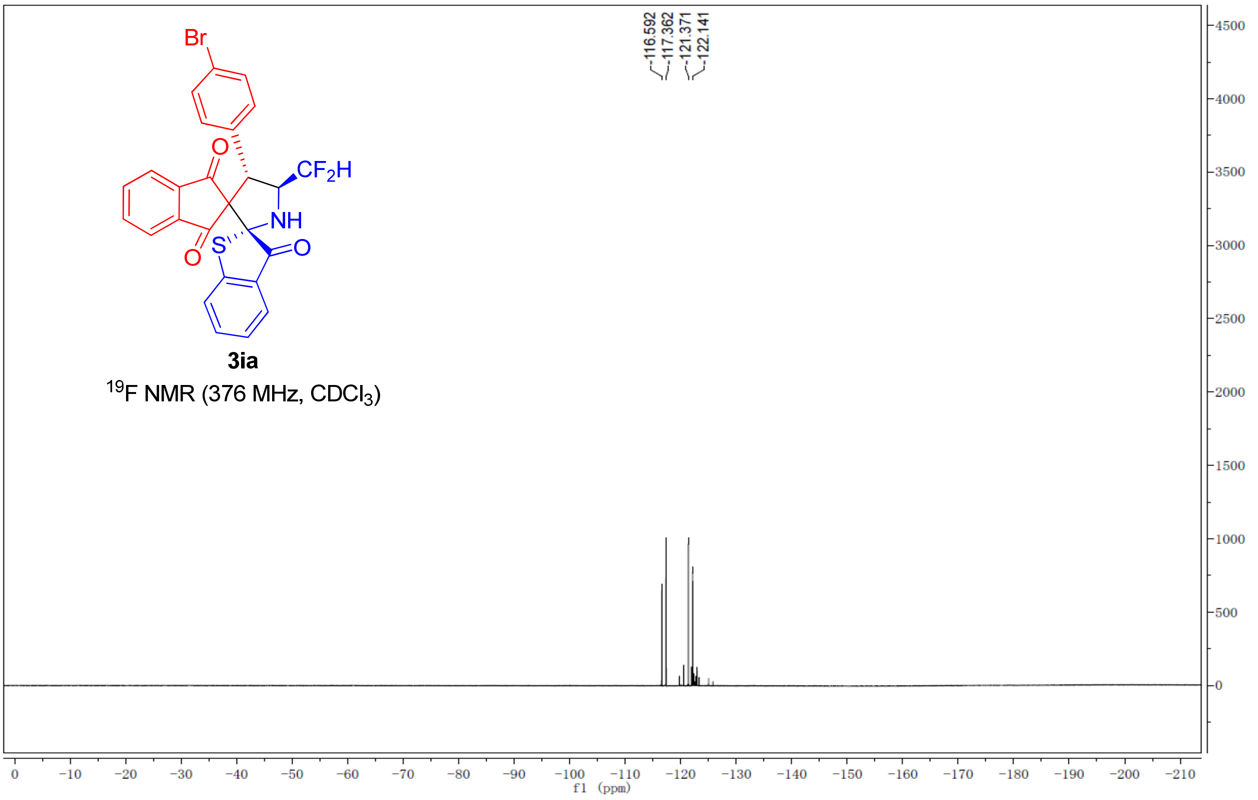
-116.592
-117.362
-121.371
-122.141
Br
O
CF2H
NH
S
O
O
3ia
19F NMR (376 MHz, CDCl3)
f1 (ppm)

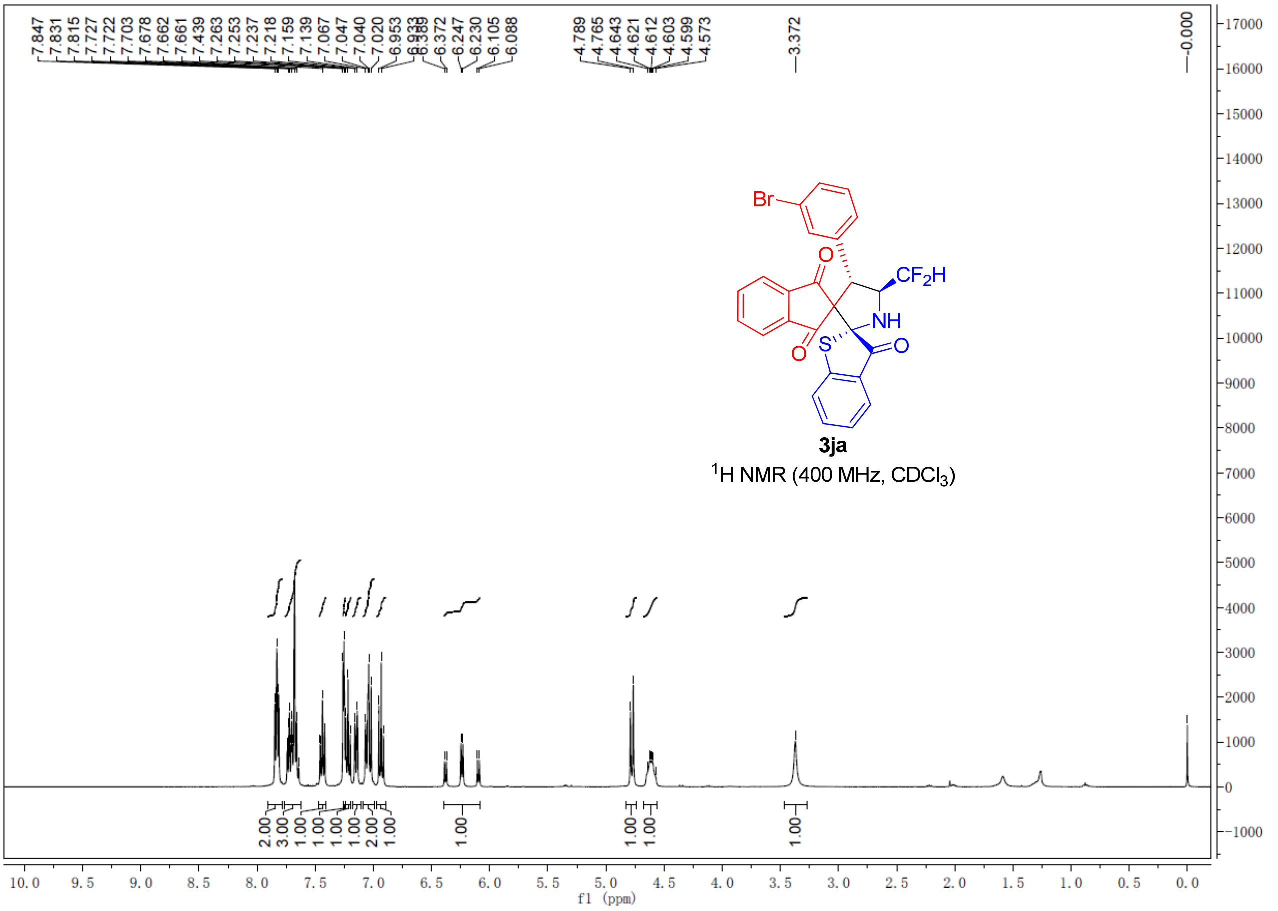
7.847
7.831
7.815
7.727
7.722
7.703
7.678
7.662
7.661
7.439
7.263
7.253
7.237
7.218
7.159
7.139
7.067
7.047
7.040
7.020
6.953
6.933
6.372
6.247
6.230
6.105
6.088
4.789
4.765
4.643
4.621
4.612
4.603
4.599
4.573
3.372
-0.000
Br
O
CF2H
NH
S
O
O
3ja
1H NMR (400 MHz, CDCl3)
2.00
3.00
1.00
1.00
1.00
1.00
1.00
2.00
1.00
1.00
1.00
1.00
1.00
f1 (ppm)

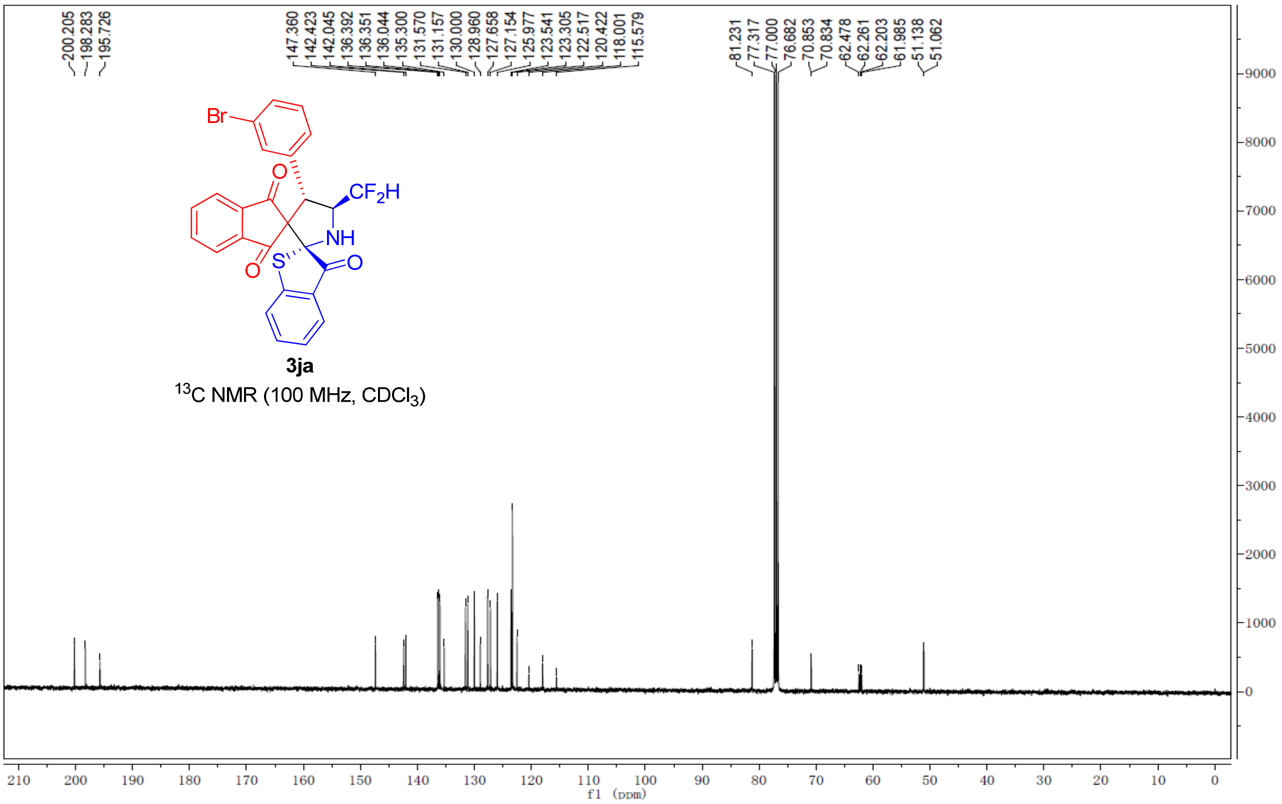
200.205
198.283
195.726
147.360
142.423
142.045
136.392
136.351
136.044
135.300
131.570
131.157
130.000
128.960
127.658
127.154
125.977
123.541
123.305
122.517
120.422
118.001
115.579
81.231
77.317
77.000
76.682
70.853
70.834
62.478
62.261
62.203
61.985
51.138
51.062
Br
O
CF2H
NH
S
O
O
3ja
13C NMR (100 MHz, CDCl3)
f1 (ppm)

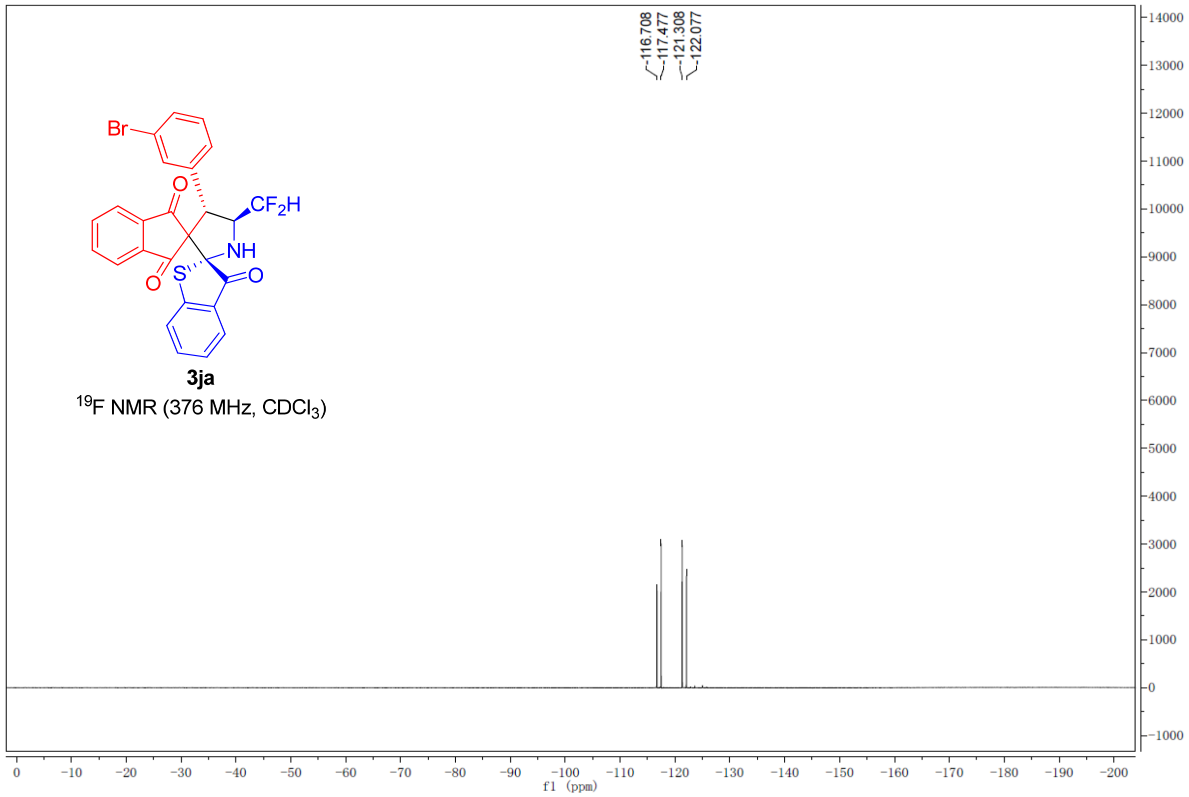
-116.708
-117.477
-121.308
-122.077
Br
O
CF2H
NH
S
O
O
3ja
19F NMR (376 MHz, CDCl3)
f1 (ppm)

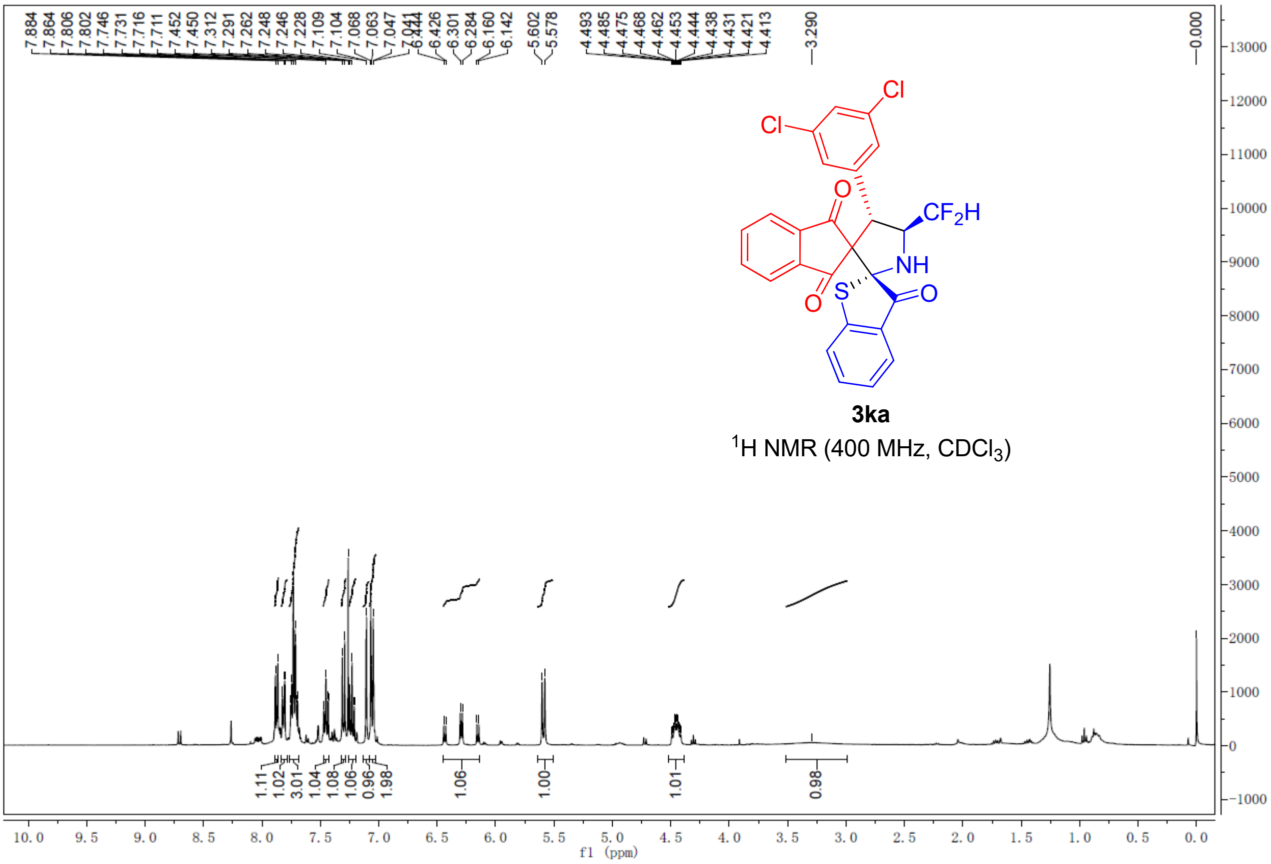
7.884 7.864 7.806 7.802 7.746 7.731 7.716 7.711 7.452 7.450 7.312 7.291 7.262 7.248 7.246 7.228 7.109 7.104 7.068 7.063 7.047 7.041 6.444 6.426 6.301 6.284 6.160 6.142 5.602 5.578 4.493 4.485 4.475 4.468 4.462 4.453 4.444 4.438 4.431 4.421 4.413 3.290 0.000
Cl
Cl
O
CF2H
NH
S
O
O
3ka
1H NMR (400 MHz, CDCl3)
1.11 1.02 3.01 1.04 1.08 1.06 0.96 1.98 1.06 1.00 1.01 0.98
f1 (ppm)

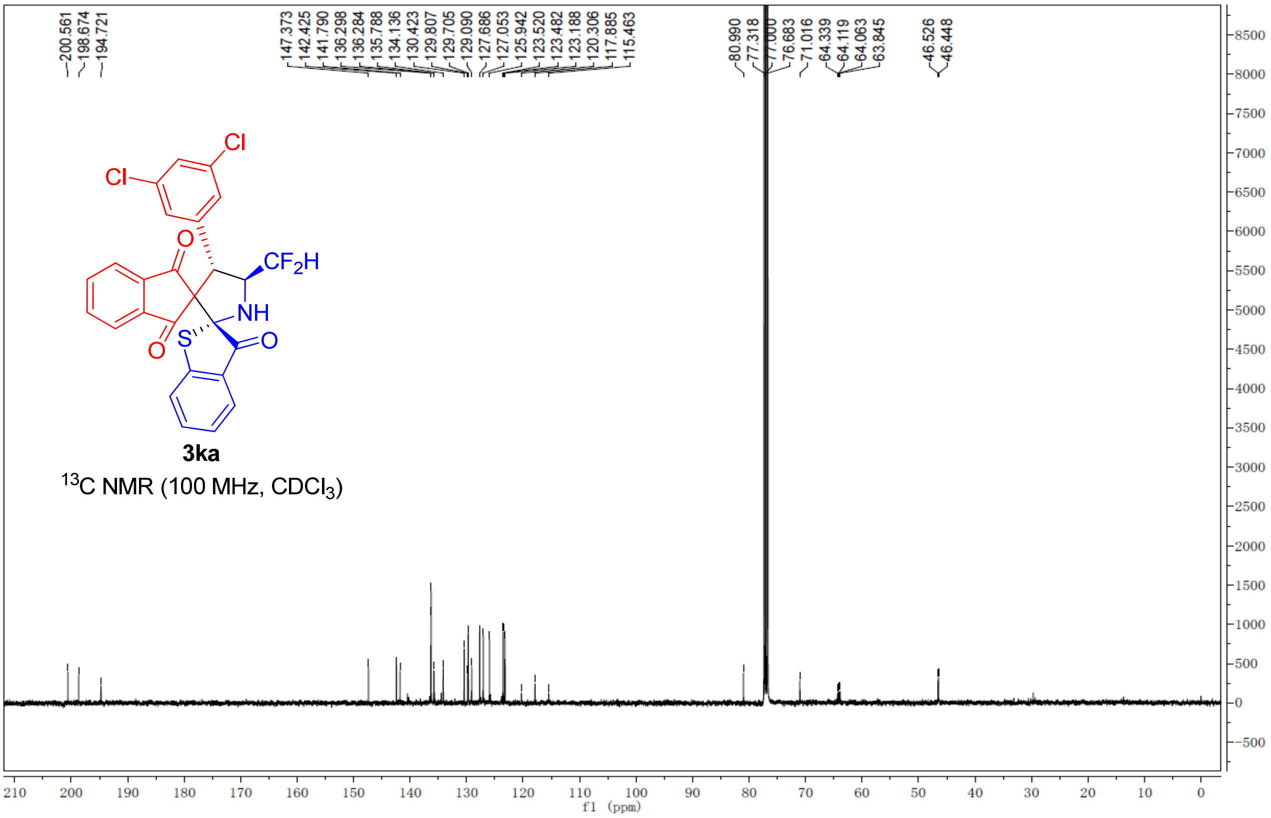
200.561 198.674 194.721 147.373 142.425 141.790 136.298 136.284 135.788 134.136 130.423 129.807 129.705 129.090 127.686 127.053 125.942 123.520 123.482 123.188 120.306 117.885 115.463 80.990 77.318 77.000 76.683 71.016 64.339 64.119 64.063 63.845 46.526 46.448
Cl
Cl
O
CF2H
NH
S
O
O
3ka
13C NMR (100 MHz, CDCl3)
f1 (ppm)

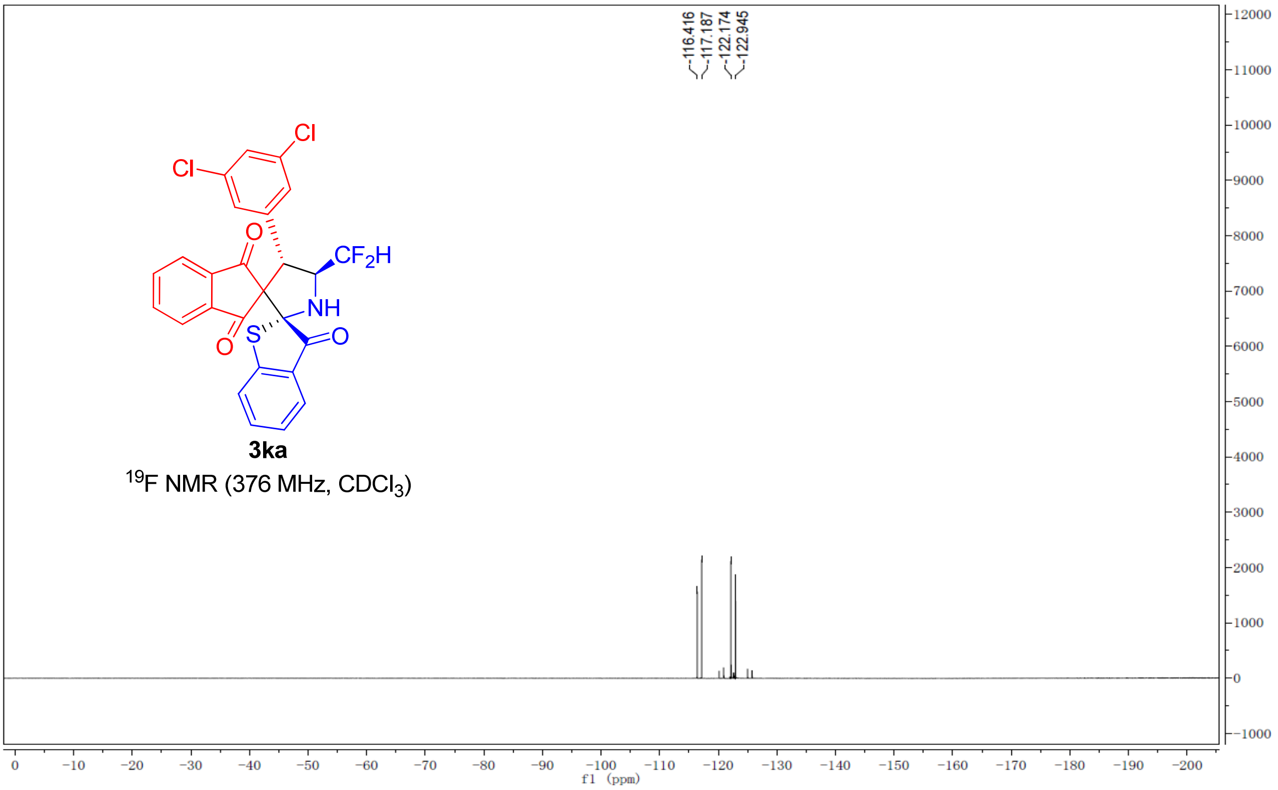

-116.416
-117.187
-122.174
-122.945
3ka
19F NMR (376 MHz, CDCl3)
f1 (ppm)

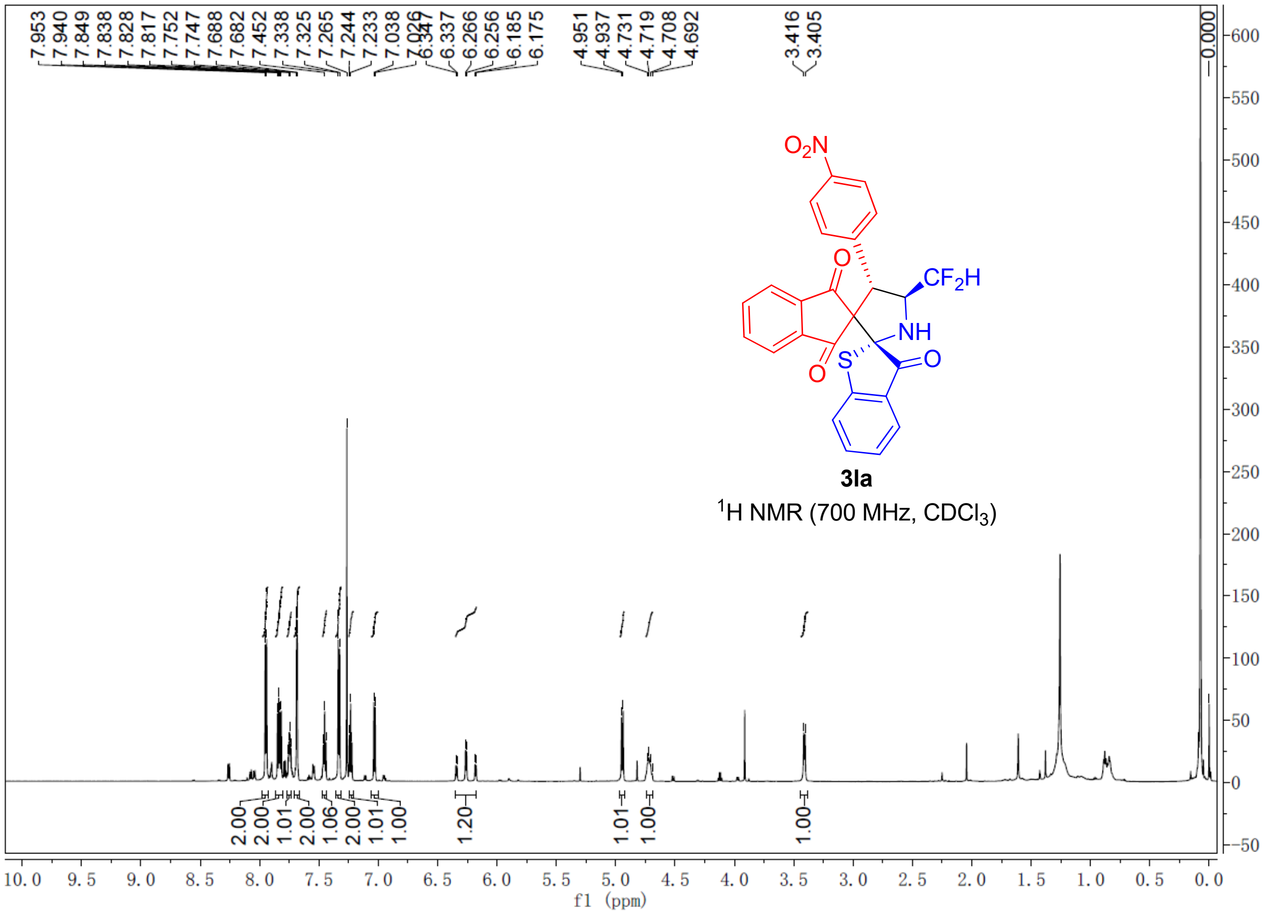

7.953
7.940
7.849
7.838
7.828
7.817
7.752
7.747
7.688
7.682
7.452
7.338
7.325
7.265
7.244
7.233
7.038
7.026
6.347
6.337
6.266
6.256
6.185
6.175
4.951
4.937
4.731
4.719
4.708
4.692
3.416
3.405
0.000
3la
1H NMR (700 MHz, CDCl3)
2.00
2.00
1.01
2.00
1.06
2.00
1.01
1.00
1.20
1.01
1.00
1.00
f1 (ppm)

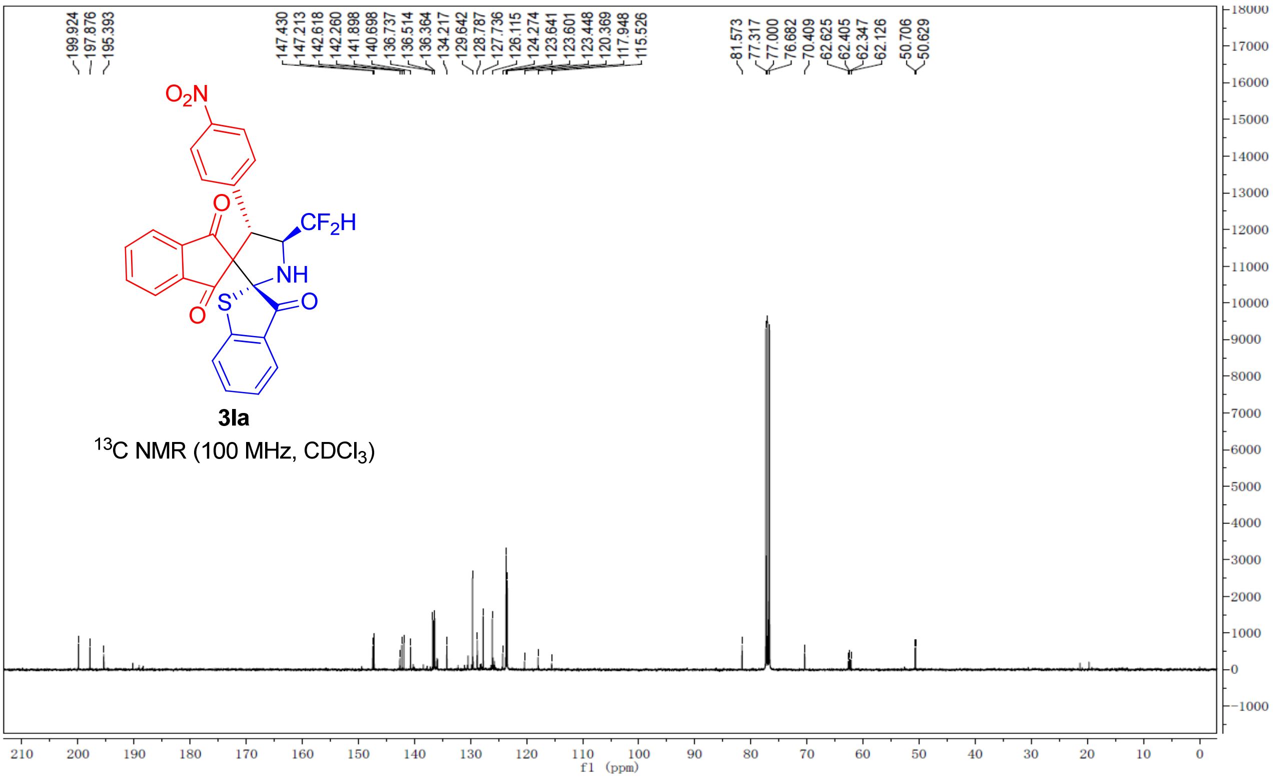
199.924
197.876
195.393
147.430
147.213
142.618
142.260
141.898
140.698
136.737
136.514
136.364
134.217
129.642
128.787
127.736
126.115
124.274
123.641
123.601
123.448
120.369
117.948
115.526
81.573
77.317
77.000
76.682
70.409
62.625
62.405
62.347
62.126
50.706
50.629
O2N
CF2H
NH
O
O
S
O
3la
13C NMR (100 MHz, CDCl3)
f1 (ppm)

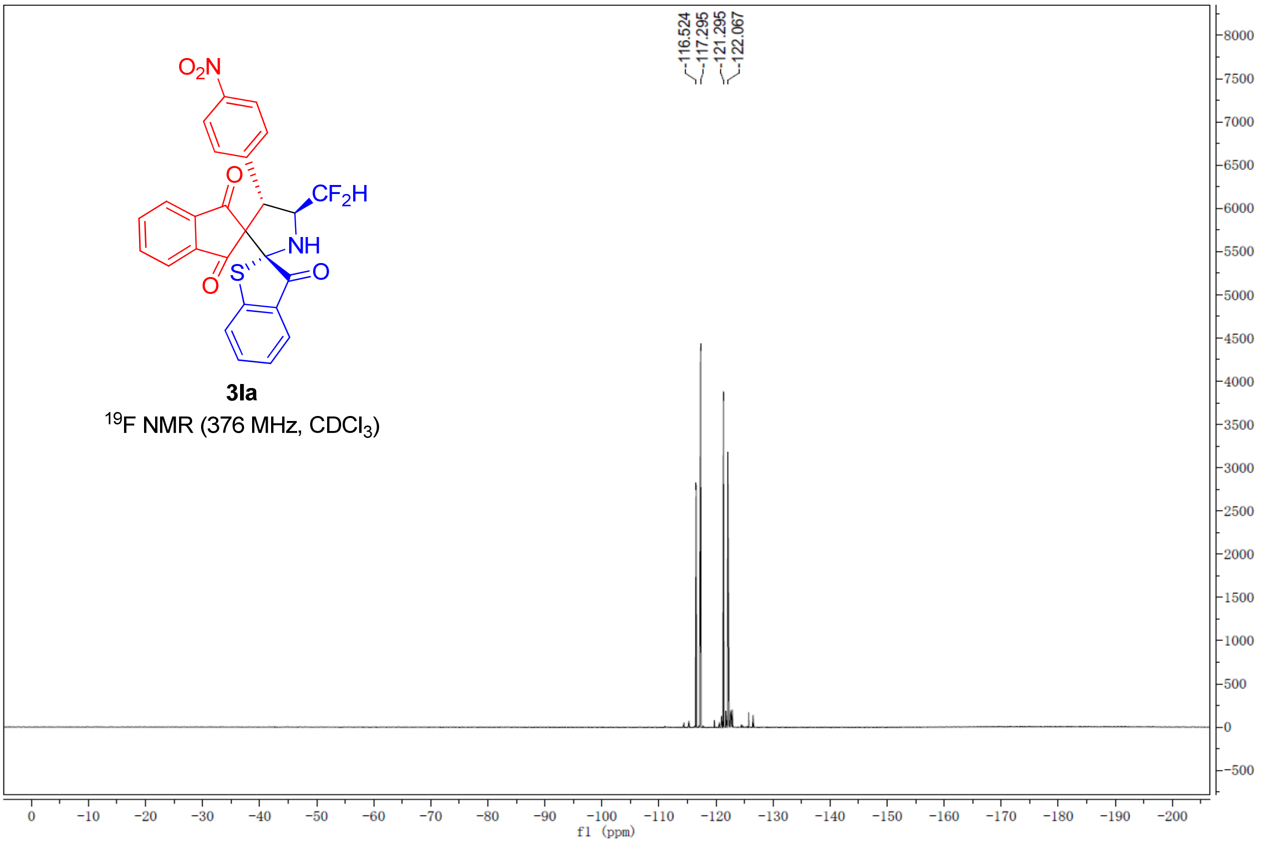
-116.524
-117.295
-121.295
-122.067
O2N
CF2H
NH
O
O
S
O
3la
19F NMR (376 MHz, CDCl3)
f1 (ppm)

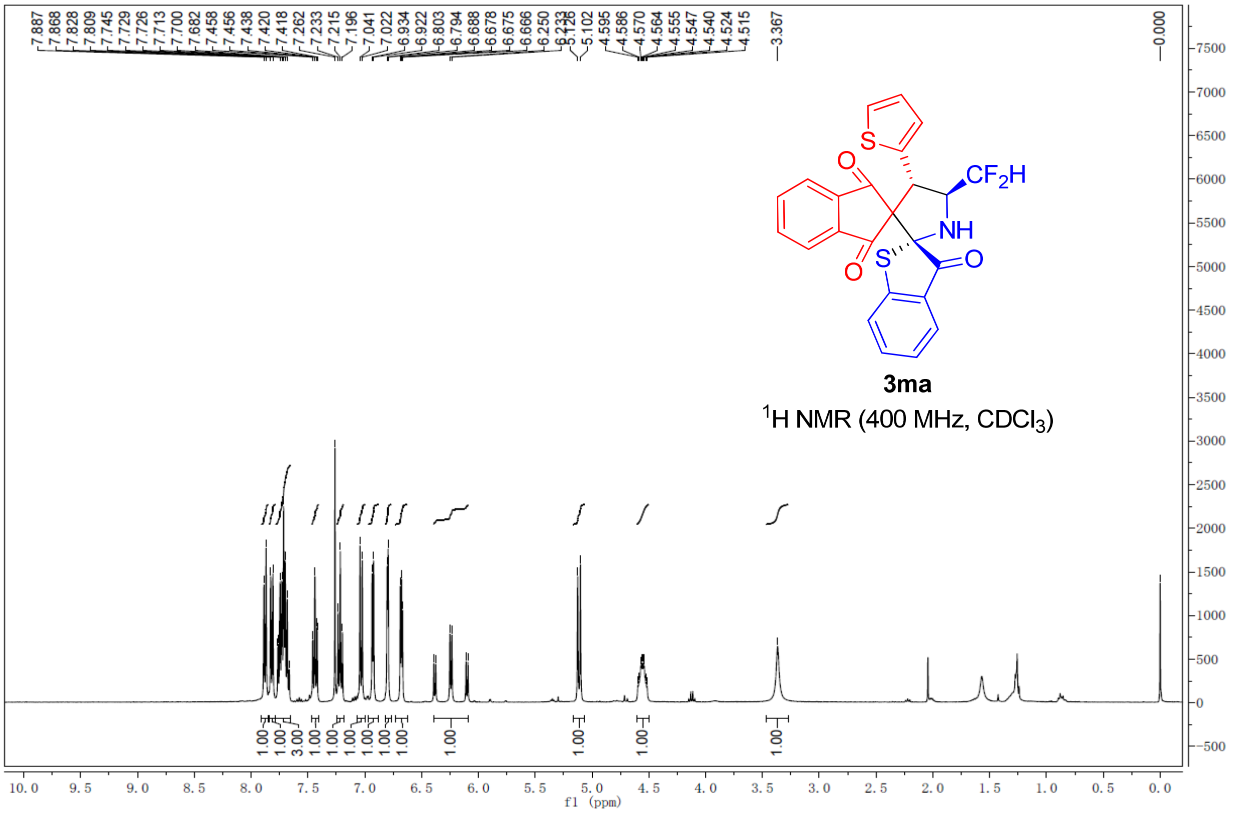
7.887 7.868 7.828 7.809 7.745 7.729 7.726 7.713 7.700 7.682 7.458 7.456 7.438 7.420 7.418 7.262 7.233 7.215 7.196 7.041 7.022 6.934 6.922 6.803 6.794 6.688 6.678 6.675 6.666 6.250 6.233 5.126 5.102 4.595 4.586 4.570 4.564 4.555 4.547 4.540 4.524 4.515 3.367 0.000
O
S
CF2H
NH
S
O
O
3ma
1H NMR (400 MHz, CDCl3)
f1 (ppm)

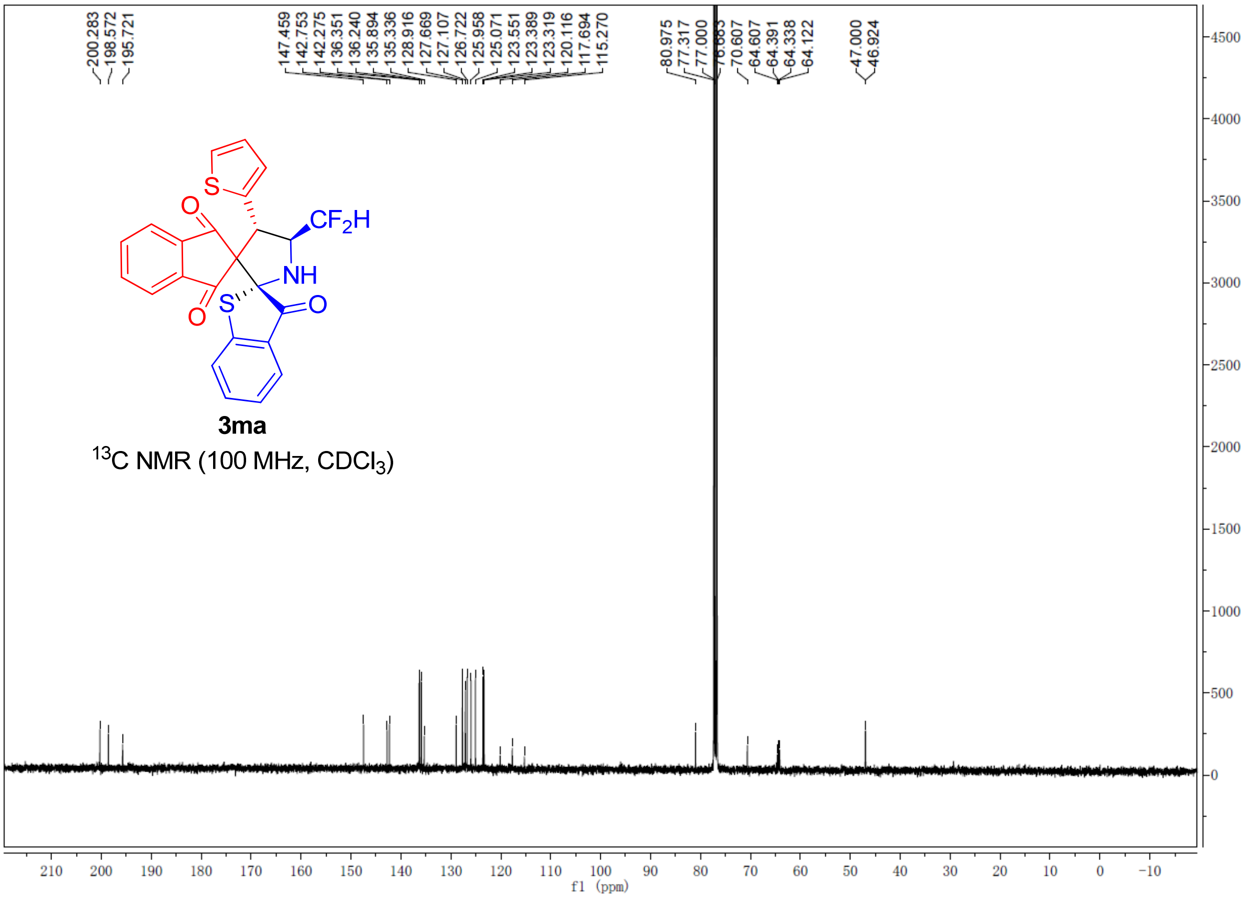
200.283 198.572 195.721 147.459 142.753 142.275 136.351 136.240 135.894 135.336 128.916 127.669 127.107 126.722 125.958 125.071 123.551 123.389 123.319 120.116 117.694 115.270 80.975 77.317 77.000 76.683 70.607 64.607 64.391 64.338 64.122 47.000 46.924
S
O
CF2H
NH
S
O
O
3ma
13C NMR (100 MHz, CDCl3)
f1 (ppm)

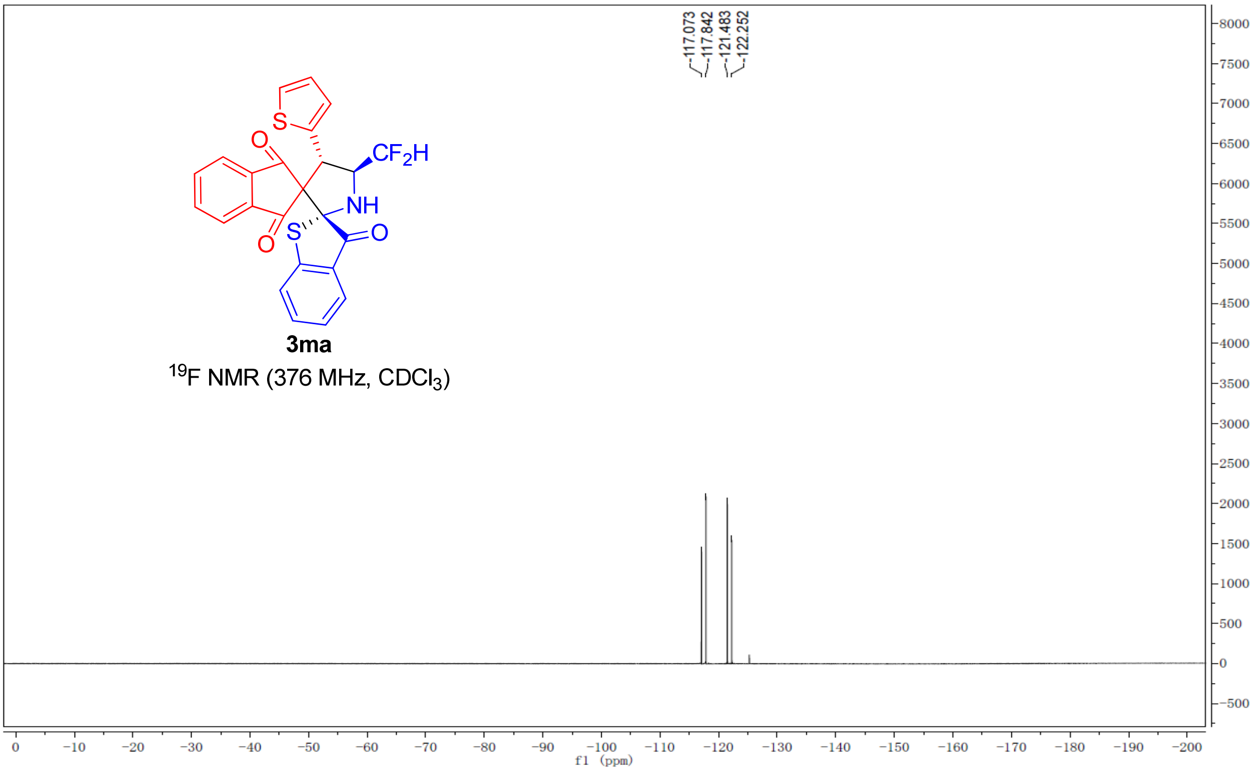
-117.073
-117.842
-121.483
-122.252
CF2H
NH
3ma
19F NMR (376 MHz, CDCl3)
f1 (ppm)

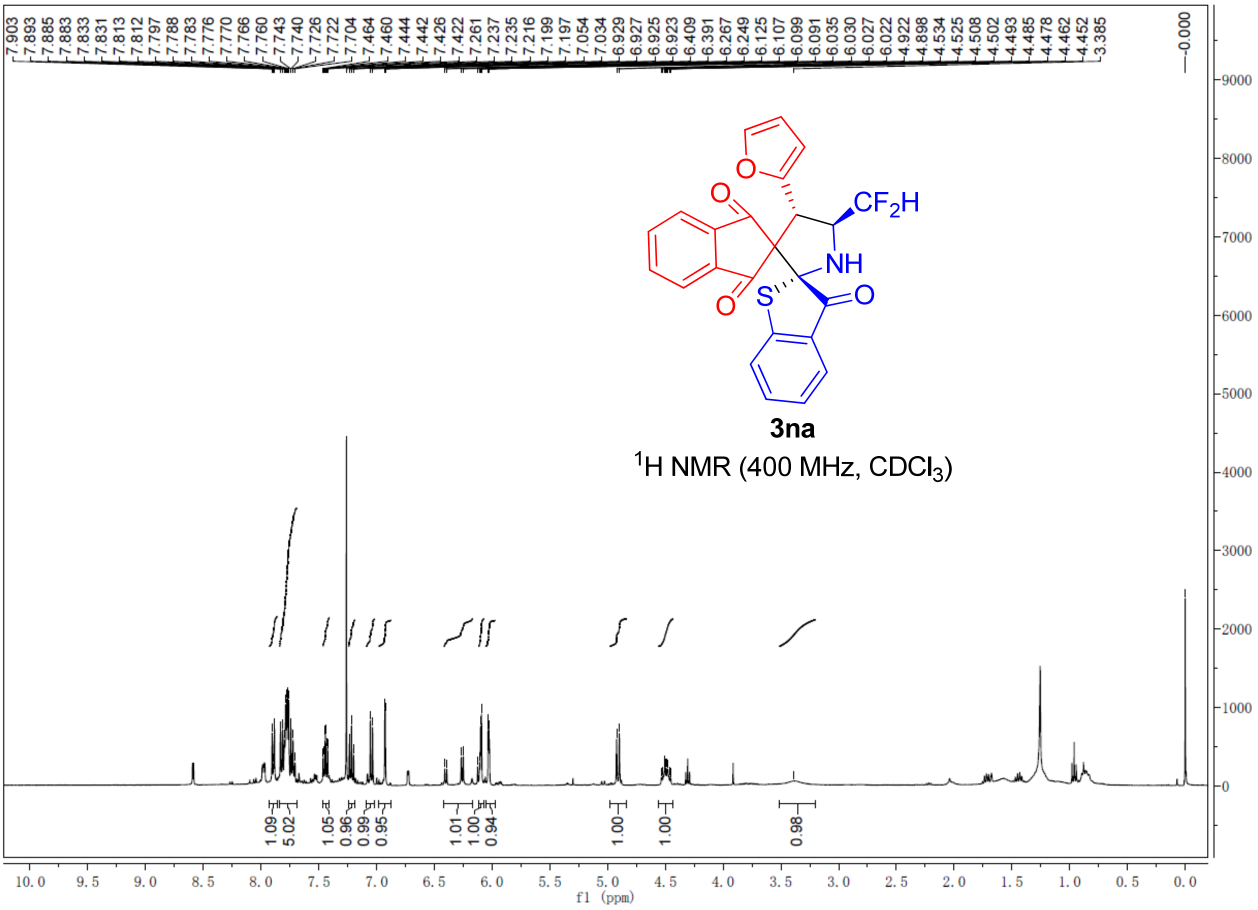
7.903
7.893
7.885
7.883
7.833
7.831
7.813
7.812
7.797
7.788
7.783
7.776
7.770
7.766
7.760
7.743
7.740
7.726
7.722
7.704
7.464
7.460
7.444
7.442
7.426
7.422
7.261
7.237
7.235
7.216
7.199
7.197
7.054
7.034
6.929
6.927
6.925
6.923
6.409
6.391
6.267
6.249
6.125
6.107
6.099
6.091
6.035
6.030
6.027
6.022
4.922
4.898
4.534
4.525
4.508
4.502
4.493
4.485
4.478
4.462
4.452
3.385
-0.000
CF2H
NH
3na
1H NMR (400 MHz, CDCl3)
1.09
5.02
1.05
0.96
0.99
0.95
1.01
1.00
0.94
1.00
1.00
0.98
f1 (ppm)

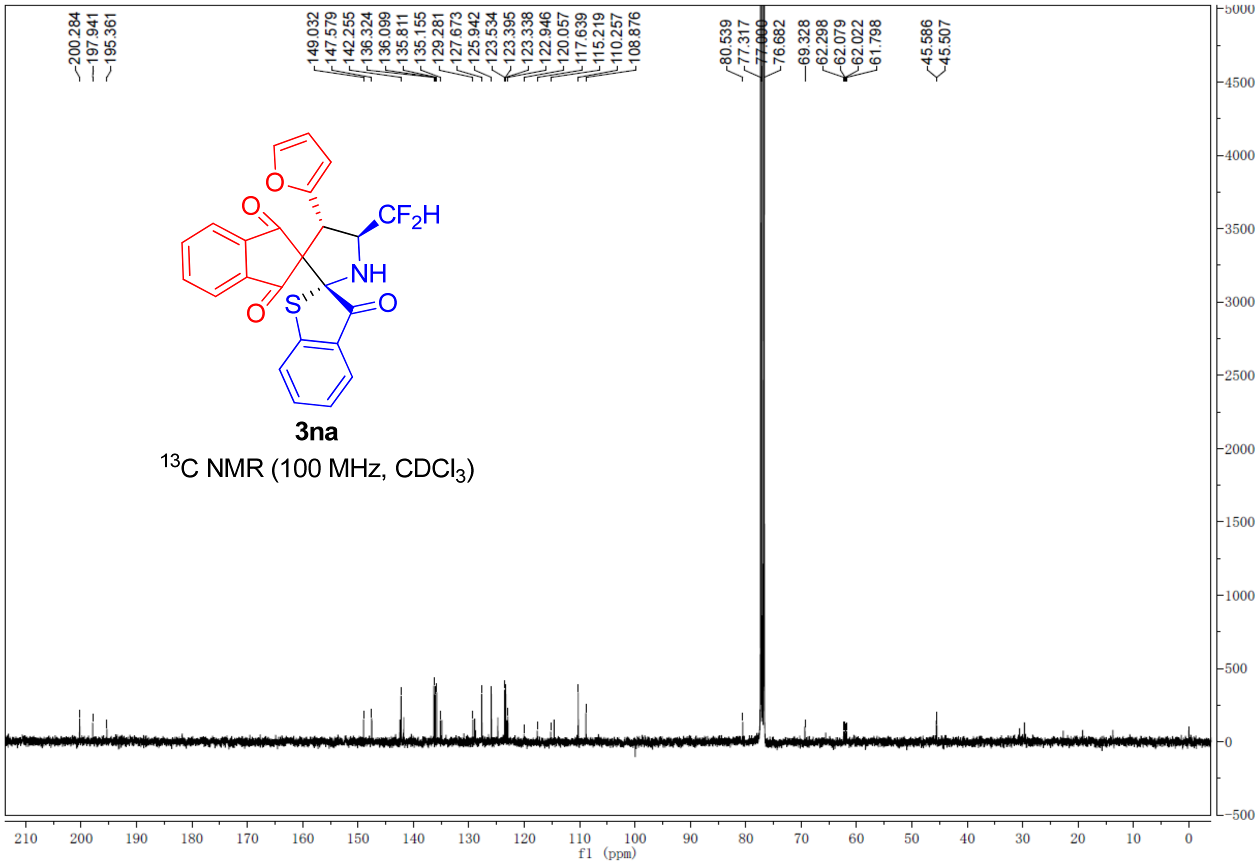
200.284
197.941
195.361
149.032
147.579
142.255
136.324
136.099
135.811
135.155
129.281
127.673
125.942
123.534
123.395
123.338
122.946
120.057
117.639
115.219
110.257
108.876
80.539
77.317
77.000
76.682
69.328
62.298
62.079
62.022
61.798
45.586
45.507
CF$_2$H
NH
O
S
3na
^{13}C NMR (100 MHz, CDCl$_3$)
f1 (ppm)

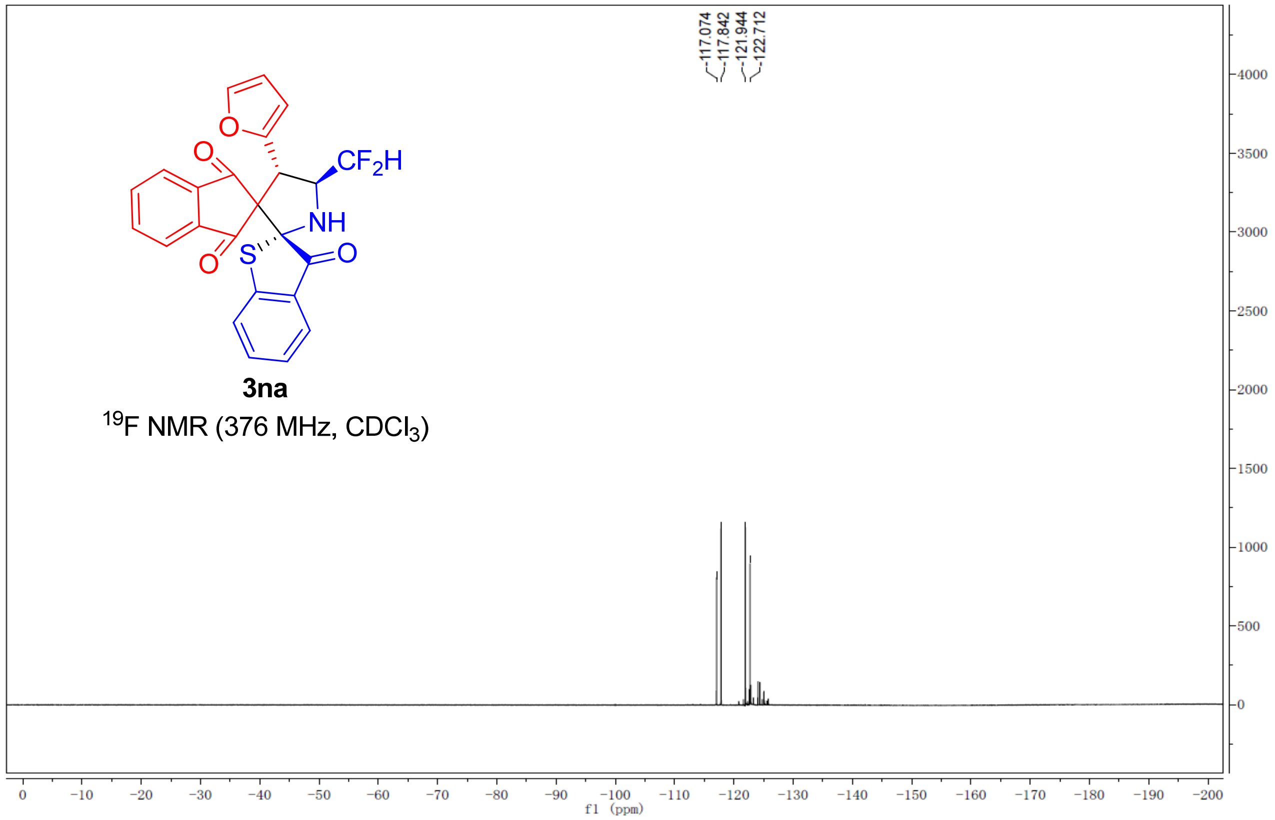
-117.074
-117.842
-121.944
-122.712
CF$_2$H
NH
O
S
3na
^{19}F NMR (376 MHz, CDCl$_3$)
f1 (ppm)

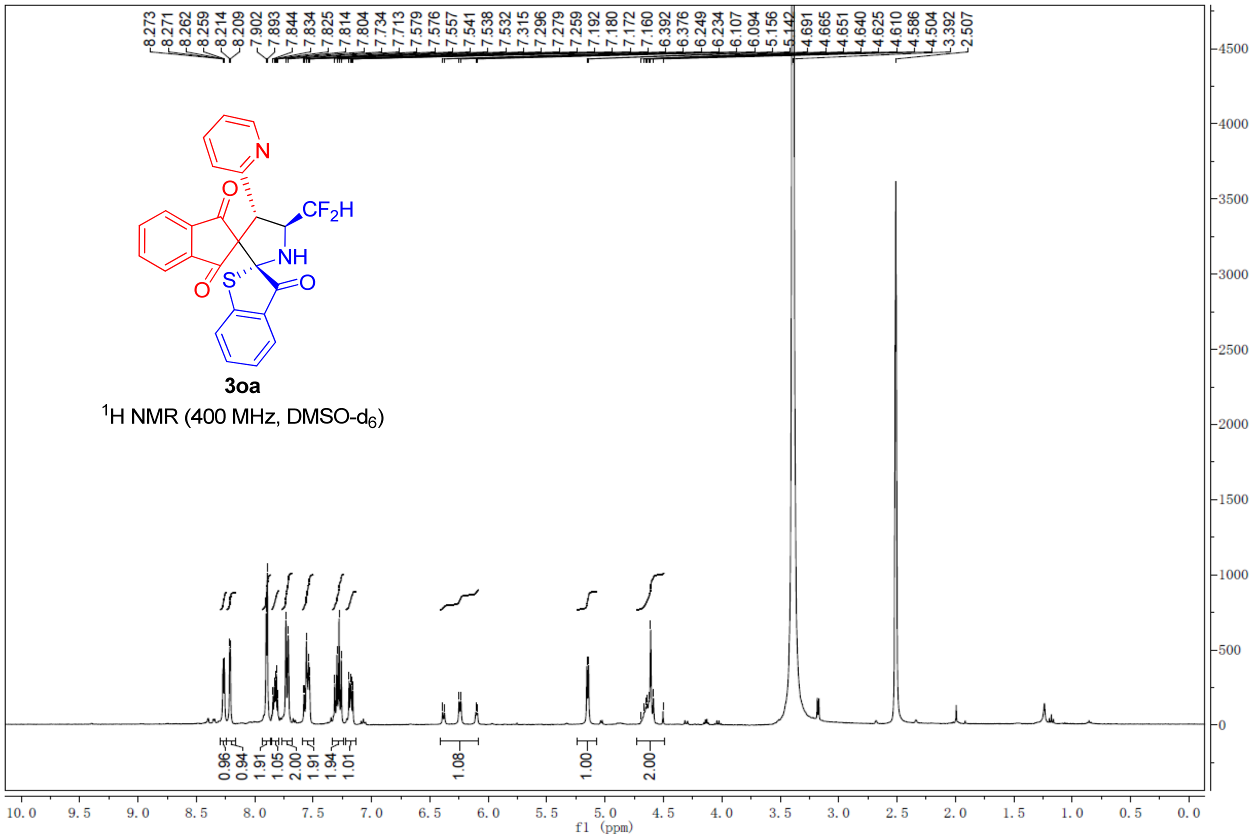
3oa
^{1}H NMR (400 MHz, DMSO-d$_6$)
f1 (ppm)

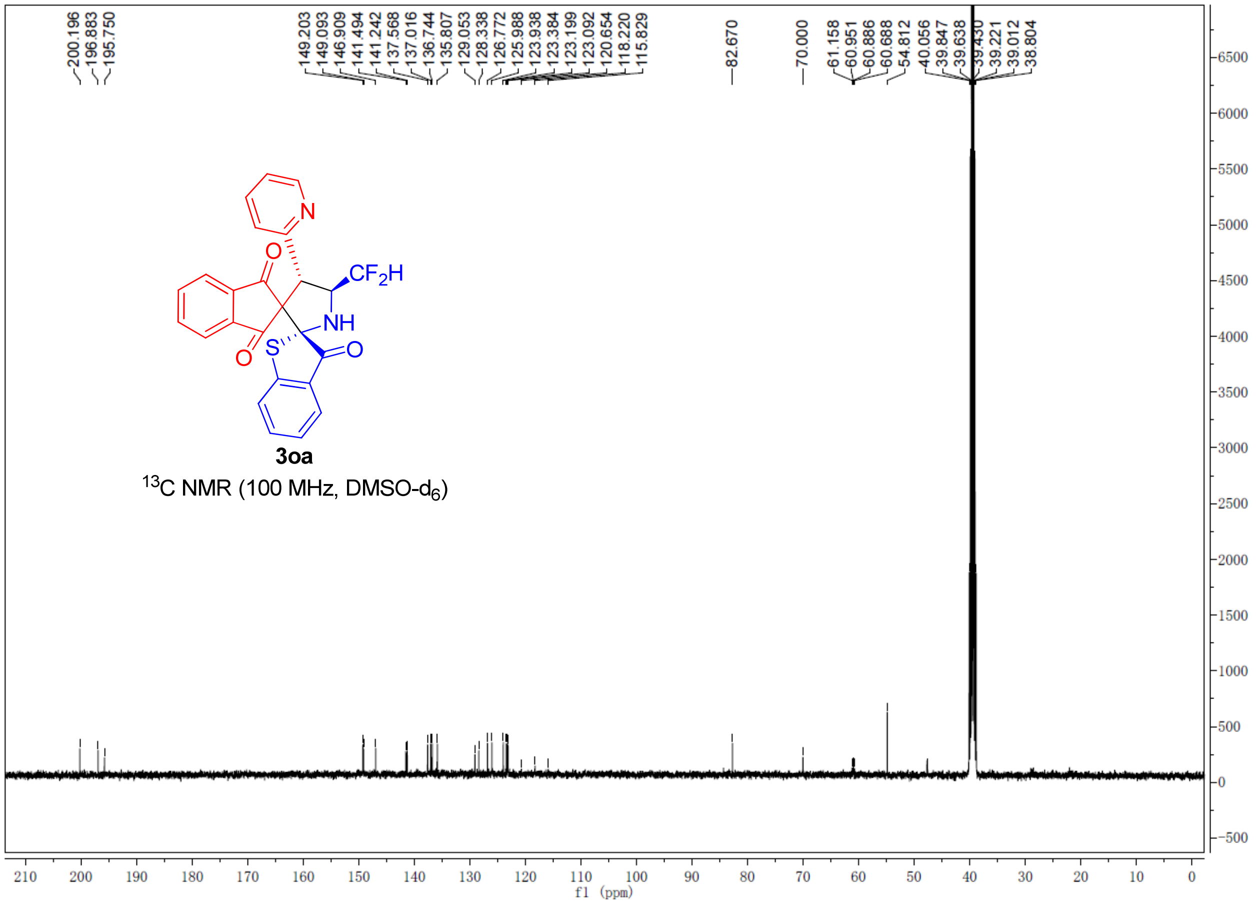
3oa
^{13}C NMR (100 MHz, DMSO-d$_6$)
f1 (ppm)

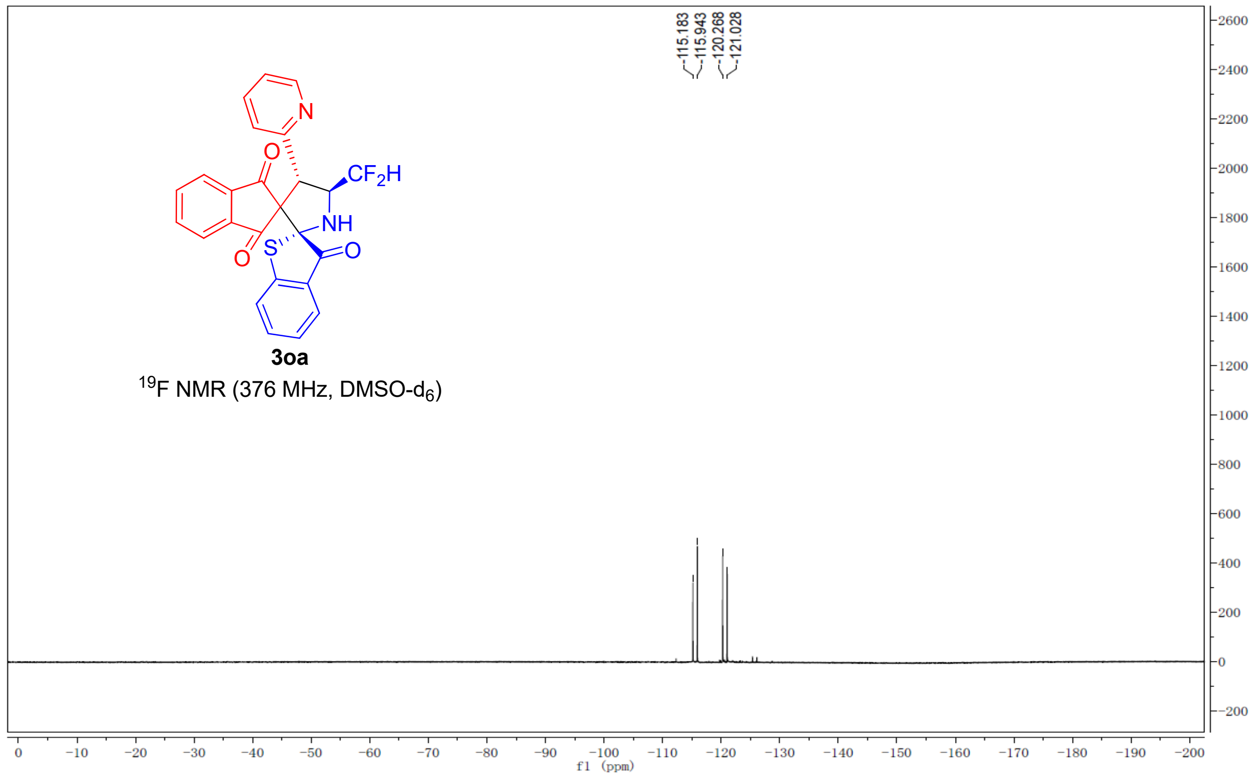

-115.183
-115.943
-120.268
-121.028
N
O
CF2H
NH
S
O
O
3oa
19F NMR (376 MHz, DMSO-d6)
f1 (ppm)

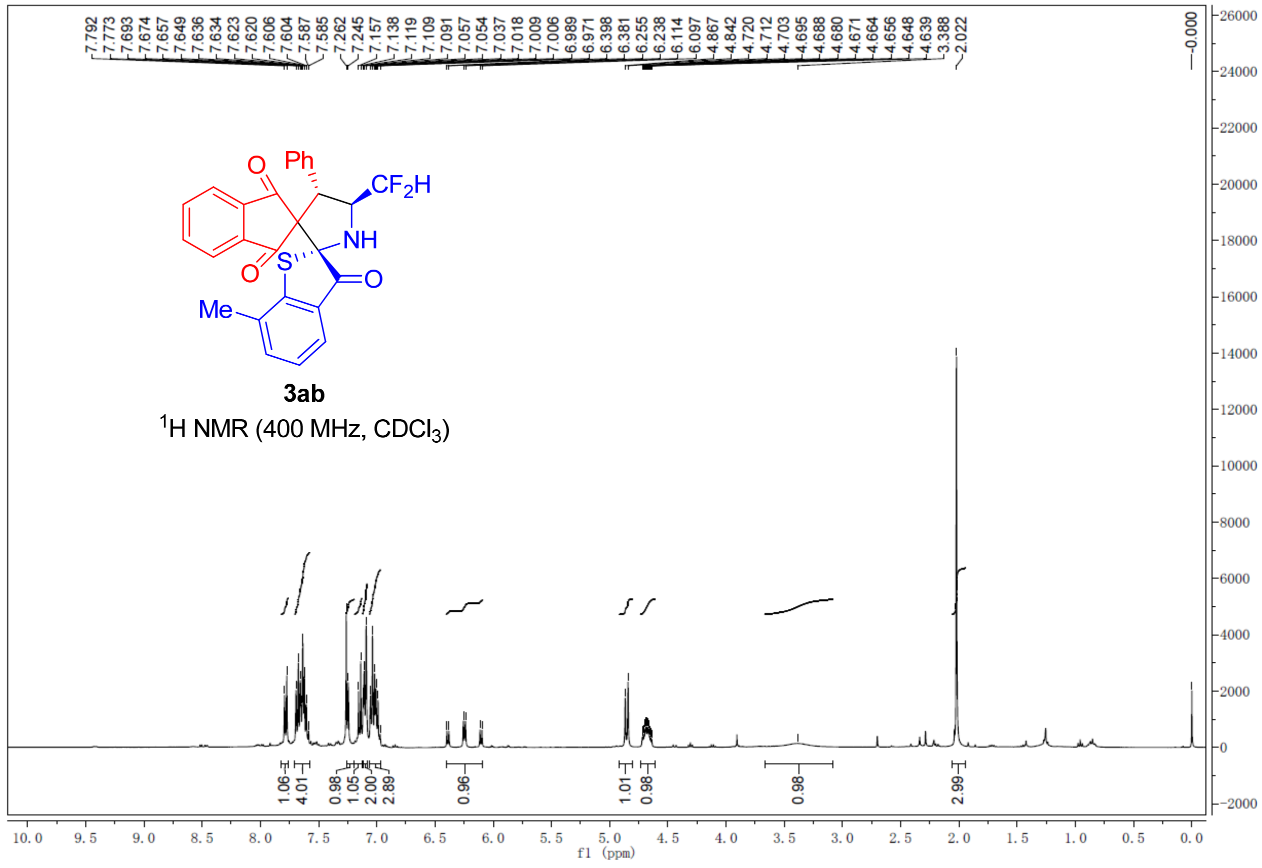

7.792
7.773
7.693
7.674
7.657
7.649
7.636
7.634
7.623
7.620
7.606
7.604
7.587
7.585
7.262
7.245
7.157
7.138
7.119
7.109
7.091
7.057
7.054
7.037
7.018
7.009
7.006
6.989
6.971
6.398
6.381
6.255
6.238
6.114
6.097
4.867
4.842
4.720
4.712
4.703
4.695
4.688
4.680
4.671
4.664
4.656
4.648
4.639
3.388
2.022
-0.000
O
Ph
CF2H
NH
S
O
O
Me
3ab
1H NMR (400 MHz, CDCl3)
1.06
4.01
0.98
1.05
2.00
2.89
0.96
1.01
0.98
0.98
2.99
f1 (ppm)

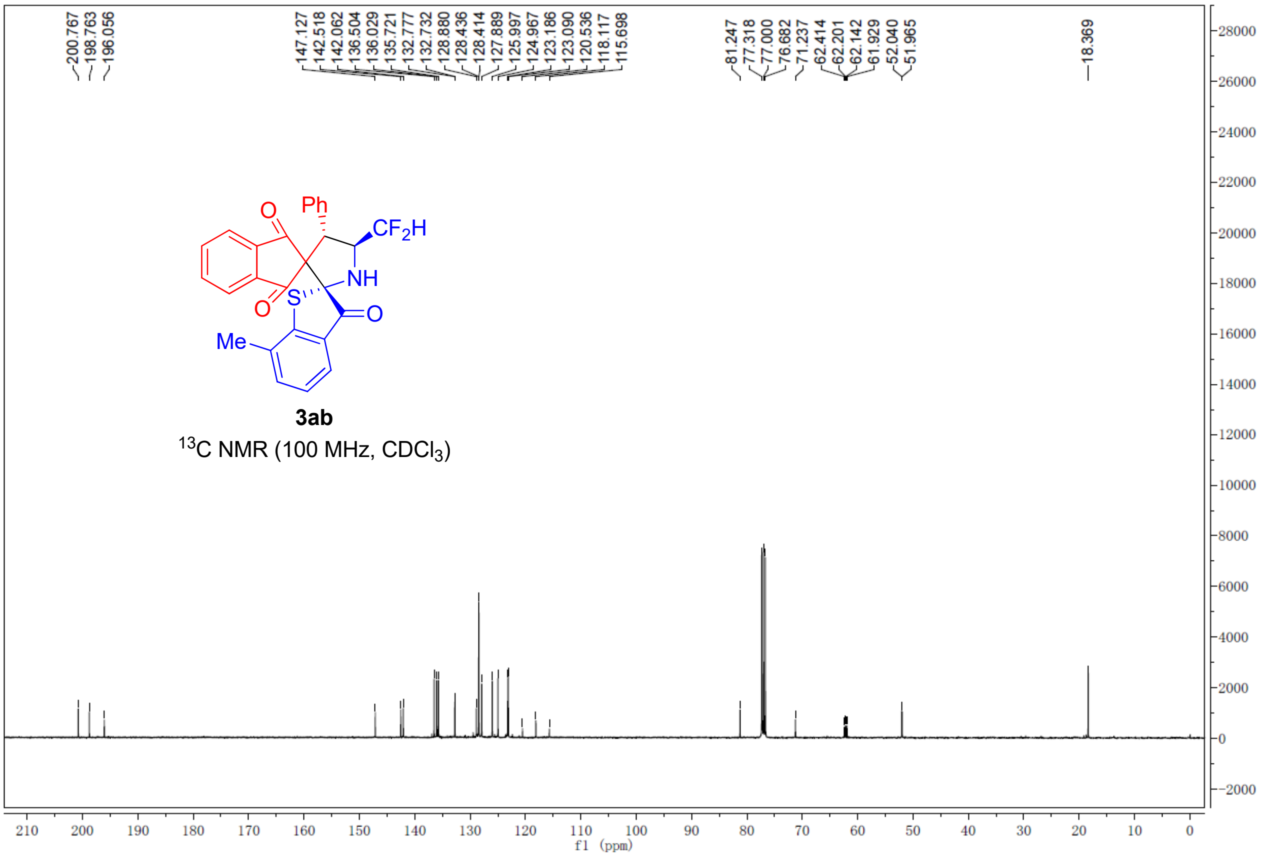

200.767
198.763
196.056
147.127
142.518
142.062
136.504
136.029
135.721
132.777
132.732
128.880
128.436
128.414
127.889
125.997
124.967
123.186
123.090
120.536
118.117
115.698
81.247
77.318
77.000
76.682
71.237
62.414
62.201
62.142
61.929
52.040
51.965
18.369
Ph
CF2H
NH
S
Me
O
3ab
13C NMR (100 MHz, CDCl3)
f1 (ppm)

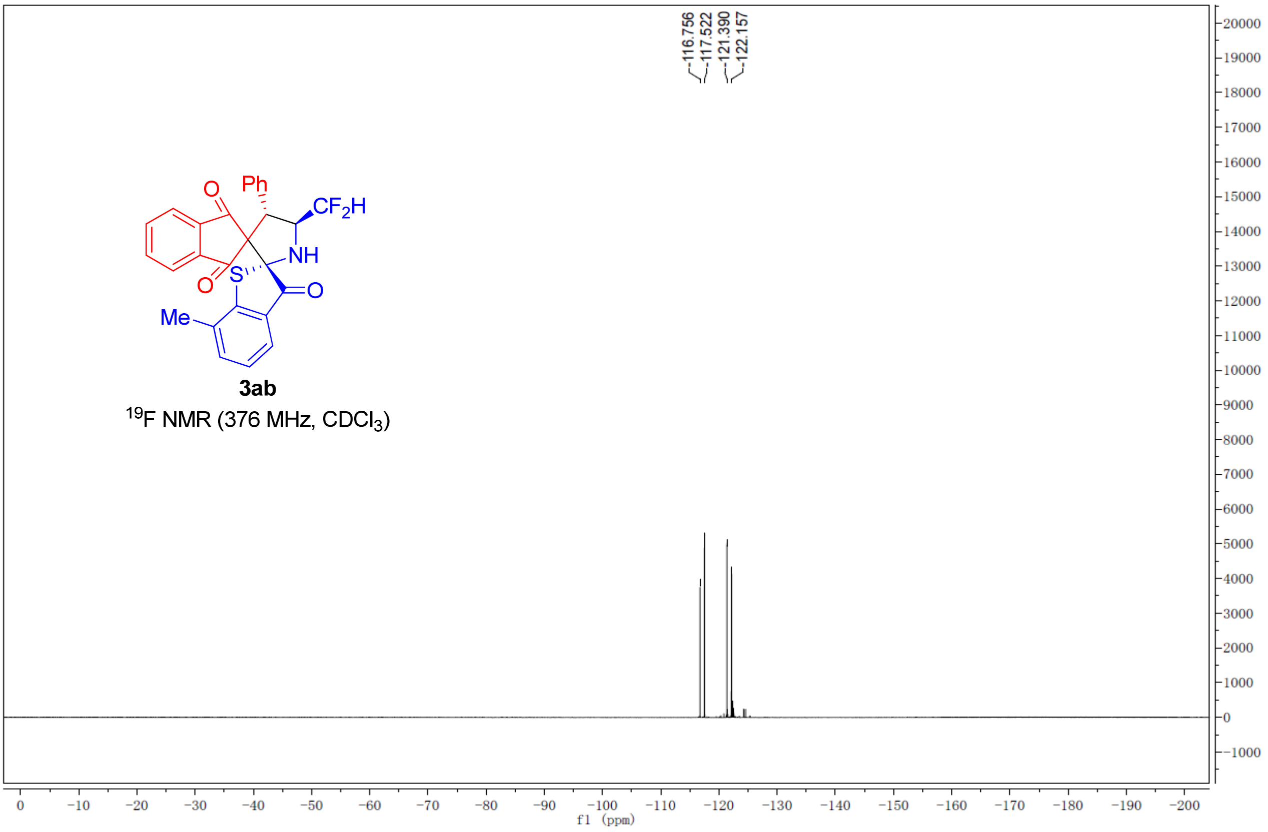

-116.756
-117.522
-121.390
-122.157
Ph
CF2H
NH
S
Me
O
3ab
19F NMR (376 MHz, CDCl3)
f1 (ppm)

2. Methodology for determining the dr

The dr was determined using NMR spectra. We chose the proton in CF_2H group, this proton has been split into td peaks owing to coupling with F atoms. Comparison the same integration areas of major diastereomer with minor diastereomer can obtain the dr value. For example, using **3aa** and **3ca**, the corresponding integrations as following:

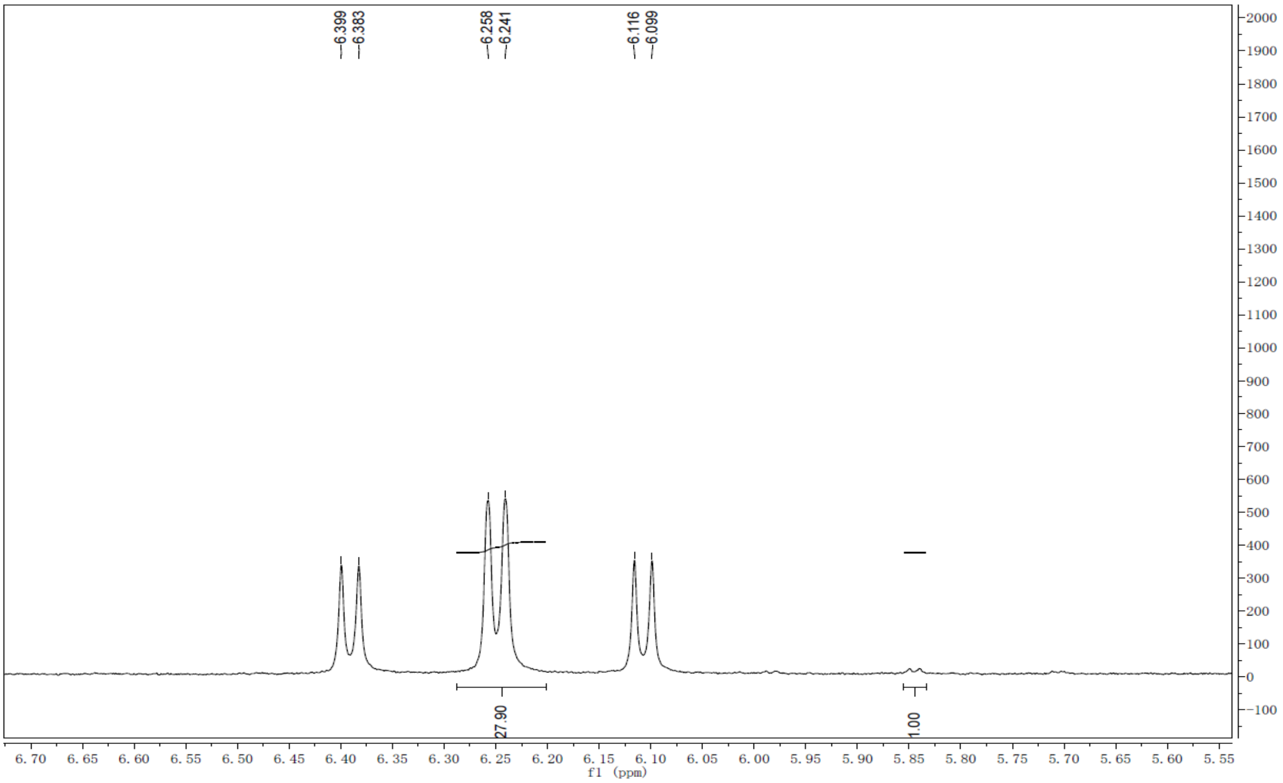

3aa: dr = 28:1, i.e. >20:1

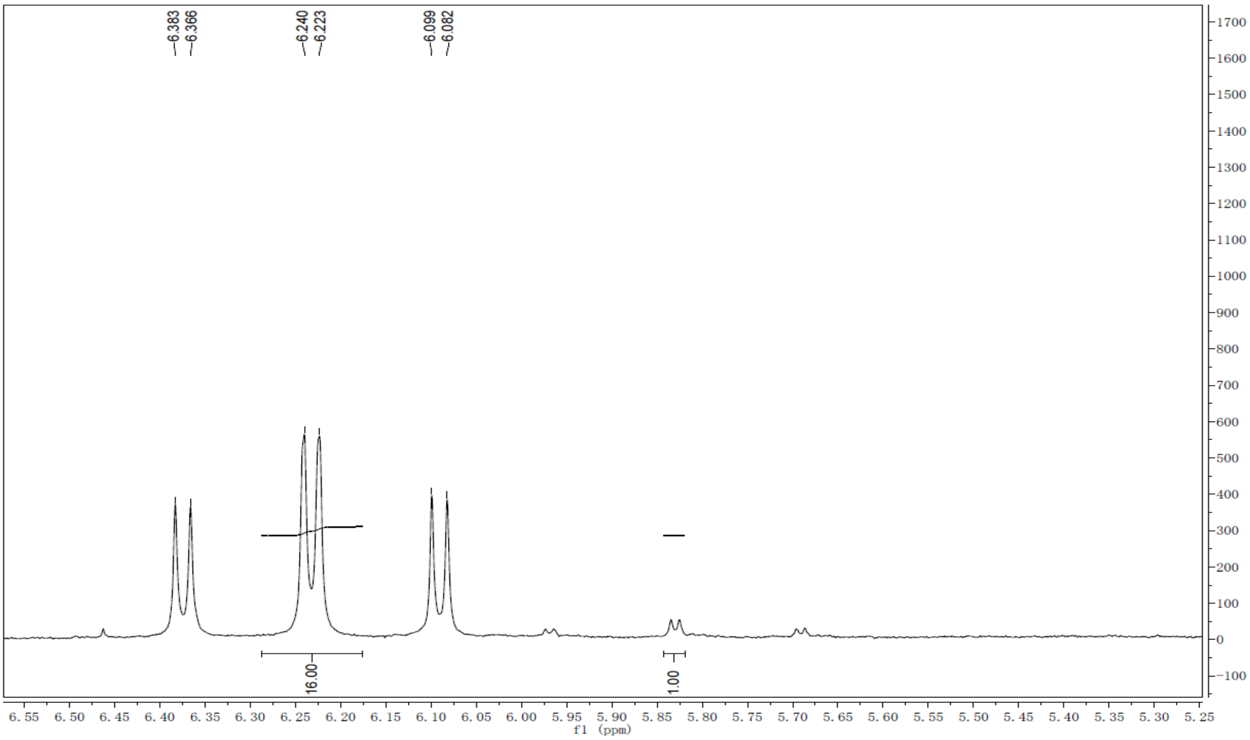

3ca: dr = 16:1

3. X-ray single-crystal data for product 3aa

The single crystal of **3aa** was cultured by slow evaporation in ethyl acetate at room temperature, the single crystal data see Table S1.

Table S1 Crystal data and structure refinement for **3aa**

Identification code	CCDC 2349208	
Empirical formula	C26H17F2NO3S	
Formula weight	461.46	
Temperature	296.15 K	
Wavelength	1.54184 Å	
Crystal system	monoclinic	
Space group	$P2_12_12_1$	
Unit cell dimensions	a = 7.43144(8) Å	α= 90°.
	b = 18.0016(3) Å	β= 98.0891(10) °.
	c = 16.52414(16) Å	γ = 90°.
Volume	2188.57(5) $Å^3$	
Z	4	
Density (calculated)	1.401 Mg/m^3	
Absorption coefficient	1.718 mm^{-1}	
F(000)	952	
Crystal size	0.22 × 0.20 × 0.15 mm^3	
Theta range for data collection	3.651 to 76.323°.	
Index ranges	$-7 \leqslant h \leqslant 9, -20 \leqslant k \leqslant 22, -20 \leqslant l \leqslant 20$	
Reflections collected	21270	
Independent reflections	4411 [Rint = 0.0352, Rsigma = 0.0265]	
Data / restraints / parameters	4411/0/298	
Goodness-of-fit on F^2	1.053	
Final R indices [I>2sigma(I)]	R1 = 0.0429, wR2 = 0.1196	
R indices (all data)	R1 = 0.0479, wR2 = 0.1243	
Largest diff. peak and hole	0.45 and -0.52 e.$Å^{-3}$	

4. Copies of HPLC chromatograms

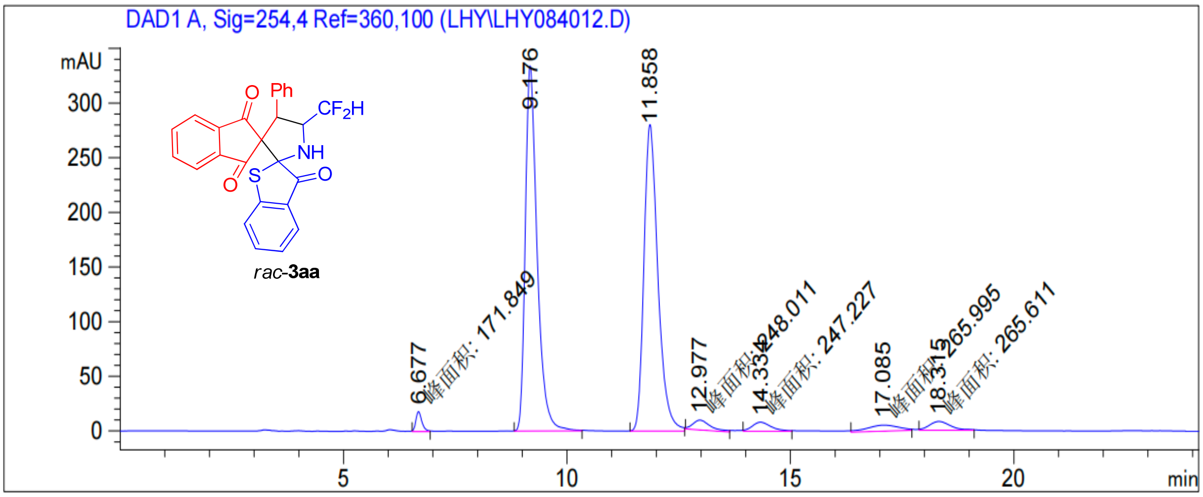

Peak #	RetTime [min]	Type	Width [min]	Area [mAU*s]	Height [mAU]	Area %
1	6.677	MM	0.1566	171.84943	18.29082	1.2785
2	9.176	BB	0.2781	6130.92529	334.14597	45.6124
3	11.858	BV	0.3323	6111.72363	280.41525	45.4696
4	12.977	MM	0.4601	248.01126	8.98408	1.8451
5	14.334	MM	0.4986	247.22690	8.26398	1.8393
6	17.085	MM	0.8139	265.99509	5.44682	1.9789
7	18.315	MM	0.5527	265.61118	8.00945	1.9761

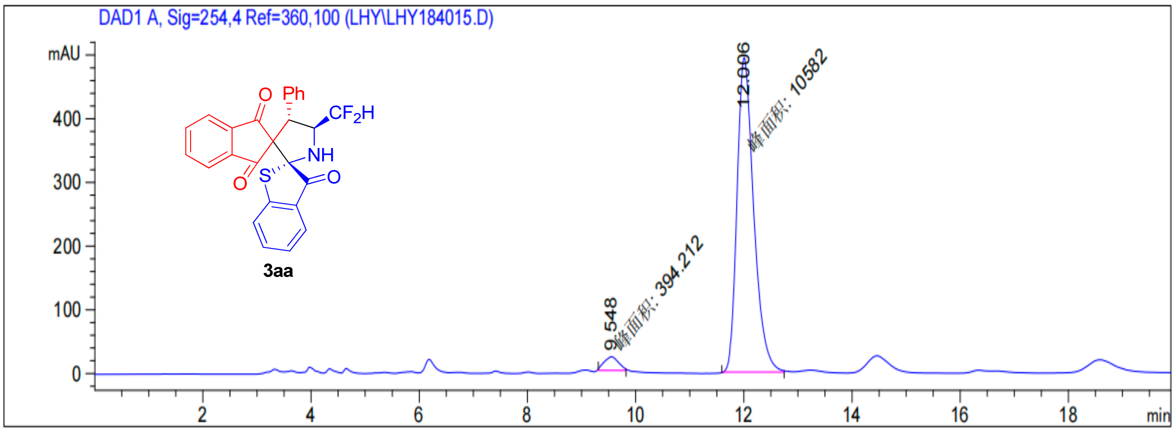

Peak #	RetTime [min]	Type	Width [min]	Area [mAU*s]	Height [mAU]	Area %
1	9.548	MM	0.3074	394.21173	21.37284	3.5915
2	12.006	MM	0.3571	1.05820e4	493.86740	96.4085

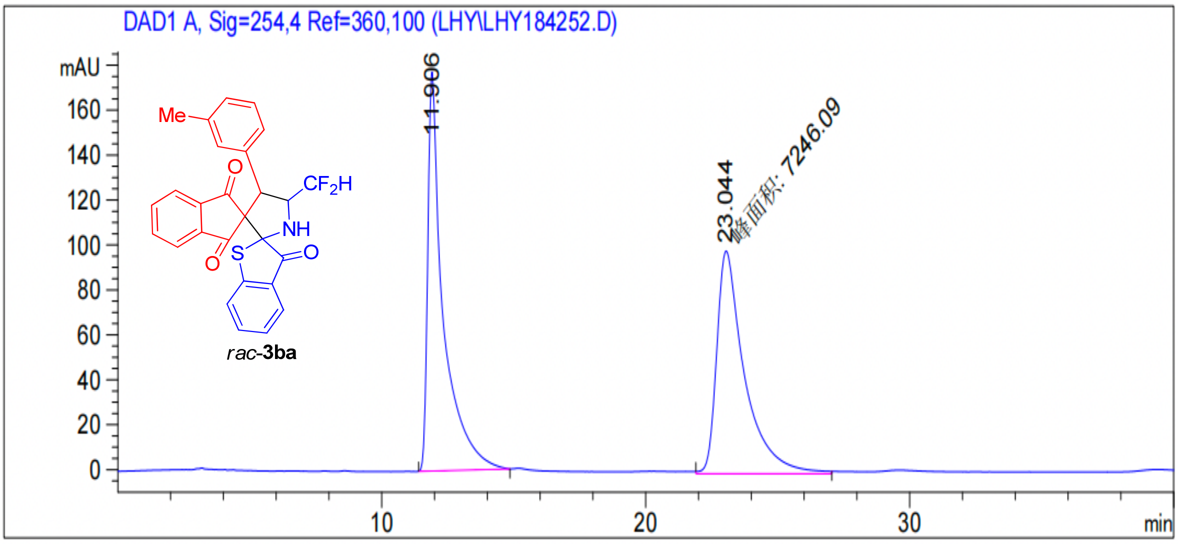

Peak #	RetTime [min]	Type	Width [min]	Area [mAU*s]	Height [mAU]	Area %
1	11.906	BB	0.5609	7229.11328	177.60425	49.9413
2	23.044	MM	1.2192	7246.09277	99.05511	50.0587

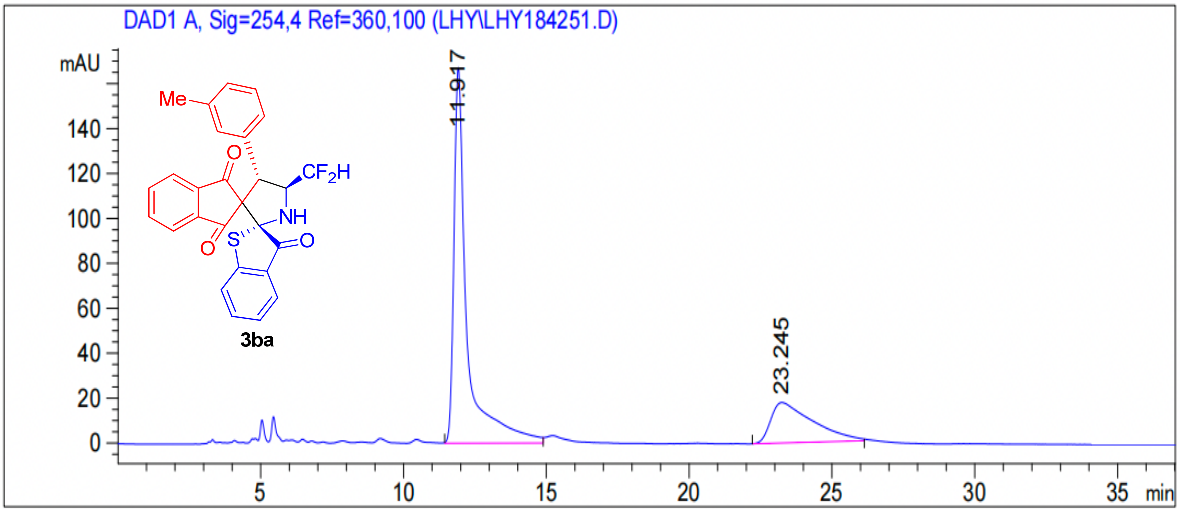

Peak #	RetTime [min]	Type	Width [min]	Area [mAU*s]	Height [mAU]	Area %
1	11.917	BB	0.4501	5277.97949	167.27501	74.2035
2	23.245	BB	1.3522	1834.86853	18.04649	25.7965

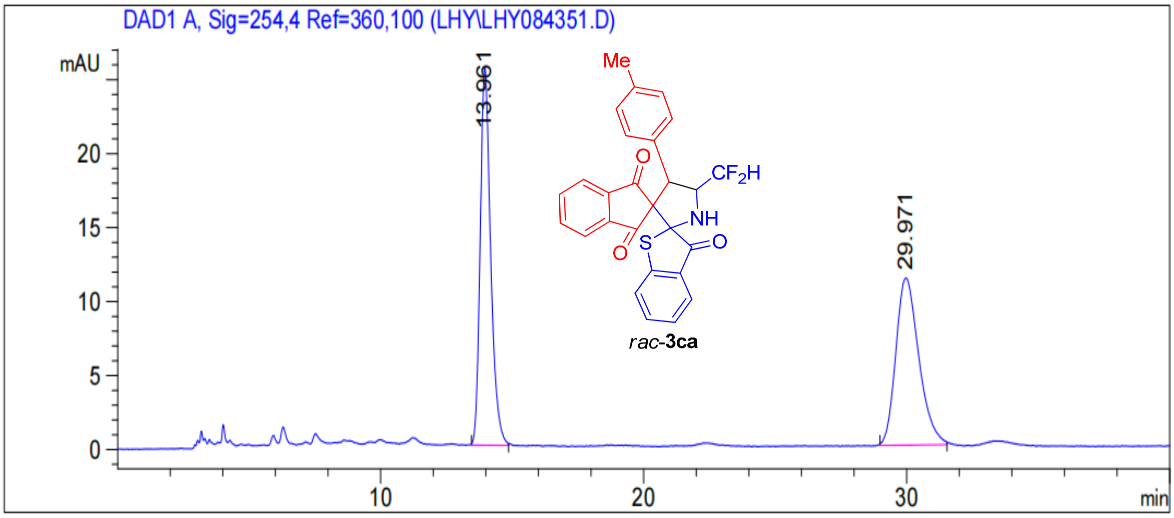

Peak #	RetTime [min]	Type	Width [min]	Area [mAU*s]	Height [mAU]	Area %
1	13.961	BB	0.4223	704.06323	25.48721	50.6217
2	29.971	BB	0.8973	686.76904	11.29612	49.3783

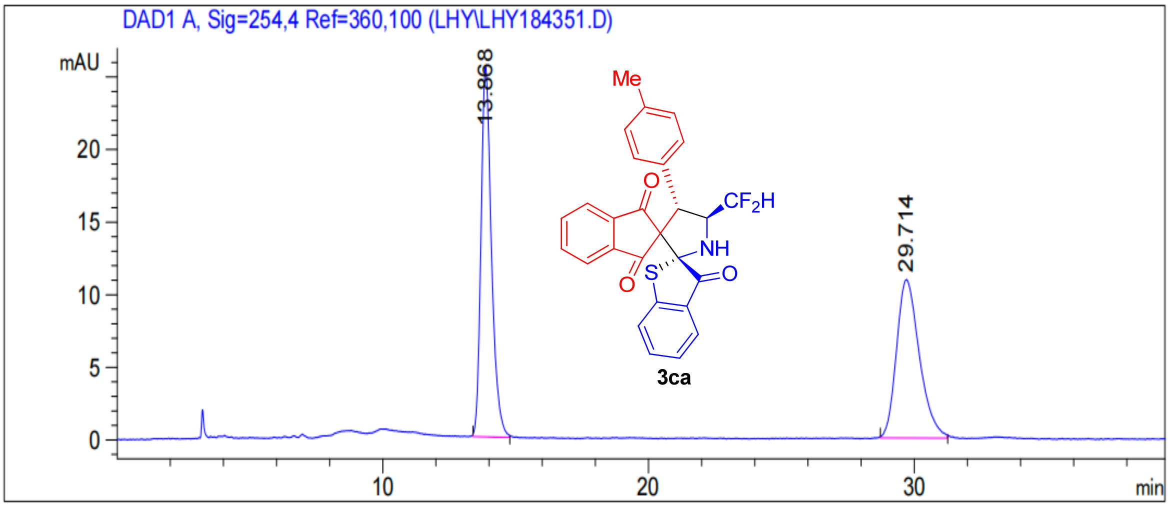

Peak #	RetTime [min]	Type	Width [min]	Area [mAU*s]	Height [mAU]	Area %
1	13.868	BB	0.4142	694.92047	25.47519	51.5980
2	29.714	BB	0.8330	651.87769	10.91182	48.4020

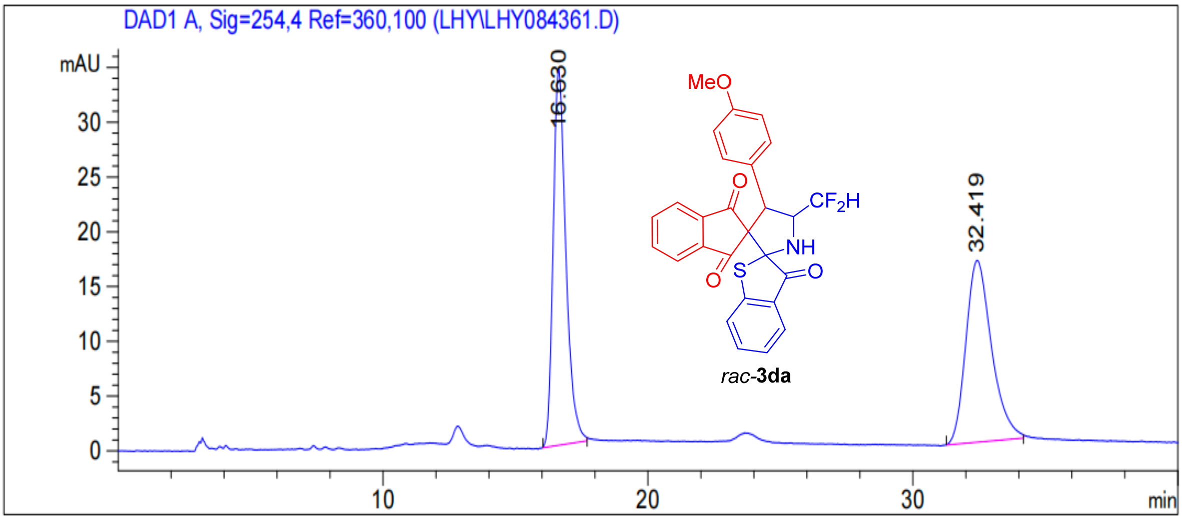

Peak #	RetTime [min]	Type	Width [min]	Area [mAU*s]	Height [mAU]	Area %
1	16.630	BB	0.5056	1142.65588	34.39807	50.5843
2	32.419	BB	0.9695	1116.25940	16.57994	49.4157

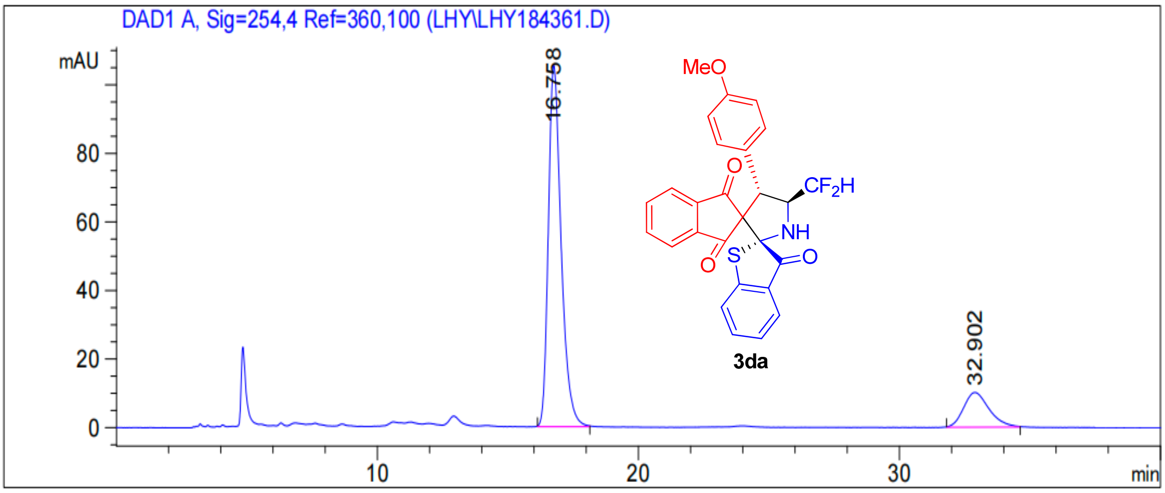

Peak #	RetTime [min]	Type	Width [min]	Area [mAU*s]	Height [mAU]	Area %
1	16.758	BB	0.5180	3577.86475	105.40877	83.8502
2	32.902	BB	0.8979	689.10541	10.10088	16.1498

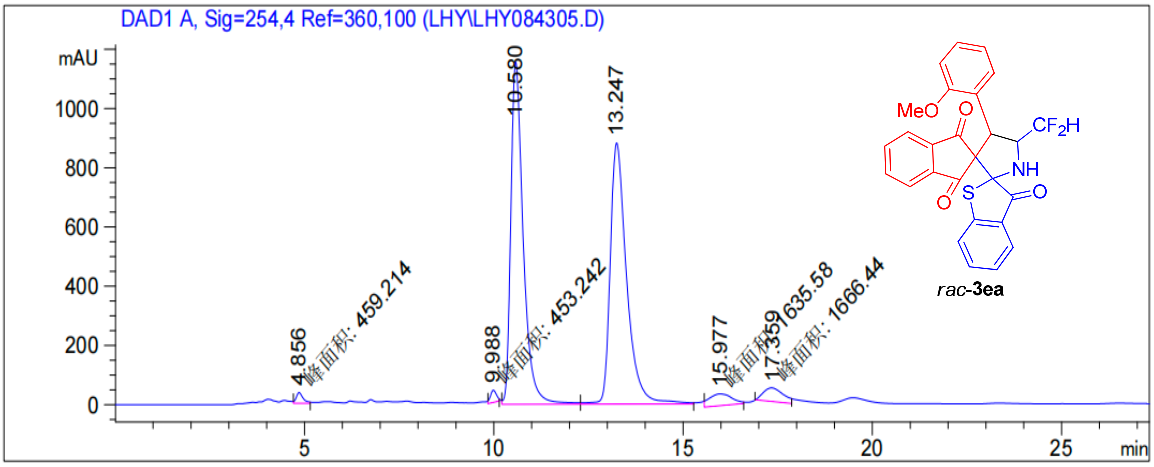

Peak #	RetTime [min]	Type	Width [min]	Area [mAU*s]	Height [mAU]	Area %
1	4.856	MM	0.2105	459.21387	36.36203	0.8030
2	9.988	MM	0.1824	453.24203	41.40450	0.7925
3	10.580	VV	0.3456	2.63008e4	1155.11279	45.9879
4	13.247	VV	0.4531	2.66754e4	881.45844	46.6429

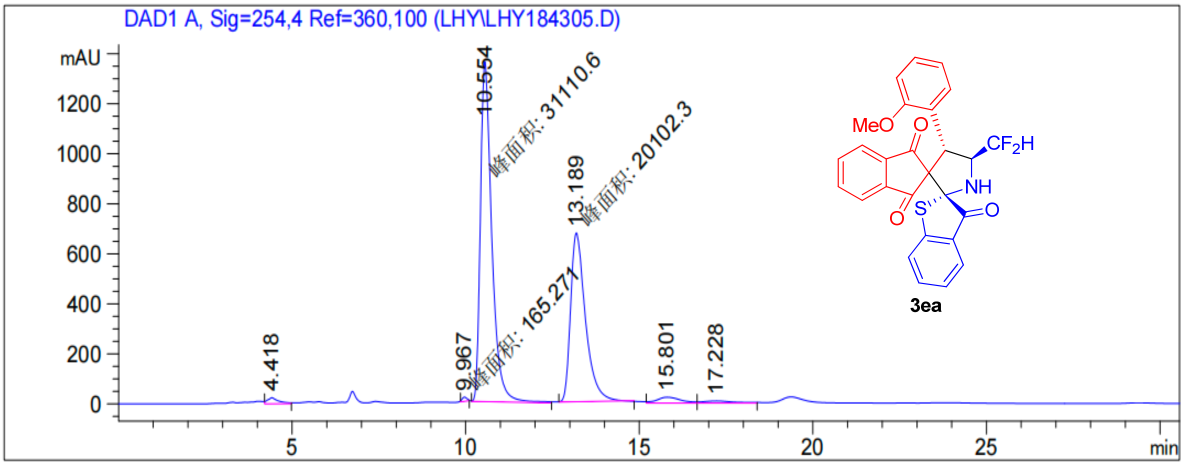

Peak #	RetTime [min]	Type	Width [min]	Area [mAU*s]	Height [mAU]	Area %
1	4.418	VV	0.2959	547.83801	24.22311	1.0215
2	9.967	MM	0.1630	165.27052	16.90235	0.3082
3	10.554	MM	0.3812	3.11106e4	1360.22571	58.0092
4	13.189	MM	0.4963	2.01023e4	675.11859	37.4830

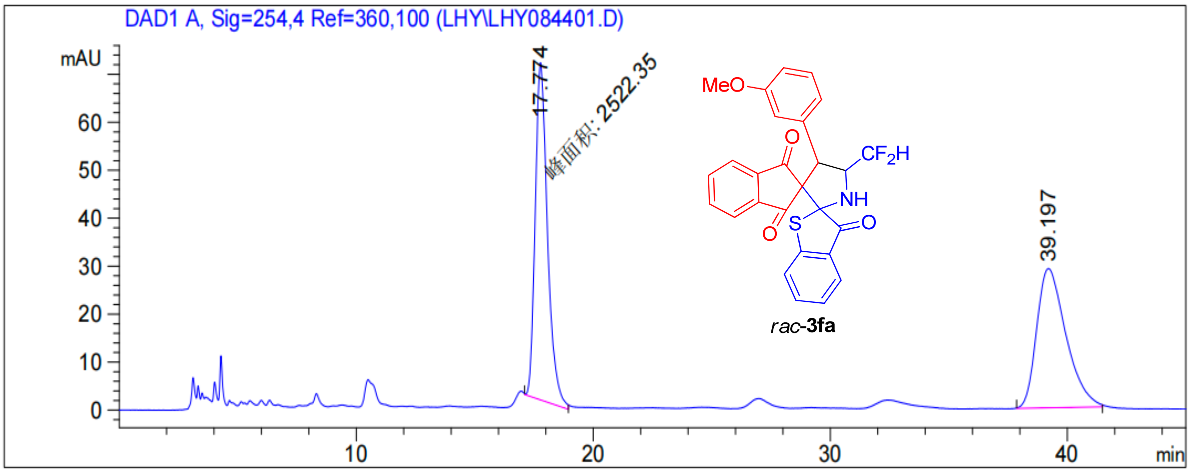

Peak #	RetTime [min]	Type	Width [min]	Area [mAU*s]	Height [mAU]	Area %
1	17.774	MM	0.5979	2522.34521	70.30589	50.3762
2	39.197	BB	1.2209	2484.66919	29.02044	49.6238

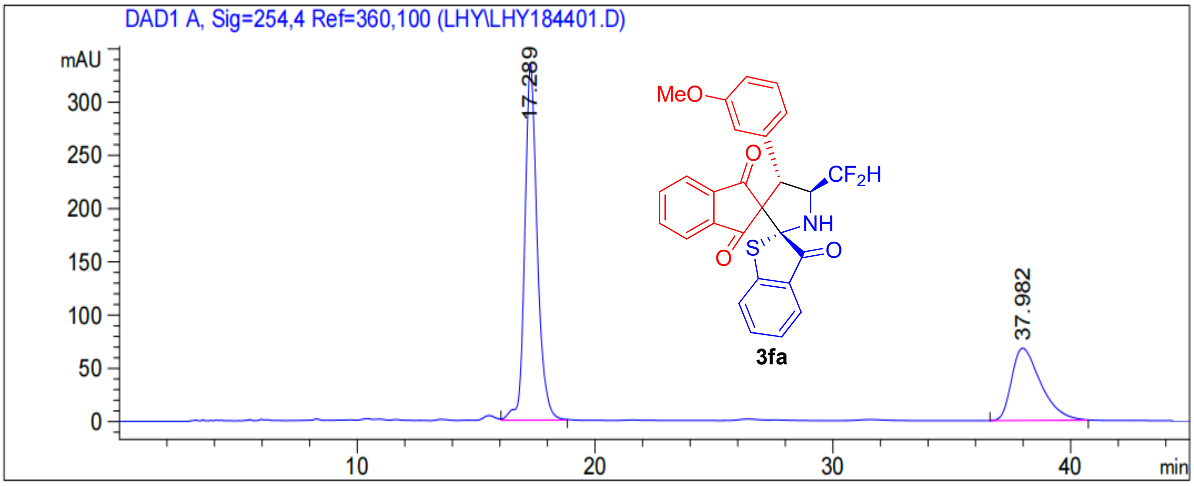

Peak #	RetTime [min]	Type	Width [min]	Area [mAU*s]	Height [mAU]	Area %
1	17.289	VB	0.5499	1.22063e4	334.36835	67.7927
2	37.982	BB	1.2680	5799.02588	67.92553	32.2073

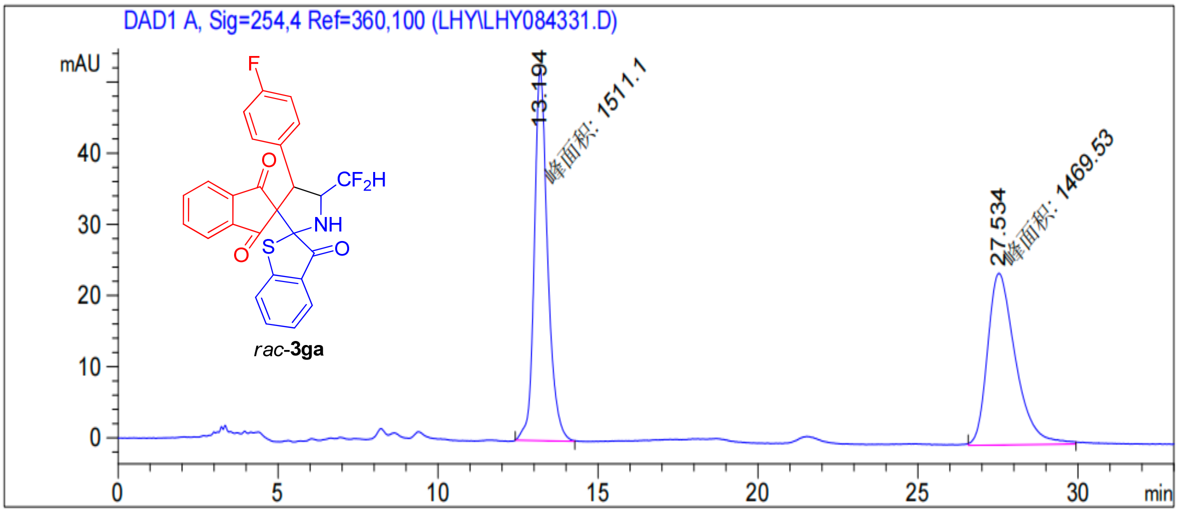

Peak #	RetTime [min]	Type	Width [min]	Area [mAU*s]	Height [mAU]	Area %
1	13.194	MM	0.4806	1511.10461	52.40659	50.6974
2	27.534	MM	1.0148	1469.52795	24.13529	49.3026

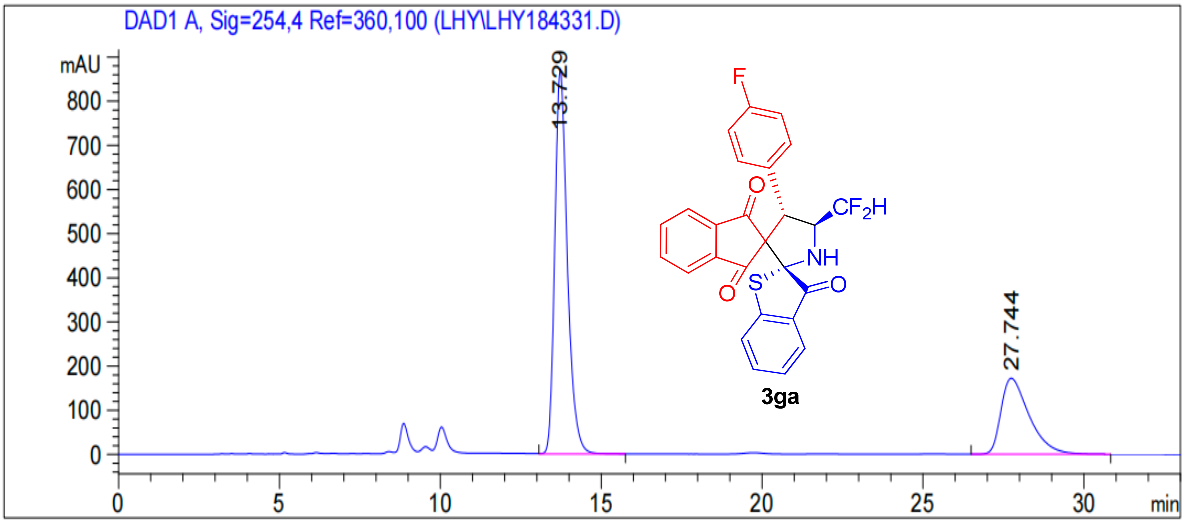

Peak #	RetTime [min]	Type	Width [min]	Area [mAU*s]	Height [mAU]	Area %
1	13.729	VB	0.4156	2.35893e4	872.08044	69.1963
2	27.744	VB	0.9324	1.05011e4	171.96779	30.8037

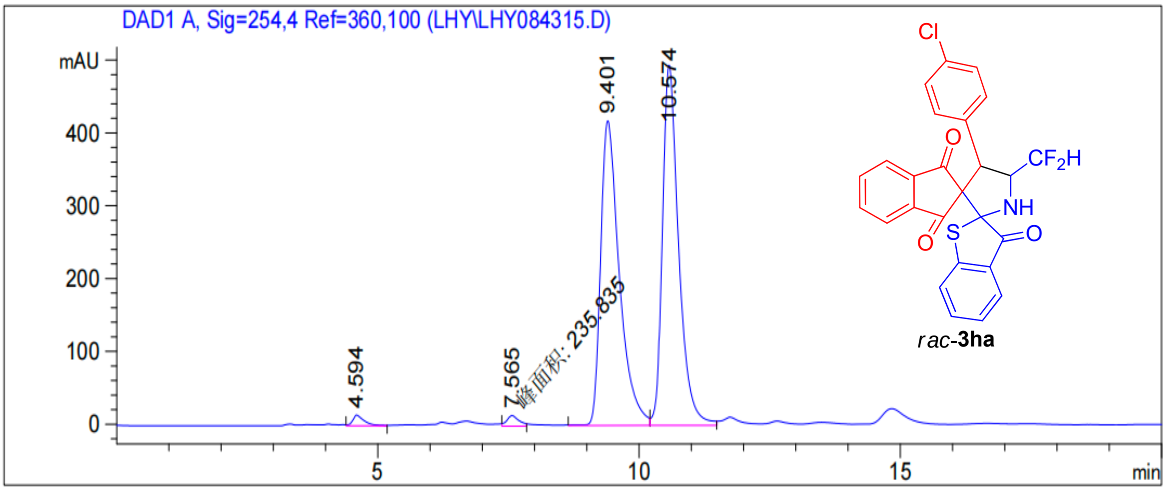

Peak #	RetTime [min]	Type	Width [min]	Area [mAU*s]	Height [mAU]	Area %
1	4.594	VB	0.2084	221.99506	14.68158	1.0306
2	7.565	MM	0.2684	235.83522	14.64229	1.0948
3	9.401	BV	0.3746	1.04192e4	418.30914	48.3689
4	10.574	VV	0.3236	1.06641e4	494.45865	49.5057

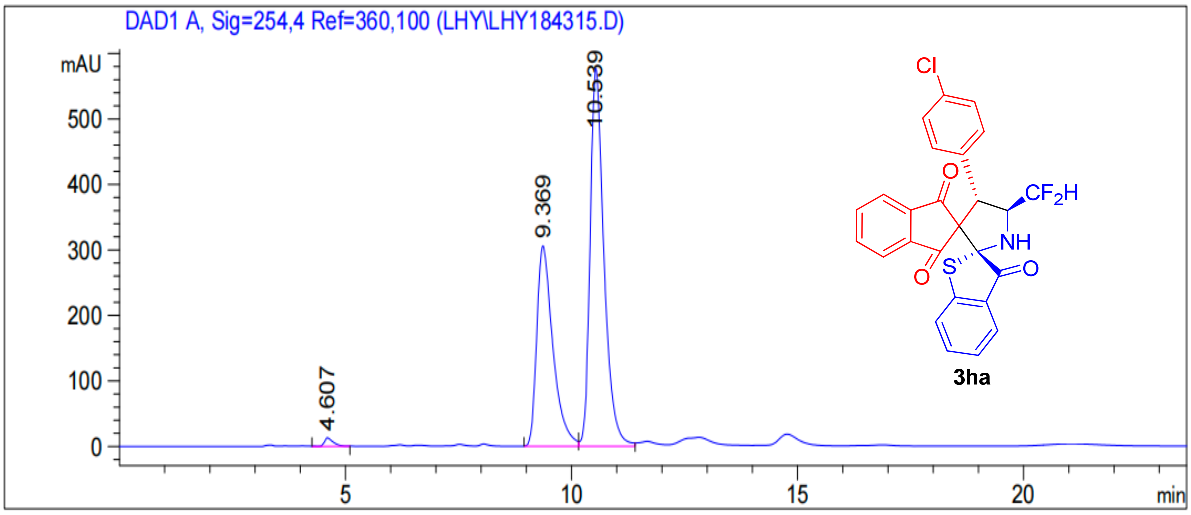

Peak #	RetTime [min]	Type	Width [min]	Area [mAU*s]	Height [mAU]	Area %
1	4.607	VB	0.1994	180.35374	13.19756	0.8971
2	9.369	BV	0.3741	7612.37891	306.13309	37.8637
3	10.539	VV	0.3250	1.23119e4	576.88251	61.2392

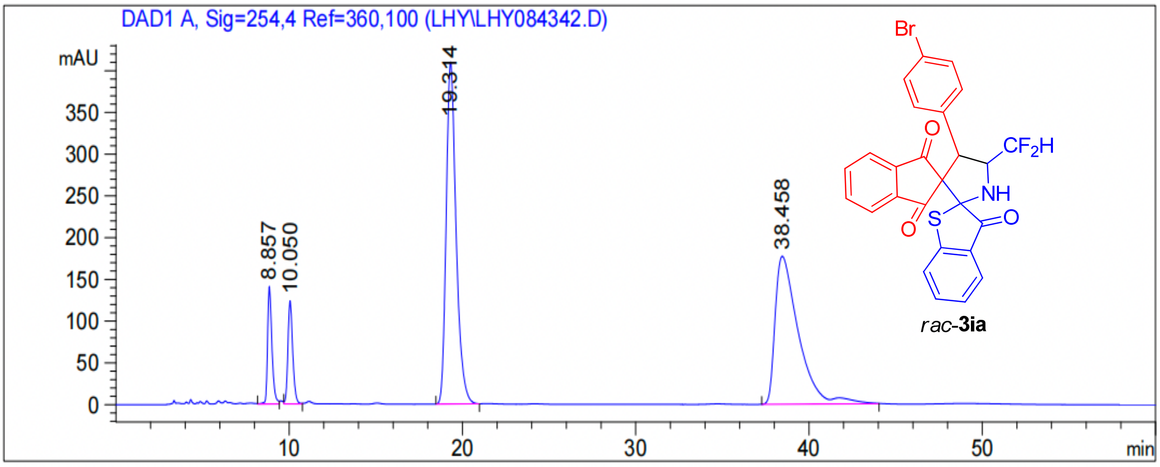

Peak #	RetTime [min]	Type	Width [min]	Area [mAU*s]	Height [mAU]	Area %
1	8.857	VV	0.2643	2470.61792	141.05635	6.5498
2	10.050	VB	0.2958	2392.97070	123.62904	6.3440
3	19.314	BB	0.6024	1.62393e4	409.70554	43.0518
4	38.458	BB R	1.3974	1.66175e4	177.34041	44.0544

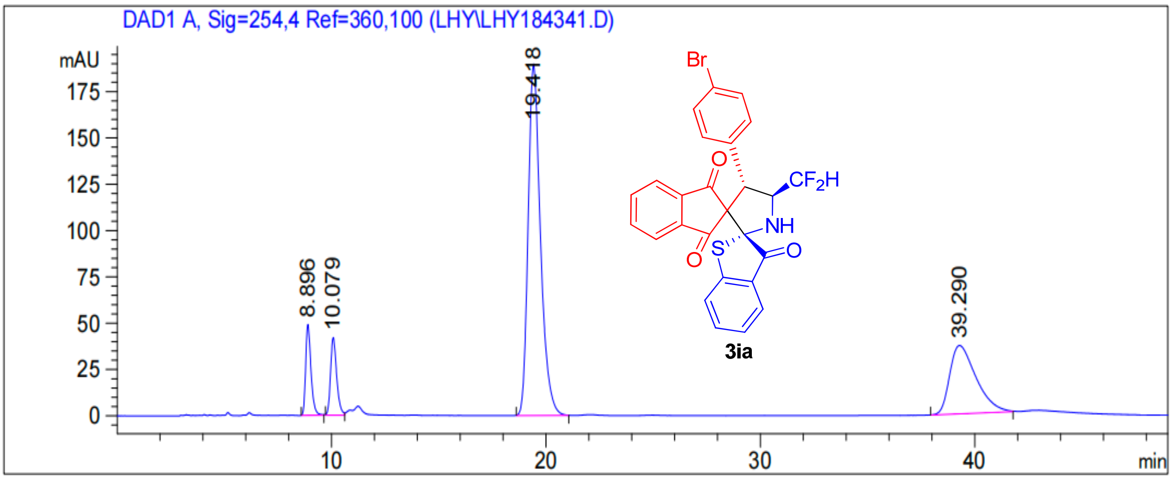

Peak #	RetTime [min]	Type	Width [min]	Area [mAU*s]	Height [mAU]	Area %
1	8.896	BB	0.2609	845.63904	49.10434	6.8180
2	10.079	BV	0.3000	828.42114	42.01851	6.6792
3	19.418	BB	0.6080	7581.04883	189.80109	61.1225
4	39.290	BB	1.1916	3147.93872	36.88812	25.3804

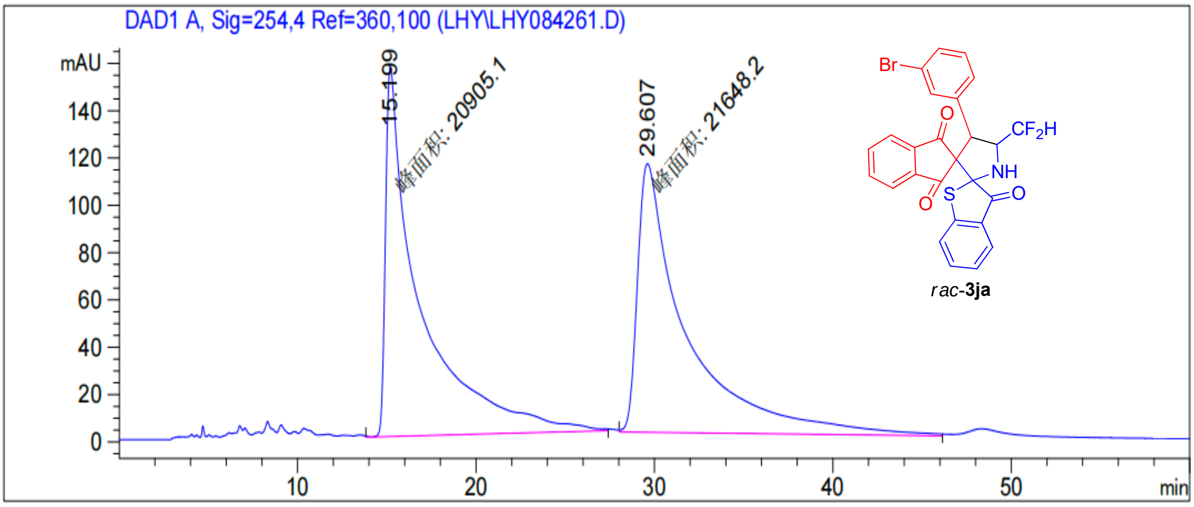

Peak #	RetTime [min]	Type	Width [min]	Area [mAU*s]	Height [mAU]	Area %
1	15.199	MM	2.2298	2.09051e4	156.25247	49.1269
2	29.607	MM	3.1770	2.16482e4	113.56737	50.8731

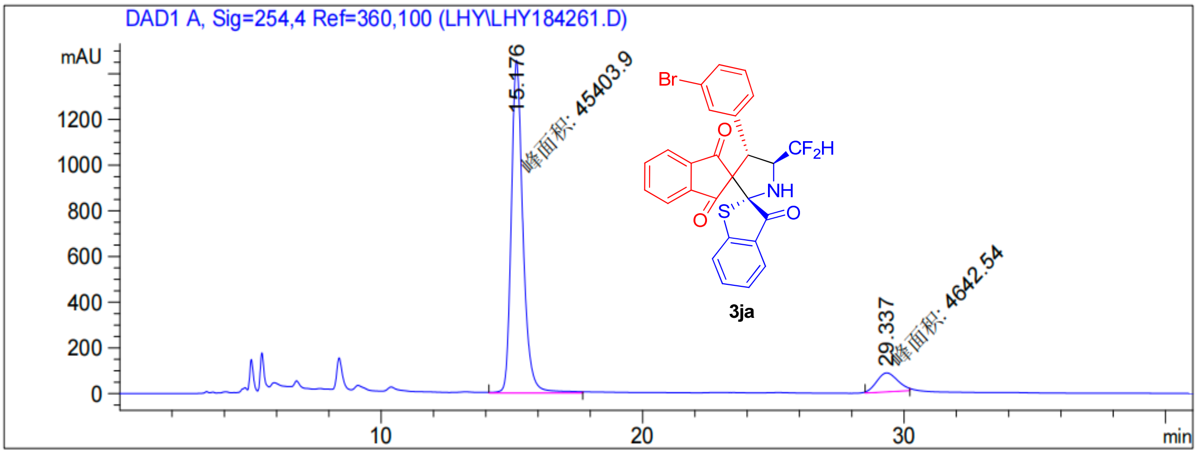

Peak #	RetTime [min]	Type	Width [min]	Area [mAU*s]	Height [mAU]	Area %
1	15.176	MM	0.5196	4.54039e4	1456.46191	90.7235
2	29.337	MM	0.9317	4642.54199	83.05104	9.2765

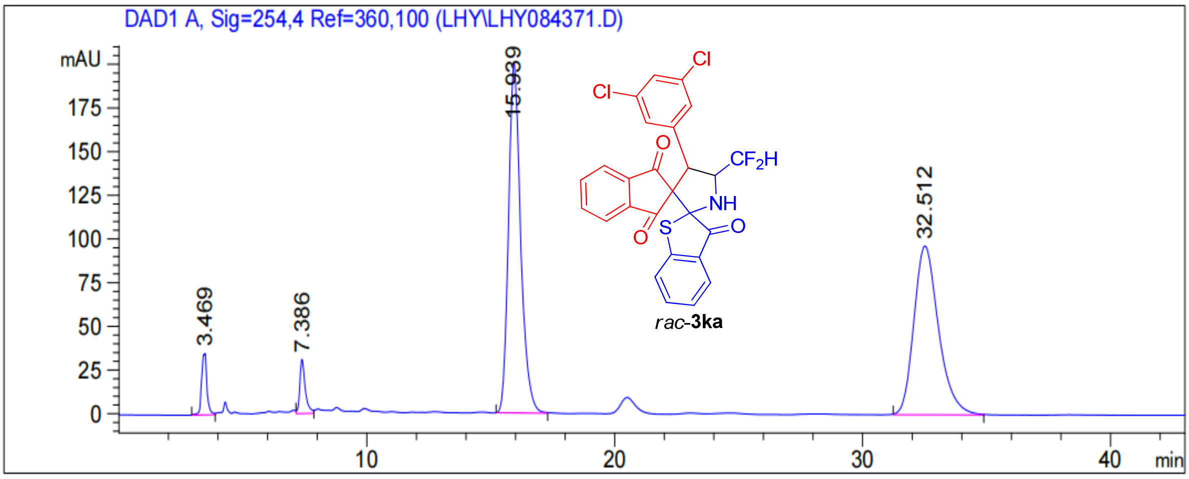

Peak #	RetTime [min]	Type	Width [min]	Area [mAU*s]	Height [mAU]	Area %
1	3.469	BV	0.1935	523.44019	35.19275	3.6102
2	7.386	VV	0.2258	471.84802	30.95982	3.2544
3	15.939	BB	0.5183	6830.39600	200.04640	47.1100
4	32.512	BB	1.0539	6673.14209	96.59996	46.0254

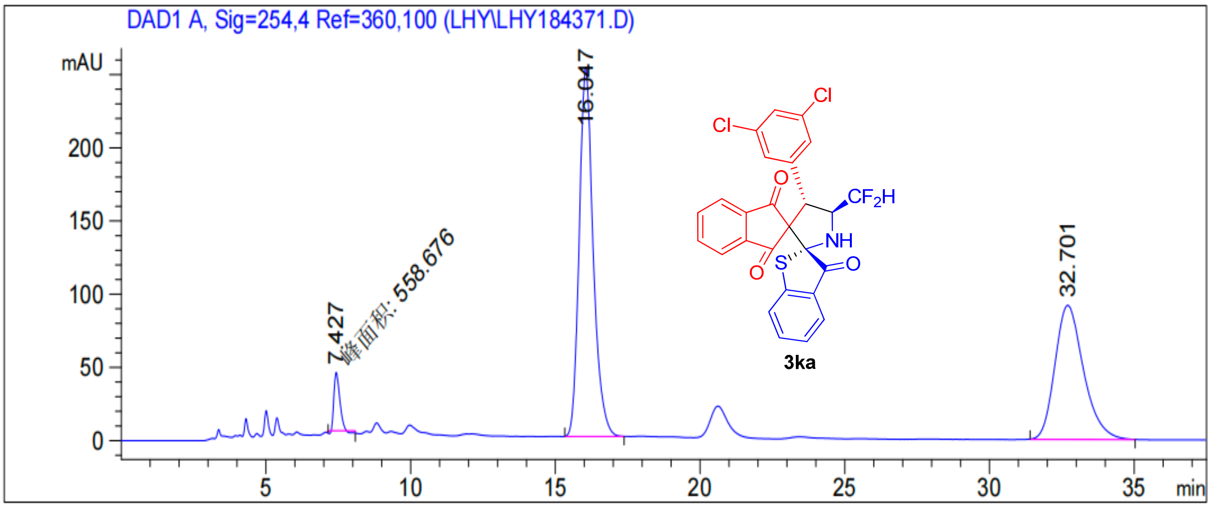

Peak #	RetTime [min]	Type	Width [min]	Area [mAU*s]	Height [mAU]	Area %
1	7.427	MM	0.2338	558.67639	39.83327	3.6404
2	16.047	BB	0.5113	8462.35059	252.28004	55.1415
3	32.701	BB	1.0446	6325.59619	91.71661	41.2182

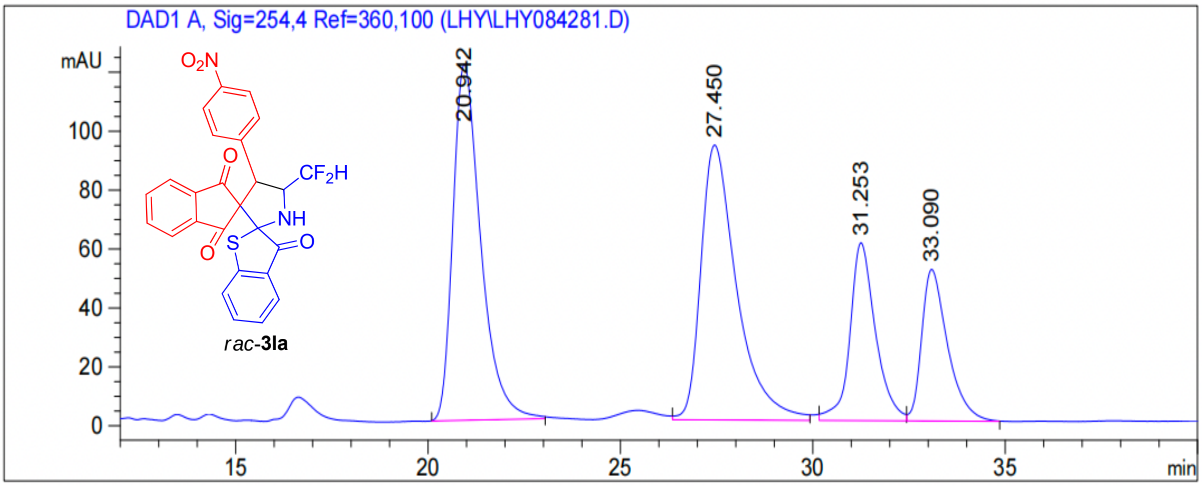

Peak #	RetTime [min]	Type	Width [min]	Area [mAU*s]	Height [mAU]	Area %
1	20.942	BB	0.7625	6069.64551	120.73341	35.0282
2	27.450	VB	0.9732	6055.58496	93.30805	34.9471
3	31.253	BV	0.6871	2789.95093	60.37413	16.1009
4	33.090	VB	0.7112	2412.70093	51.44323	13.9238

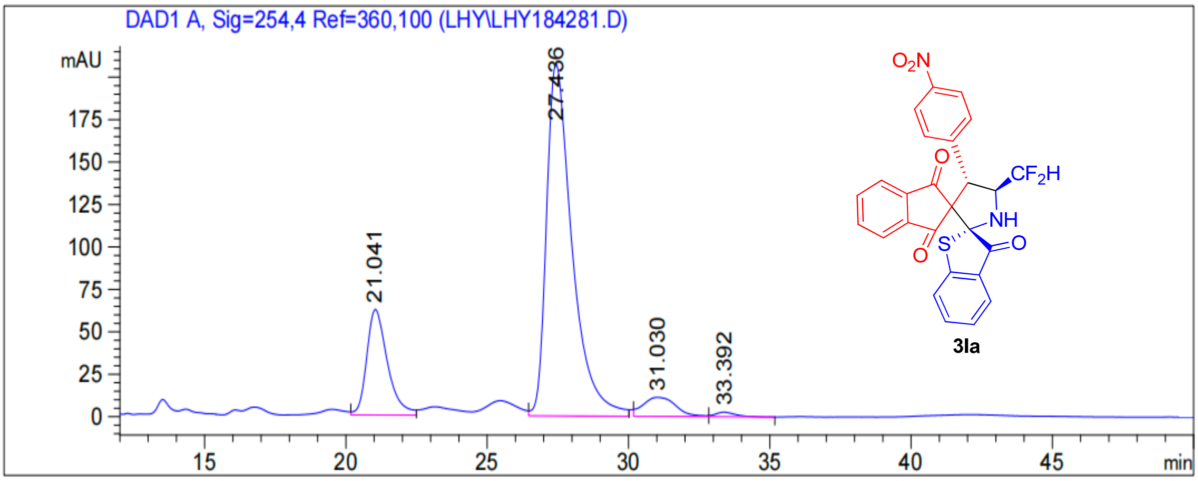

Peak #	RetTime [min]	Type	Width [min]	Area [mAU*s]	Height [mAU]	Area %
1	21.041	VB	0.7794	3173.93652	62.19172	18.3480
2	27.436	VB	0.9476	1.30726e4	207.33345	75.5705
3	31.030	BB	0.9716	917.70038	11.25084	5.3051
4	33.392	BB	0.6328	134.32051	2.75491	0.7765

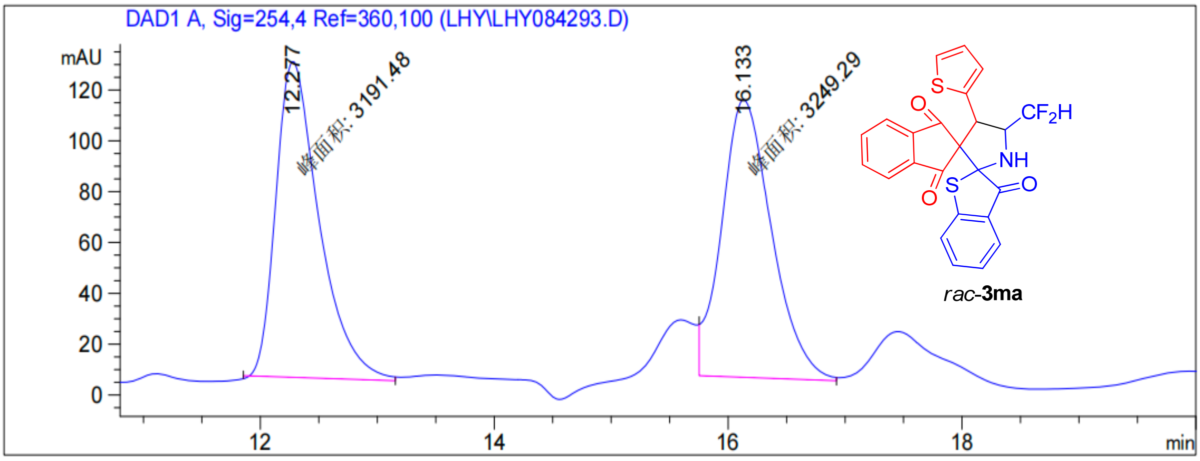

Peak #	RetTime [min]	Type	Width [min]	Area [mAU*s]	Height [mAU]	Area %
1	12.277	MM	0.4277	3191.48022	124.37860	49.5512
2	16.133	MM	0.4955	3249.29272	109.29931	50.4488

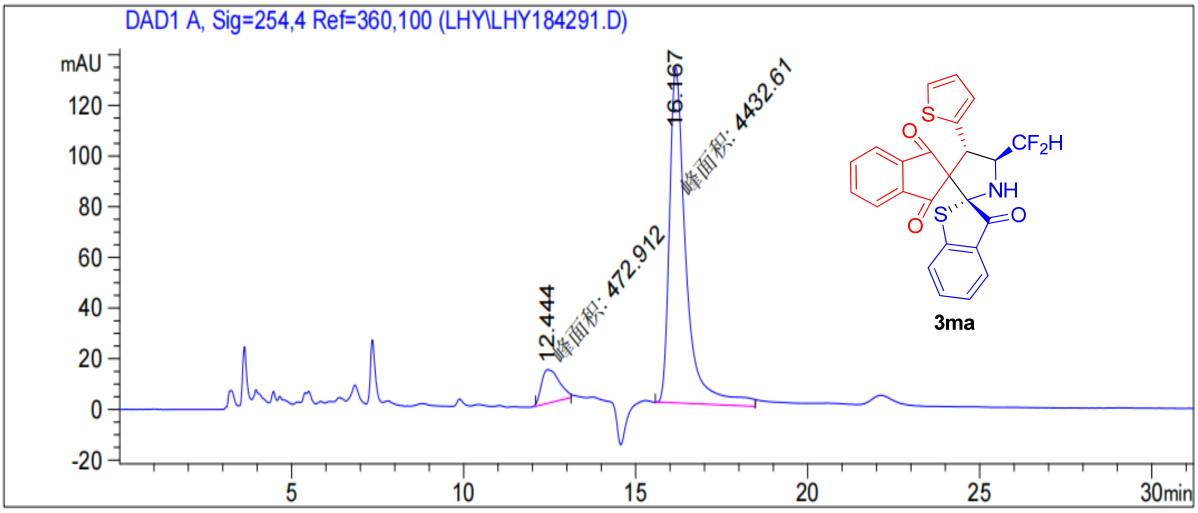

Peak #	RetTime [min]	Type	Width [min]	Area [mAU*s]	Height [mAU]	Area %
1	12.444	MM	0.5922	472.91208	13.30918	9.6404
2	16.167	MM	0.5584	4432.61230	132.31236	90.3596

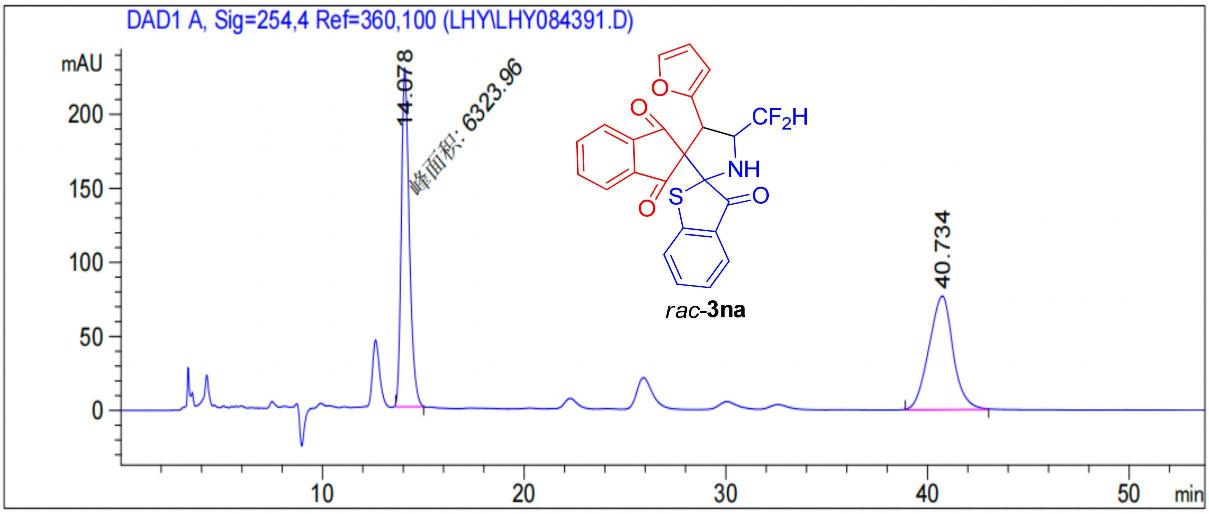

Peak #	RetTime [min]	Type	Width [min]	Area [mAU*s]	Height [mAU]	Area %
1	14.078	MM	0.4604	6323.96045	228.92690	49.6877
2	40.734	BB	1.2652	6403.45654	76.76231	50.3123

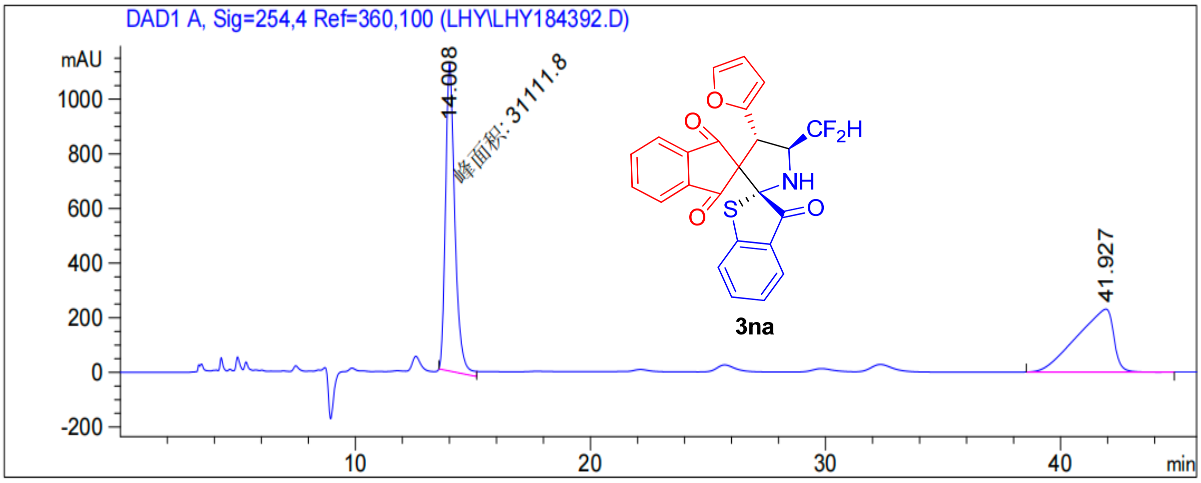

Peak #	RetTime [min]	Type	Width [min]	Area [mAU*s]	Height [mAU]	Area %
1	14.008	MM	0.4610	3.11118e4	1124.86938	55.2188
2	41.927	BB	1.4802	2.52309e4	230.65755	44.7812

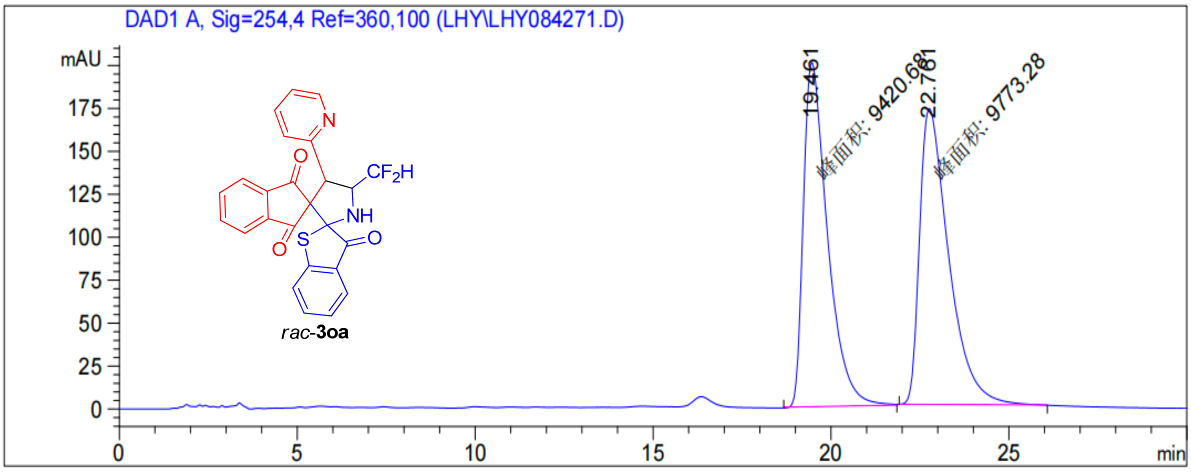

Peak #	RetTime [min]	Type	Width [min]	Area [mAU*s]	Height [mAU]	Area %
1	19.461	MM	0.7840	9420.68262	200.25690	49.0815
2	22.761	MM	0.9460	9773.27930	172.18600	50.9185

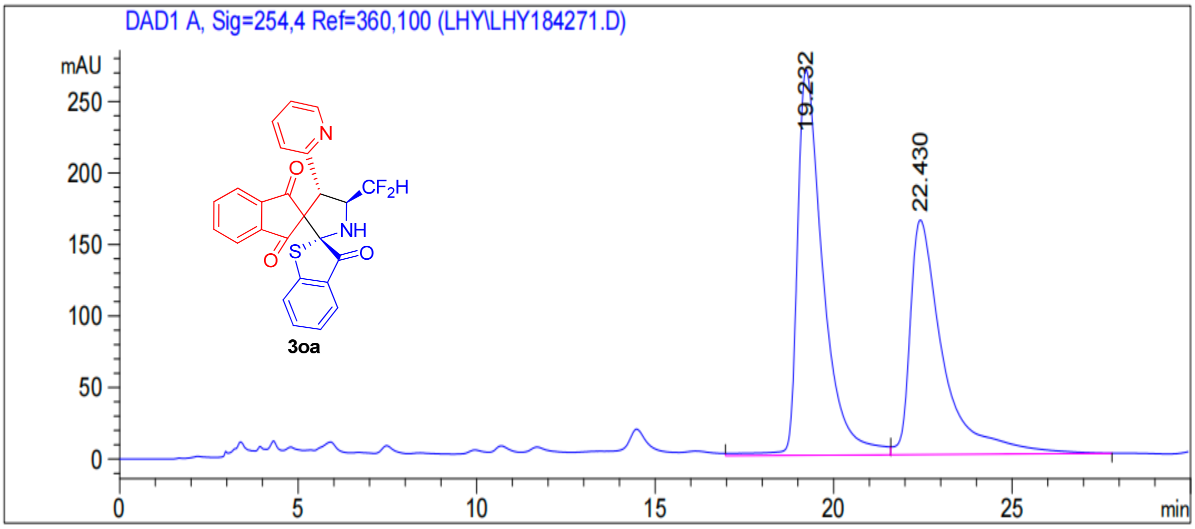

Peak #	RetTime [min]	Type	Width [min]	Area [mAU*s]	Height [mAU]	Area %
1	19.232	VV	0.7409	1.34158e4	269.43402	56.0099
2	22.430	VB	0.9421	1.05367e4	163.89340	43.9901

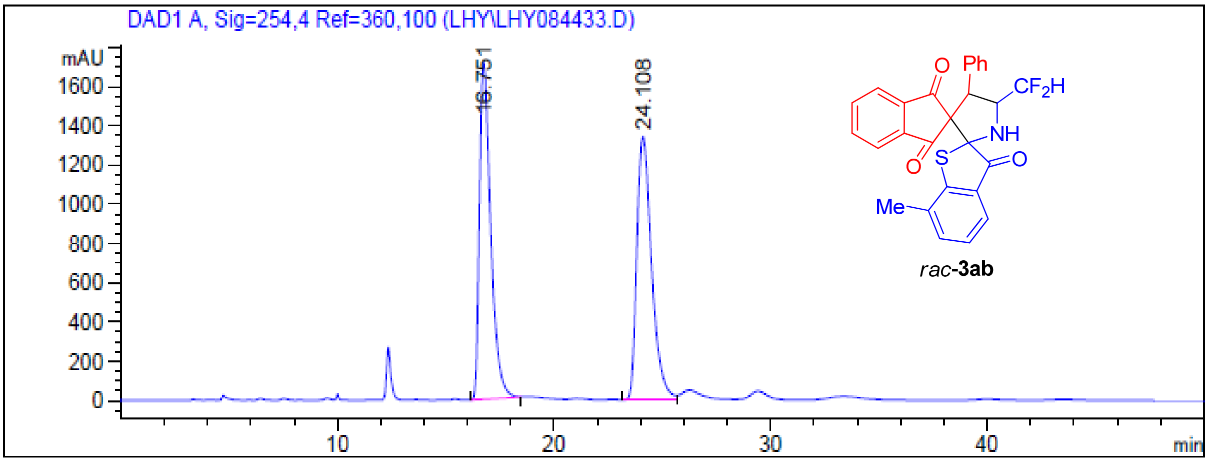

Peak #	RetTime [min]	Type	Width [min]	Area [mAU*s]	Height [mAU]	Area %
1	16.751	BB	0.5492	6.24109e4	1720.56824	49.4841
2	24.108	VV	0.7301	6.37121e4	1341.37866	50.5159

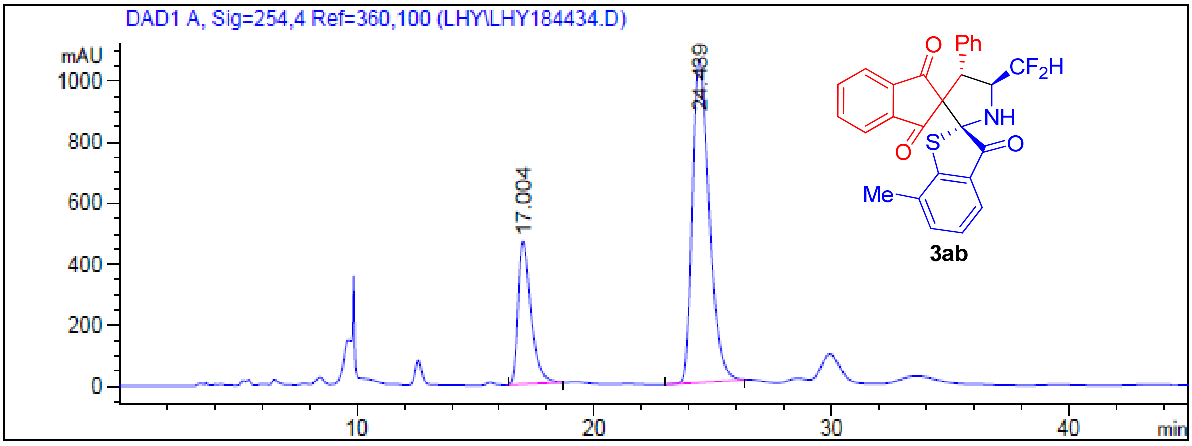

Peak #	RetTime [min]	Type	Width [min]	Area [mAU*s]	Height [mAU]	Area %
1	17.004	BB	0.5737	1.78117e4	468.36438	25.3462
2	24.439	BB	0.7616	5.24618e4	1059.63757	74.6538
